# Supplementary figures and images for: Uptake of infant and preschool immunisations in Scotland and England during the COVID-19 pandemic: An observational study of routinely collected data
Source: PLoS Med. 2022 Feb 22;19(2):e1003916. doi: 10.1371/journal.pmed.1003916 (PMC8863286; doi:10.1371/journal.pmed.1003916)

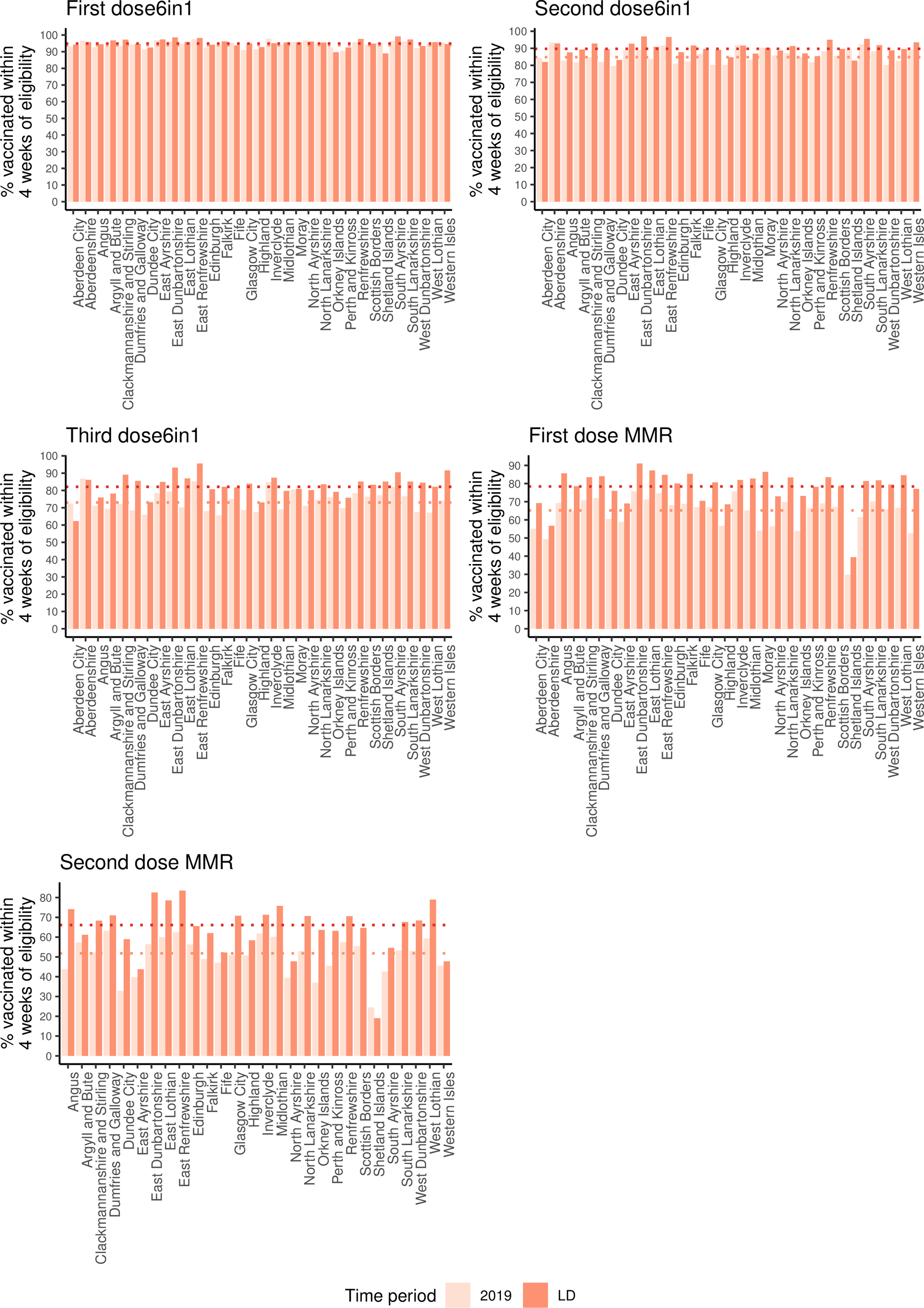

Supplement: S1 Fig — Percentage uptake by Scottish HSCP for 2019 (pale orange) and LD (dark orange) with HSCP ordered by uptake for 2019 (note: this varies by immunisation). Dashed horizontal lines indicated the mean uptake for all of Scotland for the time period of the corresponding colour. HSCP, Health and Social Care Partnership; LD, lockdown. (TIF) [file pmed.1003916.s001.tif]

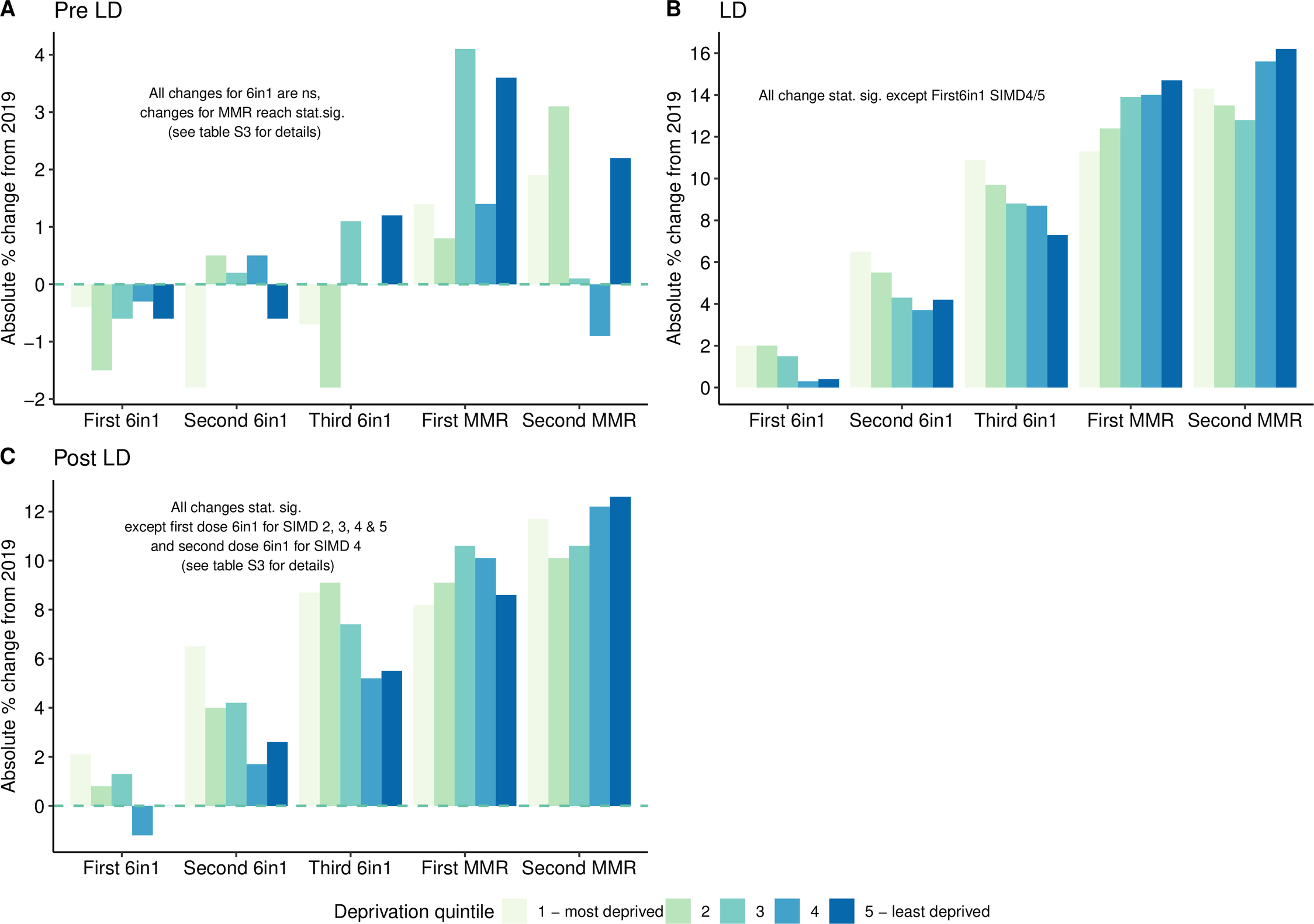

Supplement: S2 Fig — Absolute percentage change in uptake in Scotland compared to 2019 for each immunisations and SIMD for each time period (A = Pre LD, B = LD, C = Post LD). Significance rates varied by immunisation and SIMD; for details, see S3 Table. LD, lockdown; Post LD, postlockdown; Pre LD, prelockdown; SIMD, Scottish Index of Multiple Deprivation. (TIF) [file pmed.1003916.s002.tif]

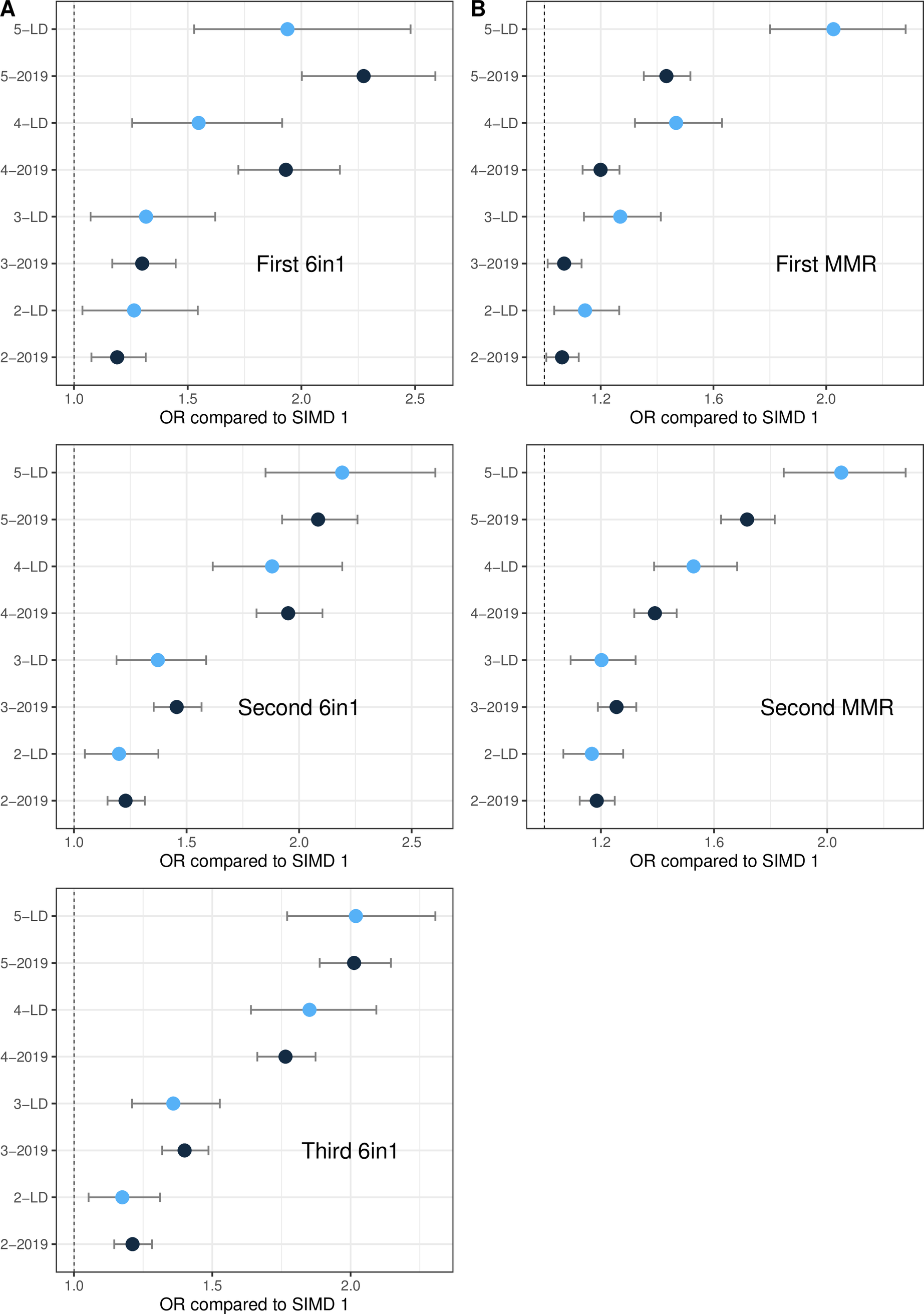

Supplement: S3 Fig — Combined OR plot with 95% CI comparing each SIMD quintile (2–5) to SIMD 1-most deprived for 2019 (dark blue) and LD (light blue). Data for Scotland only. CI, confidence interval; LD, lockdown; OR, odds ratio; SIMD, Scottish Index of Multiple Deprivation. (TIF) [file pmed.1003916.s003.tif]

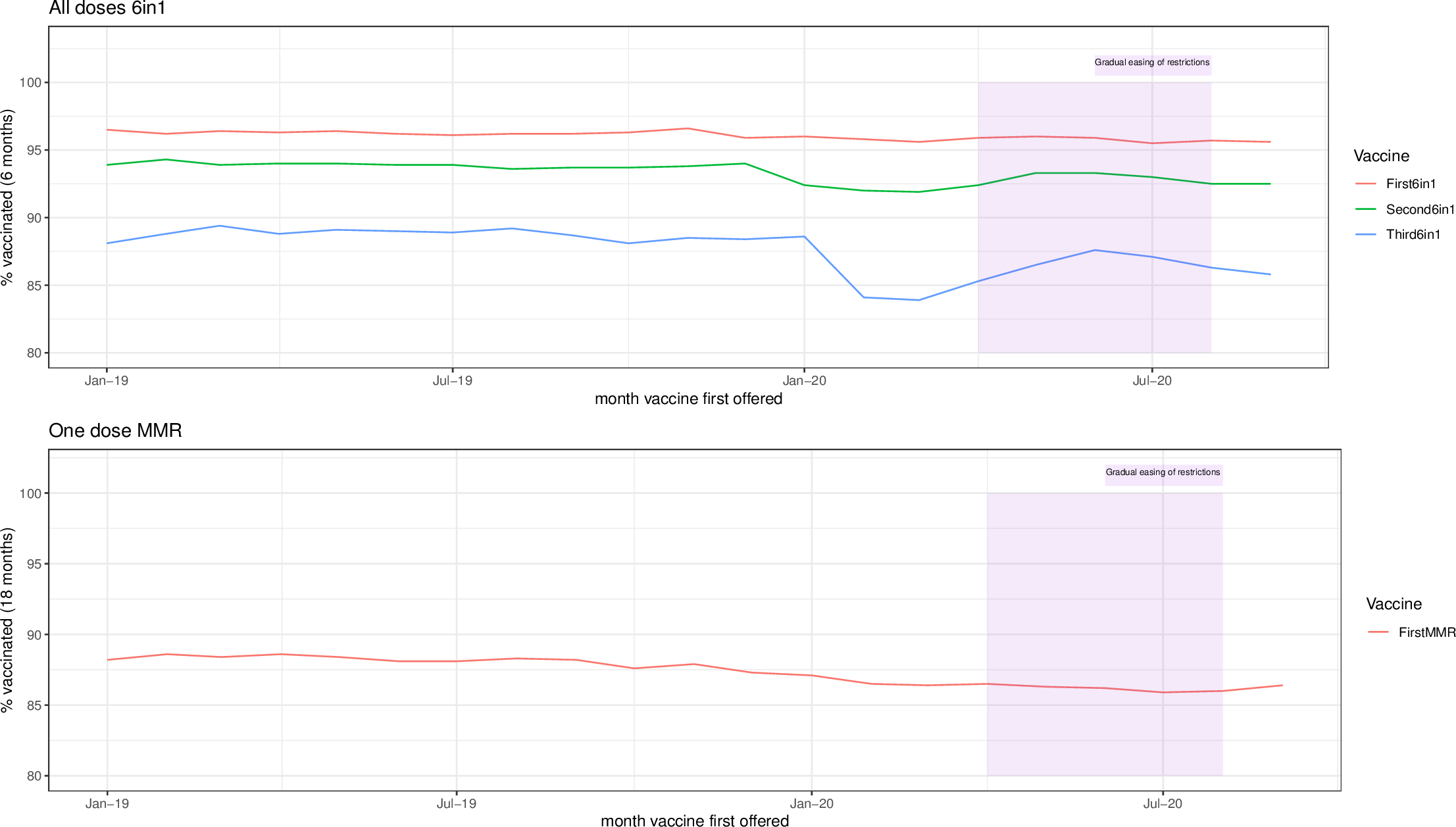

Supplement: S4 Fig — The start and end of the lockdown period is indicated by the purple shaded area. MMR, measles, mumps, and rubella. (TIF) [file pmed.1003916.s004.tif]
