# Supplementary material for: Uptake of infant and preschool immunisations in Scotland and England during the COVID-19 pandemic: An observational study of routinely collected data
Source: PLoS Med. 2022 Feb 22;19(2):e1003916. doi: 10.1371/journal.pmed.1003916 (PMC8863286; doi:10.1371/journal.pmed.1003916)
Supplement: S1 Table — Note this table gives an overview of the population as these data were not available for the individuals within the study. The date range encompasses the oldest to youngest children potentially included. For example, those born in 2015 would be eligible for the second dose MMR in 2019 and those born in early August 2020 would be eligible for the first dose 6-in-1 during the “postlockdown” time period. However, this does not account for children who may have migrated into/out of the areas since birth. Percentages are rounded to 1 decimal place. MMR, measles, mumps, and rubella. (DOCX) [file pmed.1003916.s005.docx]

| **Year** | **Region** | **Total births** | **Male**  **(%)** | **Female**  **(%)** | **Maternal age in years (%)** | | | | **Birthplace of Mother (%)** | | |
| --- | --- | --- | --- | --- | --- | --- | --- | --- | --- | --- | --- |
|  |  |  |  |  | **<25** | **25-34** | **>35** | **Unknown** | **UK** | **Non-UK** | **Unknown** |
| 2020 | Scotland | 46809 | 23972  (51.2) | 22837  (488) | 7482  (16.0) | 28274  (60.4) | 11047  (23.6) | 7  (0.0) | 38271  (81.7) | 8275  (17.7) | 263  (0.6) |
|  | England | 585195 | 299737  (51.2) | 285458  (48.8) | 89878  (15.4) | 353032  (60.3) | 142198  (24.3) | 87  (0.0) | 408717  (69.8) | 176448  (30.2) | 30  (0.0) |
| 2019 | Scotland | 49863 | 25687  (51.5) | 24176  (48.5) | 8156  (16.4) | 30136  (60.4) | 11565  (23.2) | 6  (0.0) | 41113  (82.5) | 8746  (17.5) | 4  (0.0) |
|  | England | 610505 | 313832  (51.4) | 296673  (48.6) | 98144  (16.1) | 365935  (59.9) | 146409  (24.0) | 17  (0.0) | 430098  (70.5) | 180370  (29.5) | 37  (0.0) |
| 2018 | Scotland | 51308 | 26332  (51.3) | 24976  (48.7) | 8801  (17.2) | 30738  (59.9) | 11767  (22.9) | 2  (0.0) | 42438  (82.7) | 8863  (17.3) | 7  (0.0) |
|  | England | 625651 | 321513  (51.4) | 304138  (48.6) | 103289  (16.5) | 374732  (59.9) | 147622  (23.6) | 8  (0.0) | 443762  (70.9) | 181859  (29.1) | 30  (0.0) |
| 2017 | Scotland | 52861 | 27215  (51.5) | 25646  (48.5) | 9558  (18.1) | 31492  (59.6) | 11810  (22.3) | 1  (0.0) | 43675  (82.6) | 9177  (17.4) | 9  (0.0) |
|  | England | 646794 | 331544  (51.3) | 315250  (48.7) | 110620  (17.1) | 387592  (59.9) | 148582  (23.0) | 0  (0.0) | 457930  (70.8) | 188829  (29.2) | 35  (0.0) |
| 2016 | Scotland | 54488 | 28236  (51.8) | 26252  (48.2) | 10256  (18.8) | 32466  (59.6) | 11749  (21.6) | 17  (0.0) | 45192  (82.9) | 9291  (17.1) | 5  (0.0) |
|  | England | 663157 | 340159  (51.3) | 322998  (48.7) | 117482  (17.7) | 396691  (59.8) | 148984  (22.5) | 0  (0.0) | 470759  (71.0) | 192355  (29.0) | 43  (0.0) |
| 2015 | Scotland | 55098 | 28354  (51.5) | 26744  (48.5) | 10769  (19.5) | 32768  (59.5) | 11553  (21.0) | 8  (0.0) | 46100  (83.7) | 8997  (16.3) | 1  (0.0) |
|  | England | 664399 | 341098  (51.3) | 323301  (48.7) | 124019  (18.7) | 396168  (59.6) | 144212  (21.7) | 0  (0.0) | 475995  (71.6) | 188369  (28.4) | 35  (0.0) |

Table S1: Summary statistics for all live births in Scotland England 2015-2020 from the National Records of Scotland (Scotland) (1) and Office of National Statistics (England) (2). Note this table gives an overview of the population as these data were not available for the individuals within the study. The date range encompasses the oldest to youngest children potentially included. For example, those born in 2015 would be eligible for the second dose MMR in 2019 and those born in early August 2020 would be eligible for the first dose 6in1 during the ‘Post lockdown’ time period. However, this does not account for children who may have migrated into/out of the areas since birth. Percentages are rounded to one decimal place.

1. National Records of Scotland. Vital Events Reference Tables: Births. National Records of Scotland; 2020. Available from https://www.nrscotland.gov.uk/statistics-and-data/statistics/statistics-by-theme/vital-events. Last acccessed 17^th^ October 2021.

2. National Office of Statistics. Nomis- Officaial labour Market Statistics: Live births in England and Wales by sex and characteristics of mother. 2021. Available from https://www.nomisweb.co.uk. Last acccessed 17^th^ October 2021.
