# Supplementary material for: Uptake of infant and preschool immunisations in Scotland and England during the COVID-19 pandemic: An observational study of routinely collected data
Source: PLoS Med. 2022 Feb 22;19(2):e1003916. doi: 10.1371/journal.pmed.1003916 (PMC8863286; doi:10.1371/journal.pmed.1003916)
Supplement: S2 Table — W/B, week beginning. (DOCX) [file pmed.1003916.s006.docx]

**Supplementary Table S2**

| **% uptake within 4 weeks of eligibility**  **(number received/total eligible)** | | | | | |
| --- | --- | --- | --- | --- | --- |
| **Time period** | **First 6in1** | **Second 6in1** | **Third 6in1** | **First MMR** | **Second MMR** |
| 2019 | 94  (47567/50609) | 84.8  (43221/50975) | 73  (37266/51083) | 65.2  (33935/52015) | 51.8  (25844/49940) |
| Jan-20 | 94.4  (3393/3593) | 84.1  (3101/3689) | 70.9  (2916/4112) | 68.4  (2578/3767) | 54.7  (2151/3934) |
| Feb-20 | 94.3  (4079/4325) | 86.7  (3878/4472) | 74  (3403/4600) | 69.8  (3309/4739) | 59.2  (2845/4804) |
| W/B 02-MAR-20 | 92.8  (813/876) | 84.6  (729/862) | 73.4  (628/856) | 67.2  (636/947) | 54.3  (491/904) |
| W/B 09-MAR-20 | 93.4  (883/945) | 84.3  (763/905) | 72.4  (624/862) | 66.1  (656/993) | 51  (474/930) |
| W/B 16-MAR-20 | 91.5  (935/1022) | 82.5  (635/770) | 73.1  (705/964) | 65.3  (603/924) | 46.5  (429/923) |
| W/B 23-MAR-20 | 93.2  (846/908) | 83.7  (761/909) | 73.3  (644/879) | 64.4  (588/913) | 46.9  (430/916) |
| W/B 30-MAR-20 | 95  (864/909) | 85.5  (749/876) | 75.5  (651/862) | 71.1  (692/973) | 48.6  (472/972) |
| W/B 06-APR-20 | 92.6  (892/963) | 86.9  (821/945) | 76  (688/905) | 69.7  (636/912) | 56  (509/909) |
| W/B 13-APR-20 | 93.9  (835/889) | 87.2  (891/1022) | 77  (593/770) | 76.9  (749/974) | 58.4  (558/955) |
| W/B 20-APR-20 | 95.1  (851/895) | 88.7  (805/908) | 78  (709/909) | 77.5  (732/945) | 63.3  (567/896) |
| W/B 27-APR-20 | 96  (881/918) | 91  (827/909) | 79.8  (699/876) | 80.3  (789/982) | 65.6  (563/858) |
| W/B 04-MAY-20 | 94.7  (838/885) | 88.1  (848/963) | 80  (756/945) | 80.4  (781/971) | 66.9  (590/882) |
| W/B 11-MAY-20 | 96.3  (894/928) | 89.1  (792/889) | 82.7  (845/1022) | 82.8  (815/984) | 70.6  (653/925) |
| W/B 18-MAY-20 | 95.5  (857/897) | 91.6  (820/895) | 84.7  (769/908) | 81.3  (784/964) | 70.6  (602/853) |
| W/B 25-MAY-20 | 96.2  (840/873) | 92.7  (851/918) | 86.1  (783/909) | 83.2  (820/986) | 74.2  (636/857) |
| W/B 01-JUN-20 | 97.1  (835/860) | 91  (805/885) | 83.1  (800/963) | 80.9  (728/900) | 73.1  602/824) |
| W/B 08-JUN-20 | 95  (899/946) | 93.5  (868/928) | 82.9  (737/889) | 81.9  (801/978) | 76.5  (739/966) |
| W/B 15-JUN-20 | 95.9  (880/918) | 91  (816/897) | 84.7  (758/895) | 81.7  (793/971) | 70.8  (644/909) |
| W/B 22-JUN-20 | 95.5  (804/842) | 92.1  (804/873) | 85.6  (786/918) | 80.6  (789/979) | 70.4  (650/923) |
| W/B 29-JUN-20 | 94.8  (795/839) | 92.9  (799/860) | 86.6  (766/885) | 79.3  (783/987) | 68.1  (608/893) |
| W/B 06-JUL-20 | 96.4  (863/895) | 90.5  (856/946) | 87.6  (813/928) | 76.8  (788/1026) | 71.4  (637/892) |
| W/B 13-JUL-20 | 95.2  (894/939) | 90.8  (834/918) | 85.1  (763/897) | 80.4  (805/1001) | 69.5  (629/905) |
| W/B 20-JUL-20 | 96.8  (864/893) | 89  (749/842) | 85.1  (743/873) | 80  (813/1016) | 68.2  (597/875) |
| W/B 27-JUL-20 | 94.6  (885/936) | 89  (747/839) | 85.6  (736/860) | 79.5  (796/1001) | 66  (617/935) |
| W/B 03-AUG-20 | 95  (916/964) | 90.4  (809/895) | 83.2  (787/946) | 78.3  (808/1032) | 70  (656/937) |
| W/B 10-AUG-20 | 95.1  (847/891) | 89.1  (837/939) | 82.4  (756/918) | 77.2  (761/986) | 65.9  (602/913) |
| W/B 17-AUG-20 | 95  (914/962) | 89.5  (799/893) | 80.6  (679/842) | 75.9  (749/987) | 65.8  (571/868) |
| W/B 24-AUG-20 | 94.7  (946/999) | 87.1  (815/936) | 81.3  (682/839) | 73.8  (721/977) | 61.4  (551/897) |
| W/B 31-AUG-20 | 94.9  (885/933) | 88.4  (852/964) | 81.2  (727/895) | 74  (709/958) | 61  (540/885) |
| W/B 07-SEP-20 | 95.1  (939/987) | 89.1  (794/891) | 79.7  (748/939) | 72.9  (725/995) | 63.5  (589/927) |
| W/B 14-SEP-20 | 94  (885/941) | 87.6  (843/962) | 79.3  (708/893) | 74  (751/1015) | 61.9  (569/919) |
| W/B 21-SEP-20 | 93.5  (864/924) | 88.3  (882/999) | 77.4  (724/936) | 73.4  (782/1066) | 58.8  (549/933) |
| W/B 28-SEP-20 | 94.5  (879/930) | 88.7  (828/933) | 77.2  (744/964) | 71.2  (734/1031) | 59.3  (544/917) |

Table S2: Percentage uptake of each immunisation by year (2019), month (Jan and Feb 2020) or week as per data availability. W/B = week beginning.
