# Supplementary material for: Uptake of infant and preschool immunisations in Scotland and England during the COVID-19 pandemic: An observational study of routinely collected data
Source: PLoS Med. 2022 Feb 22;19(2):e1003916. doi: 10.1371/journal.pmed.1003916 (PMC8863286; doi:10.1371/journal.pmed.1003916)
Supplement: S3 Table — A-E: Percentage uptake, percent point change in uptake compared to 2019 and significance level for this change for each HSCP at each time period. Each table shows results for a different immunisation. p-Values calculated using aggregate binary logistic regression and rounded to 2 decimal places. Results were considered significant if p-value <0.05 and 95% CI did not include 1. Statistically significant p-values are shaded green, and significant results for the 2019–LD comparisons are plotted on Fig 2. CI, confidence interval; HSCP, Health and Social Care Partnership; LD, lockdown; NA, not applicable; OR, odds ratio; PostLD, postlockdown; PreLD, pre lockdown. (DOCX) [file pmed.1003916.s007.docx]

**Supplementary Tables S3**

A-E Percentage uptake, percent point change in uptake compared to 2019 and significance level for this change for each HSCP at each time-period. Each table shows results for a different immunisation. *p*-values calculated using aggregate binary logistic regression and rounded to 2 decimal places. Results were considered significant if *p*-value <0.05 and 95% CI did not include 1. Statistically significant p values are shaded green and significant results for the 2019-LD comparisons are plotted on Figure 2. HSCP = Health and Social Care Partnership, OR = odds ratio, CI = confidence interval, NA = not applicable, PreLD = pre lockdown, LD = lockdown, PostLD = post lockdown.

**A First dose 6in1**

| **HSCP** | **Time period** | **% uptake (within 4 weeks)** | **Number received** | **Number eligible** | **% point change from 2019** | **OR compared to 2019 (95% CI)** | **p value** |
| --- | --- | --- | --- | --- | --- | --- | --- |
| Aberdeen City | 2019 | 93.2 | 2077 | 2228 | NA | NA | NA |
|  | PreLD | 90.5 | 382 | 425 | -2.7 | 0.65  (0.45-0.92) | 0.02 |
|  | LD | 94.2 | 696 | 739 | 1 | 1.18  (0.83-1.67) | 0.36 |
|  | PostLD | 91.6 | 334 | 365 | -1.6 | 0.78  (0.52-1.17) | 0.24 |
| Aberdeenshire | 2019 | 96.6 | 2452 | 2538 | NA | NA | NA |
|  | PreLD | 95.8 | 513 | 540 | -0.8 | 0.67  (0.43-1.04) | 0.07 |
|  | LD | 95.7 | 793 | 830 | -0.9 | 0.75  (0.51-1.11) | 0.16 |
|  | PostLD | 92.1 | 354 | 385 | -4.5 | 0.4  (0.26-0.61) | <0.001 |
| Angus | 2019 | 92.7 | 939 | 1013 | NA | NA | NA |
|  | PreLD | 92.4 | 205 | 219 | -0.3 | 1.15  (0.64-2.08) | 0.63 |
|  | LD | 94.5 | 312 | 330 | 1.8 | 1.37  (0.8-2.32) | 0.25 |
|  | PostLD | 93.9 | 158 | 169 | 1.2 | 1.13  (0.59-2.18) | 0.71 |
| Argyll and Bute | 2019 | 93.3 | 608 | 652 | NA | NA | NA |
|  | PreLD | 93.4 | 140 | 150 | 0.1 | 1.01  (0.5-2.06) | 0.97 |
|  | LD | 96.8 | 234 | 243 | 3.5 | 1.88  (0.9-3.92) | 0.09 |
|  | PostLD | 91.3 | 96 | 106 | -2 | 0.69  (0.34-1.43) | 0.32 |
| Clackmannanshire and Stirling | 2019 | 93.3 | 1092 | 1170 | NA | NA | NA |
|  | PreLD | 92.9 | 272 | 288 | -0.4 | 1.21  (0.7-2.11) | 0.49 |
|  | LD | 97.3 | 398 | 408 | 4 | 2.84  (1.46-5.55) | <0.001 |
|  | PostLD | 95.9 | 191 | 199 | 2.6 | 1.71  (0.81-3.59) | 0.16 |
| Dumfries and Galloway | 2019 | 94.4 | 1116 | 1182 | NA | NA | NA |
|  | PreLD | 91.1 | 231 | 247 | -3.3 | 0.85  (0.49-1.5) | 0.58 |
|  | LD | 94.6 | 404 | 429 | 0.2 | 0.96  (0.59-1.54) | 0.85 |
|  | PostLD | 93.7 | 194 | 206 | -0.7 | 0.96  (0.51-1.8) | 0.89 |
| Dundee City | 2019 | 91.4 | 1289 | 1411 | NA | NA | NA |
|  | PreLD | 89.1 | 266 | 290 | -2.3 | 1.05  (0.66-1.66) | 0.84 |
|  | LD | 92.5 | 426 | 461 | 1.1 | 1.15  (0.78-1.7) | 0.48 |
|  | PostLD | 92.1 | 206 | 225 | 0.7 | 1.03  (0.62-1.7) | 0.92 |
| East Ayrshire | 2019 | 96.9 | 1122 | 1158 | NA | NA | NA |
|  | PreLD | 95.1 | 241 | 252 | -1.8 | 0.7  (0.35-1.4) | 0.32 |
|  | LD | 97.4 | 420 | 431 | 0.5 | 1.23  (0.62-2.43) | 0.56 |
|  | PostLD | 94.1 | 181 | 193 | -2.8 | 0.48  (0.25-0.95) | 0.03 |
| East Dunbartonshire | 2019 | 96.1 | 958 | 997 | NA | NA | NA |
|  | PreLD | 95.7 | 187 | 193 | -0.4 | 1.27  (0.53-3.04) | 0.59 |
|  | LD | 98.6 | 339 | 344 | 2.5 | 2.76  (1.08-7.06) | 0.03 |
|  | PostLD | 99.4 | 154 | 155 | 3.3 | 6.27  (0.86-45.96) | 0.07 |
| East Lothian | 2019 | 94.6 | 1031 | 1090 | NA | NA | NA |
|  | PreLD | 93.9 | 194 | 204 | -0.7 | 1.11  (0.56-2.21) | 0.77 |
|  | LD | 95.8 | 330 | 346 | 1.2 | 1.18  (0.67-2.08) | 0.57 |
|  | PostLD | 95.3 | 194 | 203 | 0.7 | 1.23  (0.6-2.53) | 0.57 |
| East Renfrewshire | 2019 | 97.4 | 868 | 891 | NA | NA | NA |
|  | PreLD | 96 | 193 | 201 | -1.4 | 0.64  (0.28-1.45) | 0.28 |
|  | LD | 98.3 | 290 | 295 | 0.9 | 1.54  (0.58-4.08) | 0.39 |
|  | PostLD | 97.4 | 149 | 153 | 0 | 0.99  (0.34-2.89) | 0.98 |
| Edinburgh | 2019 | 92.5 | 4130 | 4464 | NA | NA | NA |
|  | PreLD | 89.9 | 883 | 964 | -2.6 | 0.88  (0.68-1.14) | 0.33 |
|  | LD | 94.2 | 1517 | 1613 | 1.7 | 1.28  (1.01-1.62) | 0.04 |
|  | PostLD | 94.4 | 751 | 796 | 1.9 | 1.35  (0.98-1.86) | 0.07 |
| Falkirk | 2019 | 93.7 | 1382 | 1475 | NA | NA | NA |
|  | PreLD | 95 | 291 | 308 | 1.3 | 1.15  (0.68-1.96) | 0.6 |
|  | LD | 96.2 | 513 | 534 | 2.5 | 1.64  (1.01-2.67) | 0.04 |
|  | PostLD | 95.8 | 245 | 256 | 2.1 | 1.5  (0.79-2.84) | 0.21 |
| Fife | 2019 | 95.2 | 3234 | 3396 | NA | NA | NA |
|  | PreLD | 93.4 | 700 | 744 | -1.8 | 0.8  (0.57-1.12) | 0.19 |
|  | LD | 93.7 | 1078 | 1150 | -1.5 | 0.75  (0.56-1) | 0.05 |
|  | PostLD | 92.3 | 520 | 562 | -2.9 | 0.62  (0.44-0.88) | 0.01 |
| Glasgow City | 2019 | 91 | 5855 | 6435 | NA | NA | NA |
|  | PreLD | 92.5 | 1254 | 1346 | 1.5 | 1.35  (1.07-1.7) | 0.01 |
|  | LD | 95 | 2029 | 2136 | 4 | 1.88  (1.52-2.32) | <0.001 |
|  | PostLD | 94.8 | 1069 | 1128 | 3.8 | 1.79  (1.36-2.36) | <0.001 |
| Highland | 2019 | 91.4 | 1821 | 1992 | NA | NA | NA |
|  | PreLD | 88.8 | 389 | 433 | -2.6 | 0.83  (0.59-1.18) | 0.3 |
|  | LD | 92.8 | 587 | 635 | 1.4 | 1.15  (0.82-1.6) | 0.42 |
|  | PostLD | 92.9 | 336 | 360 | 1.5 | 1.31  (0.84-2.05) | 0.23 |
| Inverclyde | 2019 | 97.8 | 618 | 632 | NA | NA | NA |
|  | PreLD | 96.9 | 146 | 151 | -0.9 | 0.66  (0.23-1.87) | 0.43 |
|  | LD | 95.1 | 217 | 227 | -2.7 | 0.49  (0.22-1.12) | 0.09 |
|  | PostLD | 97.1 | 96 | 99 | -0.7 | 0.72  (0.2-2.57) | 0.62 |
| Midlothian | 2019 | 94.1 | 1051 | 1117 | NA | NA | NA |
|  | PreLD | 94.4 | 212 | 226 | 0.3 | 0.95  (0.52-1.72) | 0.87 |
|  | LD | 95.5 | 336 | 352 | 1.4 | 1.32  (0.75-2.31) | 0.33 |
|  | PostLD | 95.8 | 191 | 200 | 1.7 | 1.33  (0.65-2.72) | 0.43 |
| Moray | 2019 | 95.7 | 788 | 823 | NA | NA | NA |
|  | PreLD | 95.2 | 209 | 222 | -0.5 | 0.71  (0.37-1.37) | 0.31 |
|  | LD | 95.6 | 295 | 310 | -0.1 | 0.87  (0.47-1.62) | 0.67 |
|  | PostLD | 97.4 | 141 | 145 | 1.7 | 1.57  (0.55-4.47) | 0.4 |
| North Ayrshire | 2019 | 96.8 | 1082 | 1118 | NA | NA | NA |
|  | PreLD | 98.2 | 261 | 270 | 1.4 | 0.96  (0.46-2.03) | 0.92 |
|  | LD | 96 | 371 | 386 | -0.8 | 0.82  (0.45-1.52) | 0.53 |
|  | PostLD | 93.7 | 182 | 193 | -3.1 | 0.55  (0.28-1.1) | 0.09 |
| North Lanarkshire | 2019 | 95.1 | 3337 | 3510 | NA | NA | NA |
|  | PreLD | 95.6 | 696 | 724 | 0.5 | 1.29  (0.86-1.94) | 0.22 |
|  | LD | 95.5 | 1049 | 1098 | 0.4 | 1.11  (0.8-1.54) | 0.53 |
|  | PostLD | 97.1 | 609 | 627 | 2 | 1.75  (1.07-2.87) | 0.03 |
| Orkney Islands | 2019 | 92.6 | 189 | 204 | NA | NA | NA |
|  | PreLD | 100 | 28 | 28 | 7.4 | 1.16  (0.55-2.44) | 0.69 |
|  | LD | 89.7 | 53 | 59 | -2.9 | 1.27  (0.81-2.01) | 0.3 |
|  | PostLD | 100 | 37 | 37 | 7.4 | 0.8  (0.48-1.33) | 0.39 |
| Perth and Kinross | 2019 | 90.6 | 1124 | 1240 | NA | NA | NA |
|  | PreLD | 87.4 | 225 | 255 | -3.2 | 0.77  (0.51-1.19) | 0.24 |
|  | LD | 92.5 | 411 | 444 | 1.9 | 1.29  (0.86-1.92) | 0.22 |
|  | PostLD | 93.2 | 191 | 206 | 2.6 | 1.31  (0.75-2.3) | 0.34 |
| Renfrewshire | 2019 | 95.7 | 1617 | 1689 | NA | NA | NA |
|  | PreLD | 97.8 | 377 | 385 | 2.1 | 2.1  (1-4.39) | 0.05 |
|  | LD | 97.6 | 587 | 602 | 1.9 | 1.74  (0.99-3.06) | 0.05 |
|  | PostLD | 96.6 | 239 | 248 | 0.9 | 1.18  (0.58-2.4) | 0.64 |
| Scottish Borders | 2019 | 94.3 | 838 | 889 | NA | NA | NA |
|  | PreLD | 93.9 | 183 | 193 | -0.4 | 1.11  (0.56-2.23) | 0.76 |
|  | LD | 94.9 | 291 | 306 | 0.6 | 1.18  (0.65-2.13) | 0.58 |
|  | PostLD | 95.8 | 142 | 148 | 1.5 | 1.44  (0.61-3.42) | 0.41 |
| Shetland Islands | 2019 | 95.3 | 203 | 213 | NA | NA | NA |
|  | PreLD | 86.4 | 22 | 25 | -8.9 | 0.36  (0.09-1.41) | 0.14 |
|  | LD | 89 | 54 | 60 | -6.3 | 1.27  (0.81-2.01) | 0.3 |
|  | PostLD | 89.6 | 33 | 37 | -5.8 | 0.8  (0.48-1.33) | 0.39 |
| South Ayrshire | 2019 | 96.5 | 844 | 875 | NA | NA | NA |
|  | PreLD | 94.1 | 187 | 198 | -2.4 | 0.62  (0.31-1.26) | 0.19 |
|  | LD | 99.2 | 274 | 277 | 2.7 | 3.35  (1.02-11.06) | 0.05 |
|  | PostLD | 97.4 | 148 | 152 | 0.9 | 1.36  (0.47-3.91) | 0.57 |
| South Lanarkshire | 2019 | 96 | 3066 | 3194 | NA | NA | NA |
|  | PreLD | 94.6 | 622 | 646 | -1.4 | 1.08  (0.69-1.69) | 0.73 |
|  | LD | 97.4 | 1084 | 1113 | 1.4 | 1.56  (1.04-2.35) | 0.03 |
|  | PostLD | 96.4 | 484 | 502 | 0.4 | 1.12  (0.68-1.86) | 0.65 |
| West Dunbartonshire | 2019 | 92.1 | 796 | 864 | NA | NA | NA |
|  | PreLD | 90.3 | 158 | 172 | -1.8 | 0.96  (0.53-1.76) | 0.9 |
|  | LD | 93.4 | 274 | 292 | 1.3 | 1.3  (0.76-2.23) | 0.34 |
|  | PostLD | 95.8 | 144 | 150 | 3.7 | 2.05  (0.87-4.81) | 0.1 |
| West Lothian | 2019 | 94.7 | 1754 | 1852 | NA | NA | NA |
|  | PreLD | 93.9 | 342 | 363 | -0.8 | 0.91  (0.56-1.48) | 0.7 |
|  | LD | 95.7 | 547 | 571 | 1 | 1.27  (0.81-2.01) | 0.3 |
|  | PostLD | 93.4 | 272 | 291 | -1.3 | 0.8  (0.48-1.33) | 0.39 |
| Western Isles | 2019 | 95.4 | 188 | 197 | NA | NA | NA |
|  | PreLD | 95.8 | 33 | 35 | 0.4 | 0.79  (0.16-3.82) | 0.77 |
|  | LD | 94.6 | 56 | 59 | -0.8 | 1.18  (0.83-1.67) | 0.36 |
|  | PostLD | 95.4 | 23 | 24 | 0 | 0.78  (0.52-1.17) | 0.24 |

**B Second dose 6in1**

| **HSCP** | **Time period** | **% uptake (within 4 weeks)** | **Number received** | **Number eligible** | **% point change from 2019** | **OR compared to 2019**  **(95% CI)** | **p value** |
| --- | --- | --- | --- | --- | --- | --- | --- |
| Aberdeen City | 2019 | 84.3 | 1891 | 2244 | NA | NA | NA |
|  | PreLD | 74.7 | 358 | 459 | -9.6 | 0.66  (0.52-0.85) | <0.001 |
|  | LD | 81.9 | 603 | 733 | -2.4 | 0.87  (0.69-1.08) | 0.2 |
|  | PostLD | 82 | 281 | 344 | -2.3 | 0.83  (0.62-1.12) | 0.23 |
| Aberdeenshire | 2019 | 93.3 | 2411 | 2584 | NA | NA | NA |
|  | PreLD | 87.4 | 470 | 530 | -5.9 | 0.56  (0.41-0.77) | <0.001 |
|  | LD | 92.9 | 753 | 811 | -0.4 | 0.93  (0.68-1.27) | 0.65 |
|  | PostLD | 90.7 | 365 | 404 | -2.6 | 0.67  (0.47-0.97) | 0.03 |
| Angus | 2019 | 82.7 | 843 | 1019 | NA | NA | NA |
|  | PreLD | 80 | 188 | 223 | -2.7 | 1.12  (0.75-1.67) | 0.57 |
|  | LD | 87.6 | 288 | 330 | 4.9 | 1.43  (1-2.06) | 0.05 |
|  | PostLD | 85.7 | 136 | 160 | 3 | 1.18  (0.74-1.88) | 0.48 |
| Argyll and Bute | 2019 | 81.7 | 523 | 640 | NA | NA | NA |
|  | PreLD | 84.4 | 137 | 167 | 2.7 | 1.02  (0.66-1.59) | 0.92 |
|  | LD | 89.3 | 214 | 240 | 7.6 | 1.84  (1.17-2.9) | 0.01 |
|  | PostLD | 83.8 | 86 | 107 | 2.1 | 0.92  (0.55-1.54) | 0.74 |
| Clackmannanshire and Stirling | 2019 | 84.9 | 1003 | 1182 | NA | NA | NA |
|  | PreLD | 91.2 | 231 | 259 | 6.3 | (1.47  (0.96-2.25) | 0.07 |
|  | LD | 92.8 | 416 | 448 | 7.9 | 2.32  (1.57-3.44) | <0.001 |
|  | PostLD | 92.5 | 173 | 188 | 7.6 | 2.06  (1.19-3.57) | 0.01 |
| Dumfries and Galloway | 2019 | 82 | 978 | 1193 | NA | NA | NA |
|  | PreLD | 87.2 | 217 | 251 | 5.2 | 1.4  (0.95-2.07) | 0.09 |
|  | LD | 89.5 | 363 | 409 | 7.5 | 1.73  (1.23-2.44) | <0.001 |
|  | PostLD | 90.3 | 197 | 218 | 8.3 | 2.06  (1.28-3.31) | <0.001 |
| Dundee City | 2019 | 79.6 | 1133 | 1423 | NA | NA | NA |
|  | PreLD | 75.9 | 226 | 284 | -3.7 | 1  (0.73-1.37) | 0.99 |
|  | LD | 83.2 | 385 | 463 | 3.6 | 1.26  (0.96-1.66) | 0.1 |
|  | PostLD | 83.9 | 199 | 238 | 4.3 | 1.31  (0.91-1.88) | 0.15 |
| East Ayrshire | 2019 | 89 | 1052 | 1182 | NA | NA | NA |
|  | PreLD | 88.2 | 214 | 242 | -0.8 | 0.94  (0.61-1.46) | 0.8 |
|  | LD | 92.7 | 400 | 429 | 3.7 | 1.7  (1.12-2.59) | 0.01 |
|  | PostLD | 92.8 | 187 | 202 | 3.8 | 1.54  (0.88-2.69) | 0.13 |
| East Dunbartonshire | 2019 | 88.9 | 893 | 1005 | NA | NA | NA |
|  | PreLD | 96.4 | 186 | 196 | 7.5 | 2.33  (1.2-4.54) | 0.01 |
|  | LD | 97 | 331 | 342 | 8.1 | 3.77  (2.01-7.1) | <0.001 |
|  | PostLD | 95.4 | 168 | 176 | 6.5 | 2.63  (1.26-5.5) | 0.01 |
| East Lothian | 2019 | 83.8 | 932 | 1112 | NA | NA | NA |
|  | PreLD | 87.1 | 169 | 198 | 3.3 | 1.13  (0.74-1.72) | 0.59 |
|  | LD | 90.9 | 326 | 360 | 7.1 | 1.85  (1.26-2.73) | <0.001 |
|  | PostLD | 88.8 | 152 | 170 | 5 | 1.63  (0.98-2.73) | 0.06 |
| East Renfrewshire | 2019 | 91.6 | 834 | 910 | NA | NA | NA |
|  | PreLD | 95.8 | 171 | 181 | 4.2 | 1.56  (0.79-3.07) | 0.2 |
|  | LD | 96.6 | 282 | 292 | 5 | 2.57  (1.31-5.04) | 0.01 |
|  | PostLD | 96.3 | 156 | 162 | 4.7 | 2.37  (1.01-5.54) | 0.05 |
| Edinburgh | 2019 | 81 | 3650 | 4505 | NA | NA | NA |
|  | PreLD | 83.3 | 814 | 980 | 2.3 | 1.15  (0.96-1.38) | 0.14 |
|  | LD | 87.7 | 1387 | 1582 | 6.7 | 1.67  (1.41-1.97) | <0.001 |
|  | PostLD | 83.9 | 679 | 810 | 2.9 | 1.21  (0.99-1.48) | 0.06 |
| Falkirk | 2019 | 82.3 | 1243 | 1511 | NA | NA | NA |
|  | PreLD | 82.8 | 260 | 315 | 0.5 | 1.02  (0.74-1.4) | 0.91 |
|  | LD | 91.7 | 473 | 517 | 9.4 | 2.32  (1.66-3.24) | <0.001 |
|  | PostLD | 92.1 | 231 | 250 | 9.8 | 2.62  (1.61-4.26) | <0.001 |
| Fife | 2019 | 86.8 | 2966 | 3418 | NA | NA | NA |
|  | PreLD | 83.7 | 627 | 732 | -3.1 | 0.91  (0.72-1.14) | 0.42 |
|  | LD | 89.6 | 1030 | 1151 | 2.8 | 1.3  (1.05-1.6) | 0.02 |
|  | PostLD | 90.2 | 509 | 562 | 3.4 | 1.46  (1.08-1.98) | 0.01 |
| Glasgow City | 2019 | 80.2 | 5196 | 6480 | NA | NA | NA |
|  | PreLD | 83.2 | 1116 | 1347 | 3 | 1.19  (1.02-1.39) | 0.02 |
|  | LD | 89.1 | 1894 | 2125 | 8.9 | 2.03  (1.74-2.35) | <0.001 |
|  | PostLD | 90.8 | 971 | 1069 | 10.6 | 2.45  (1.97-3.04) | <0.001 |
| Highland | 2019 | 80.3 | 1600 | 1992 | NA | NA | NA |
|  | PreLD | 75.5 | 332 | 419 | -4.8 | 0.93  (0.72-1.21) | 0.61 |
|  | LD | 84.8 | 565 | 669 | 4.5 | 1.33  (1.05-1.69) | 0.02 |
|  | PostLD | 80.1 | 258 | 321 | -0.2 | 1  (0.75-1.35) | 0.98 |
| Inverclyde | 2019 | 91.9 | 575 | 626 | NA | NA | NA |
|  | PreLD | 91.2 | 142 | 156 | -0.7 | 0.9  (0.48-1.67) | 0.74 |
|  | LD | 91.6 | 207 | 226 | -0.3 | 0.97  (0.56-1.68) | 0.9 |
|  | PostLD | 88.7 | 91 | 101 | -3.2 | 0.81  (0.4-1.65) | 0.56 |
| Midlothian | 2019 | 83.3 | 935 | 1122 | NA | NA | NA |
|  | PreLD | 84.1 | 181 | 218 | 0.8 | 0.98  (0.66-1.44) | 0.91 |
|  | LD | 86.9 | 308 | 354 | 3.6 | 1.34  (0.95-1.9) | 0.1 |
|  | PostLD | 93.9 | 172 | 183 | 10.6 | 3.13  (1.67-5.87) | <0.001 |
| Moray | 2019 | 90.4 | 751 | 831 | NA | NA | NA |
|  | PreLD | 88.8 | 171 | 197 | -1.6 | 0.7  (0.44-1.12) | 0.14 |
|  | LD | 90.2 | 279 | 311 | -0.2 | 0.93  (0.6-1.43) | 0.74 |
|  | PostLD | 94.2 | 146 | 155 | 3.8 | 1.73  (0.85-.52) | 0.13 |
| North Ayrshire | 2019 | 85.8 | 952 | 1109 | NA | NA | NA |
|  | PreLD | 82.7 | 221 | 257 | -3.1 | 1.01  (0.69-1.5) | 0.95 |
|  | LD | 88.7 | 354 | 399 | 2.9 | 1.3  (0.91-1.85) | 0.15 |
|  | PostLD | 85.4 | 171 | 201 | -0.4 | 0.94  (0.62-1.44) | 0.77 |
| North Lanarkshire | 2019 | 87.2 | 3090 | 3542 | NA | NA | NA |
|  | PreLD | 85.6 | 623 | 724 | -1.6 | 0.9  (0.72-1.14) | 0.39 |
|  | LD | 91.3 | 1037 | 1136 | 4.1 | 1.53  (1.22-1.93) | <0.001 |
|  | PostLD | 89.3 | 508 | 566 | 2.1 | 1.28  (0.96-1.71) | 0.09 |
| Orkney Islands | 2019 | 85 | 175 | 206 | NA | NA | NA |
|  | PreLD | 93.2 | 29 | 31 | 8.2 | 2.57  (0.58-11.32) | 0.21 |
|  | LD | 87 | 53 | 61 | 2 | 1.88  (1.39-2.53) | <0.001 |
|  | PostLD | 97 | 26 | 27 | 12 | 1.48  (1.02-2.14) | 0.04 |
| Perth and Kinross | 2019 | 81.8 | 1016 | 1242 | NA | NA | NA |
|  | PreLD | 76.9 | 211 | 264 | -4.9 | 0.89  (0.63-1.24) | 0.48 |
|  | LD | 85.3 | 382 | 446 | 3.5 | 1.33  (0.98-1.79) | 0.07 |
|  | PostLD | 85.5 | 171 | 200 | 3.7 | 1.31  (0.86-1.99) | 0.2 |
| Renfrewshire | 2019 | 88.2 | 1508 | 1710 | NA | NA | NA |
|  | PreLD | 90.8 | 334 | 366 | 2.6 | 1.4  (0.95-2.07) | 0.09 |
|  | LD | 95 | 593 | 623 | 6.8 | 2.65  (1.78-3.93) | <0.001 |
|  | PostLD | 92.2 | 246 | 269 | 4 | 1.43  (0.91-2.25) | 0.12 |
| Scottish Borders | 2019 | 85.6 | 740 | 864 | NA | NA | NA |
|  | PreLD | 83.3 | 178 | 212 | -2.3 | 0.88  (0.58-1.33) | 0.53 |
|  | LD | 89.6 | 283 | 315 | 4 | 1.48  (0.98-2.24) | 0.06 |
|  | PostLD | 86.8 | 134 | 153 | 1.2 | 1.18  (0.7-1.98) | 0.53 |
| Shetland Islands | 2019 | 89.5 | 197 | 220 | NA | NA | NA |
|  | PreLD | 83.8 | 21 | 25 | -5.8 | 0.61  (0.19-1.94) | 0.41 |
|  | LD | 82.8 | 56 | 68 | -6.7 | 1.88  (1.39-2.53) | <0.001 |
|  | PostLD | 77.3 | 25 | 32 | -12.2 | 1.48  (1.02-2.14) | 0.04 |
| South Ayrshire | 2019 | 92.4 | 830 | 898 | NA | NA | NA |
|  | PreLD | 89.5 | 165 | 184 | -2.9 | 0.71  (0.42-1.22) | 0.21 |
|  | LD | 95.5 | 277 | 290 | 3.1 | 1.75  (0.95-3.21) | 0.07 |
|  | PostLD | 92.3 | 121 | 132 | -0.1 | 0.9  (0.46-1.75) | 0.76 |
| South Lanarkshire | 2019 | 88.2 | 2821 | 3199 | NA | NA | NA |
|  | PreLD | 85.9 | 590 | 666 | -2.3 | 1.04  (0.8-1.35) | 0.77 |
|  | LD | 91.8 | 1014 | 1104 | 3.6 | 1.51  (1.19-1.92) | <0.001 |
|  | PostLD | 90.8 | 489 | 539 | 2.6 | 1.31  (0.96-1.79) | 0.09 |
| West Dunbartonshire | 2019 | 80.2 | 693 | 864 | NA | NA | NA |
|  | PreLD | 85.5 | 131 | 160 | 5.3 | 1.11  (0.72-1.72) | 0.63 |
|  | LD | 88.7 | 263 | 296 | 8.5 | 1.97  (1.32-2.93) | <0.001 |
|  | PostLD | 85 | 128 | 149 | 4.8 | 1.5  (0.92-2.46) | 0.1 |
| West Lothian | 2019 | 82.7 | 1525 | 1845 | NA | NA | NA |
|  | PreLD | 85.3 | 318 | 368 | 2.6 | 1.33  (0.97-1.84) | 0.08 |
|  | LD | 89.6 | 510 | 567 | 6.9 | 1.88  (1.39-2.53) | <0.001 |
|  | PostLD | 87.5 | 254 | 290 | 4.8 | 1.48  (1.02-2.14) | 0.04 |
| Western Isles | 2019 | 89.2 | 173 | 194 | NA | NA | NA |
|  | PreLD | 83.3 | 30 | 36 | -5.9 | 0.61  (0.23-1.63) | 0.32 |
|  | LD | 93.5 | 60 | 64 | 4.3 | 0.87  (0.69-1.08) | 0.2 |
|  | PostLD | 88.9 | 24 | 28 | -0.3 | 0.83  (0.62-1.12) | 0.23 |

**C Third dose 6in1**

| **HSCP** | **Time period** | **% uptake (within 4 weeks)** | **Number received** | **Number eligible** | **% point change from 2019** | **OR compared to 2019**  **(95% CI)** | **p value** |
| --- | --- | --- | --- | --- | --- | --- | --- |
| Aberdeen City | 2019 | 72.4 | 1609 | 2222 | NA | NA | NA |
|  | PreLD | 65.8 | 345 | 520 | -6.6 | 0.75  (0.61-0.92) | 0.01 |
|  | LD | 62.3 | 450 | 719 | -10.1 | 0.64  (0.53-0.76) | <0.001 |
|  | PostLD | 75 | 252 | 336 | 2.6 | 1.14  (0.88-1.49) | 0.32 |
| Aberdeenshire | 2019 | 86.8 | 2266 | 2611 | NA | NA | NA |
|  | PreLD | 82 | 446 | 536 | -4.8 | 0.75  (0.59-0.97) | 0.03 |
|  | LD | 86.1 | 721 | 838 | -0.7 | 0.94  (0.75-1.18) | 0.58 |
|  | PostLD | 80.8 | 336 | 416 | -6 | 0.64  (0.49-0.84) | <0.001 |
| Angus | 2019 | 71.2 | 738 | 1036 | NA | NA | NA |
|  | PreLD | 76 | 159 | 214 | 4.8 | 1.17  (0.84-1.63) | 0.37 |
|  | LD | 75.9 | 261 | 345 | 4.7 | 1.25  (0.95-1.66) | 0.11 |
|  | PostLD | 79 | 121 | 154 | 7.8 | 1.48  (0.98-2.23) | 0.06 |
| Argyll and Bute | 2019 | 69.3 | 443 | 639 | NA | NA | NA |
|  | PreLD | 72.9 | 120 | 170 | 3.6 | 1.06  (0.73-1.54) | 0.75 |
|  | LD | 78.2 | 178 | 230 | 8.9 | 1.51  (1.07-2.15) | 0.02 |
|  | PostLD | 78.8 | 94 | 118 | 9.5 | 1.73  (1.07-2.8) | 0.02 |
| Clackmannanshire and Stirling | 2019 | 72.3 | 860 | 1189 | NA | NA | NA |
|  | PreLD | 74.4 | 217 | 282 | 2.1 | 1.28  (0.94-1.73) | 0.12 |
|  | LD | 89 | 384 | 432 | 16.7 | 3.06  (2.21-4.24) | <0.001 |
|  | PostLD | 86.1 | 167 | 193 | 13.8 | 2.46  (1.59-3.79) | <0.001 |
| Dumfries and Galloway | 2019 | 68.5 | 805 | 1175 | NA | NA | NA |
|  | PreLD | 66.4 | 187 | 280 | -2.1 | 0.92  (0.7-1.22) | 0.58 |
|  | LD | 85.5 | 339 | 401 | 17 | 2.51  (1.87-3.38) | <0.001 |
|  | PostLD | 81 | 177 | 221 | 12.5 | 1.85  (1.3-2.63) | <0.001 |
| Dundee City | 2019 | 65.9 | 933 | 1416 | NA | NA | NA |
|  | PreLD | 62 | 213 | 325 | -3.9 | 0.98  (0.76-1.27) | 0.9 |
|  | LD | 73.2 | 326 | 442 | 7.3 | 1.45  (1.15-1.85) | <0.001 |
|  | PostLD | 72.5 | 170 | 235 | 6.6 | 1.35  (1-1.84) | 0.05 |
| East Ayrshire | 2019 | 78.5 | 930 | 1184 | NA | NA | NA |
|  | PreLD | 78.4 | 204 | 260 | -0.1 | 0.99  (0.72-1.38) | 0.98 |
|  | LD | 84.9 | 357 | 420 | 6.4 | 1.55  (1.14-2.09) | <0.001 |
|  | PostLD | 87 | 172 | 198 | 8.5 | 1.81  (1.17-2.79) | 0.01 |
| East Dunbartonshire | 2019 | 79.3 | 808 | 1019 | NA | NA | NA |
|  | PreLD | 86.3 | 180 | 213 | 7 | 1.42  (0.95-2.13) | 0.08 |
|  | LD | 93.2 | 304 | 326 | 13.9 | 3.61  (2.28-5.71) | <0.001 |
|  | PostLD | 92.4 | 152 | 165 | 13.1 | 3.05  (1.7-5.49) | <0.001 |
| East Lothian | 2019 | 70.2 | 786 | 1119 | NA | NA | NA |
|  | PreLD | 67.9 | 153 | 225 | -2.3 | 0.9  (0.66-1.23) | 0.5 |
|  | LD | 86.9 | 298 | 345 | 16.7 | 2.69  (1.92-3.75) | <0.001 |
|  | PostLD | 82.9 | 140 | 167 | 12.7 | 2.2  (1.43-3.38) | <0.001 |
| East Renfrewshire | 2019 | 85.3 | 795 | 932 | NA | NA | NA |
|  | PreLD | 84.5 | 145 | 167 | -0.8 | 1.14  (0.7-1.84) | 0.61 |
|  | LD | 95.6 | 294 | 310 | 10.3 | 3.17  (1.85-5.41) | <0.001 |
|  | PostLD | 89.6 | 134 | 147 | 4.3 | 1.78  (0.98-3.23) | 0.06 |
| Edinburgh | 2019 | 68 | 3058 | 4494 | NA | NA | NA |
|  | PreLD | 68.1 | 721 | 1043 | 0.1 | 1.05  (0.91-1.22) | 0.5 |
|  | LD | 80.7 | 1253 | 1545 | 12.7 | 2.02  (1.75-2.32) | <0.001 |
|  | PostLD | 75.8 | 583 | 771 | 7.8 | 1.46  (1.22-1.74) | <0.001 |
| Falkirk | 2019 | 65.5 | 969 | 1480 | NA | NA | NA |
|  | PreLD | 73.7 | 241 | 337 | 8.2 | 1.32  (1.02-1.72) | 0.03 |
|  | LD | 82 | 412 | 503 | 16.5 | 2.39  (1.86-3.07) | <0.001 |
|  | PostLD | 83.3 | 212 | 254 | 17.8 | 2.66  (1.88-3.77) | <0.001 |
| Fife | 2019 | 75 | 2571 | 3426 | NA | NA | NA |
|  | PreLD | 73.5 | 556 | 768 | -1.5 | 0.87  (0.73-1.04) | 0.13 |
|  | LD | 81.7 | 940 | 1151 | 6.7 | 1.48  (1.25-1.75) | <0.001 |
|  | PostLD | 86.4 | 484 | 561 | 11.4 | 2.09  (1.62-2.69) | <0.001 |
| Glasgow City | 2019 | 68.7 | 4454 | 6484 | NA | NA | NA |
|  | PreLD | 72.9 | 1011 | 1424 | 4.2 | 1.12  (0.98-1.27) | 0.09 |
|  | LD | 83.9 | 1796 | 2140 | 15.2 | 2.38  (2.1-2.7) | <0.001 |
|  | PostLD | 84.4 | 842 | 998 | 15.7 | 2.46  (2.06-2.94) | <0.001 |
| Highland | 2019 | 67.5 | 1340 | 1984 | NA | NA | NA |
|  | PreLD | 61.4 | 279 | 461 | -6.1 | 0.74  (0.6-0.91) | <0.001 |
|  | LD | 73.1 | 480 | 653 | 5.6 | 1.33  (1.09-1.62) | <0.001 |
|  | PostLD | 63.9 | 199 | 311 | -3.6 | 0.85  (0.67-1.1) | 0.22 |
| Inverclyde | 2019 | 84.8 | 542 | 639 | NA | NA | NA |
|  | PreLD | 78.6 | 121 | 151 | -6.2 | 0.72  (0.46-1.14) | 0.16 |
|  | LD | 87.4 | 206 | 238 | 2.6 | 1.15  (0.75-1.77) | 0.52 |
|  | PostLD | 84.6 | 91 | 105 | -0.2 | 1.16  (0.64-2.13) | 0.62 |
| Midlothian | 2019 | 69 | 791 | 1146 | NA | NA | NA |
|  | PreLD | 66.6 | 151 | 222 | -2.4 | 0.95  (0.7-1.3) | 0.77 |
|  | LD | 79.7 | 288 | 360 | 10.7 | 1.8  (1.35-2.39) | <0.001 |
|  | PostLD | 82.4 | 143 | 173 | 13.4 | 2.14  (1.42-3.23) | <0.001 |
| Moray | 2019 | 80.5 | 680 | 845 | NA | NA | NA |
|  | PreLD | 66.2 | 129 | 188 | -14.3 | 0.53  (0.37-0.75) | <0.001 |
|  | LD | 80.9 | 259 | 320 | 0.4 | 1.03  (0.74-1.43) | 0.86 |
|  | PostLD | 86.8 | 134 | 155 | 6.3 | 1.55  (0.95-2.53) | 0.08 |
| North Ayrshire | 2019 | 71.1 | 781 | 1099 | NA | NA | NA |
|  | PreLD | 74.3 | 198 | 268 | 3.2 | 1.15  (0.85-1.56) | 0.36 |
|  | LD | 80.2 | 334 | 417 | 9.1 | 1.55  (0.95-2.53) | 0.08 |
|  | PostLD | 67 | 117 | 175 | -4.1 | 0.82  (0.58-1.15) | 0.26 |
| North Lanarkshire | 2019 | 74.5 | 2651 | 3558 | NA | NA | NA |
|  | PreLD | 77.7 | 579 | 758 | 3.2 | 1.11  (0.92-1.33) | 0.28 |
|  | LD | 83.6 | 959 | 1148 | 9.1 | 1.74  (1.46-2.06) | <0.001 |
|  | PostLD | 77.8 | 404 | 517 | 3.3 | 1.22  (0.98-1.53) | 0.07 |
| Orkney Islands | 2019 | 76 | 155 | 204 | NA | NA | NA |
|  | PreLD | 82.2 | 27 | 33 | 6.2 | 1.42  (0.56-3.65) | 0.46 |
|  | LD | 79.2 | 39 | 51 | 3.2 | 2.22  (1.76-2.79) | <0.001 |
|  | PostLD | 94.4 | 28 | 29 | 18.5 | 1.74  (1.27-2.39) | <0.001 |
| Perth and Kinross | 2019 | 69.8 | 847 | 1213 | NA | NA | NA |
|  | PreLD | 57.3 | 204 | 313 | -12.5 | 0.81  (0.62-1.05) | 0.11 |
|  | LD | 75.7 | 317 | 415 | 5.9 | 1.4  (1.08-1.81) | 0.01 |
|  | PostLD | 74.8 | 166 | 219 | 5 | 1.35  (0.97-1.89) | 0.07 |
| Renfrewshire | 2019 | 78.4 | 1342 | 1712 | NA | NA | NA |
|  | PreLD | 81.3 | 307 | 386 | 2.9 | 1.07  (0.82-1.41) | 0.62 |
|  | LD | 85.1 | 537 | 630 | 6.7 | 1.59  (1.24-2.04) | <0.001 |
|  | PostLD | 84.5 | 228 | 271 | 6.1 | 1.46  (1.03-2.07) | 0.03 |
| Scottish Borders | 2019 | 76.4 | 662 | 867 | NA | NA | NA |
|  | PreLD | 68.3 | 165 | 239 | -8.1 | 0.69  (0.5-0.95) | 0.02 |
|  | LD | 83.3 | 250 | 303 | 6.9 | 1.46  (1.04-2.04) | 0.03 |
|  | PostLD | 73.5 | 103 | 137 | -2.9 | 0.94  (0.62-1.43) | 0.76 |
| Shetland Islands | 2019 | 77.2 | 169 | 219 | NA | NA | NA |
|  | PreLD | 87 | 27 | 31 | 9.8 | 2  (0.67-5.98) | 0.22 |
|  | LD | 85.2 | 56 | 65 | 8 | 2.22  (1.76-2.79) | <0.001 |
|  | PostLD | 42.8 | 13 | 31 | -34.5 | 1.74  (1.27-2.39) | <0.001 |
| South Ayrshire | 2019 | 82.1 | 760 | 926 | NA | NA | NA |
|  | PreLD | 79.6 | 149 | 180 | -2.5 | 1.05  (0.69-1.6) | 0.82 |
|  | LD | 90.5 | 271 | 301 | 8.4 | 1.97  (1.31-2.98) | <0.001 |
|  | PostLD | 85 | 103 | 122 | 2.9 | 1.18  (0.71-1.99) | 0.52 |
| South Lanarkshire | 2019 | 76.6 | 2469 | 3225 | NA | NA | NA |
|  | PreLD | 75 | 587 | 745 | -1.6 | 1.14  (0.94-1.38) | 0.19 |
|  | LD | 85.1 | 893 | 1043 | 8.5 | 1.82  (1.51-2.21) | <0.001 |
|  | PostLD | 80.6 | 438 | 545 | 4 | 1.25  (1-1.57) | 0.05 |
| West Dunbartonshire | 2019 | 67.6 | 585 | 866 | NA | NA | NA |
|  | PreLD | 75.7 | 129 | 190 | 8.1 | 1.02  (0.73-1.42) | 0.93 |
|  | LD | 84.5 | 238 | 281 | 16.9 | 2.66  (1.86-3.79) | <0.001 |
|  | PostLD | 78.8 | 116 | 147 | 11.2 | 1.8  (1.18-2.74) | 0.01 |
| West Lothian | 2019 | 67.2 | 1244 | 1850 | NA | NA | NA |
|  | PreLD | 73.7 | 266 | 382 | 6.5 | 1.12  (0.88-1.42) | 0.36 |
|  | LD | 82.1 | 487 | 594 | 14.9 | 2.22  (1.76-2.79) | <0.001 |
|  | PostLD | 77.6 | 193 | 247 | 10.4 | 1.74  (1.27-2.39) | <0.001 |
| Western Isles | 2019 | 74.1 | 149 | 201 | NA | NA | NA |
|  | PreLD | 60.8 | 18 | 30 | -13.3 | 0.52  (0.24-1.16) | 0.11 |
|  | LD | 91.6 | 62 | 69 | 17.5 | 0.64  (0.53-0.76) | <0.001 |
|  | PostLD | 76.7 | 23 | 30 | 2.6 | 1.14  (0.88-1.49) | 0.32 |

**D First dose MMR**

| **HSCP** | **Time period** | **% uptake (within 4 weeks)** | **Number received** | **Number eligible** | **% point change from 2019** | **OR compared to 2019**  **(94% CI)** | **p value** |
| --- | --- | --- | --- | --- | --- | --- | --- |
| Aberdeen City | 2019 | 55.1 | 1235 | 2243 | NA | NA | NA |
|  | PreLD | 53.4 | 259 | 501 | -1.7 | 0.87  (0.72-1.06) | 0.17 |
|  | LD | 69.3 | 553 | 795 | 14.2 | 1.87  (1.57-2.22) | <0.001 |
|  | PostLD | 71.5 | 285 | 399 | 16.4 | 2.04  (1.62-2.57) | <0.001 |
| Aberdeenshire | 2019 | 49.4 | 1383 | 2798 | NA | NA | NA |
|  | PreLD | 48 | 254 | 554 | -1.4 | 0.87  (0.72-1.04) | 0.12 |
|  | LD | 56.8 | 536 | 936 | 7.4 | 1.37  (1.18-1.59) | <0.001 |
|  | PostLD | 48.9 | 221 | 448 | -0.5 | 1  (0.82-1.22) | 0.97 |
| Angus | 2019 | 69.3 | 681 | 982 | NA | NA | NA |
|  | PreLD | 72.1 | 182 | 250 | 2.8 | 1.18  (0.87-1.61) | 0.29 |
|  | LD | 85.6 | 311 | 365 | 16.3 | 2.55  (1.85-3.5) | <0.001 |
|  | PostLD | 80 | 129 | 162 | 10.7 | 1.73  (1.15-2.59) | 0.01 |
| Argyll and Bute | 2019 | 64.8 | 440 | 679 | NA | NA | NA |
|  | PreLD | 74 | 112 | 157 | 9.2 | 1.35  (0.92-1.98) | 0.12 |
|  | LD | 78.6 | 178 | 226 | 13.8 | 2.01  (1.41-2.87) | <0.001 |
|  | PostLD | 72.9 | 74 | 102 | 8.1 | 1.44  (0.9-2.28) | 0.13 |
| Clackmannanshire and Stirling | 2019 | 70.8 | 902 | 1274 | NA | NA | NA |
|  | PreLD | 73.4 | 179 | 242 | 2.6 | 1.17  (0.86-1.6) | 0.32 |
|  | LD | 83.5 | 377 | 449 | 12.7 | 2.16  (1.63-2.86) | <0.001 |
|  | PostLD | 86.8 | 178 | 205 | 16 | 2.72  (1.78-4.15) | <0.001 |
| Dumfries and Galloway | 2019 | 72 | 907 | 1259 | NA | NA | NA |
|  | PreLD | 71.7 | 198 | 272 | -0.3 | 1.04  (0.77-1.39) | 0.8 |
|  | LD | 84 | 338 | 401 | 12 | 2.08  (1.55-2.8) | <0.001 |
|  | PostLD | 79.7 | 178 | 224 | 7.7 | 1.5  (1.06-2.12) | 0.02 |
| Dundee City | 2019 | 60.5 | 857 | 1416 | NA | NA | NA |
|  | PreLD | 60.3 | 188 | 293 | -0.2 | 1.17  (0.9-1.52) | 0.24 |
|  | LD | 76 | 388 | 510 | 15.5 | 2.07  (1.65-2.61) | <0.001 |
|  | PostLD | 74.5 | 198 | 265 | 14 | 1.93  (1.43-2.59) | <0.001 |
| East Ayrshire | 2019 | 58.9 | 703 | 1194 | NA | NA | NA |
|  | PreLD | 56.3 | 166 | 260 | -2.6 | 1.23  (0.93-1.63) | 0.14 |
|  | LD | 69 | 306 | 441 | 10.1 | 1.58  (1.25-2) | <0.001 |
|  | PostLD | 70.4 | 154 | 218 | 11.5 | 1.68  (1.23-2.3) | <0.001 |
| East Dunbartonshire | 2019 | 75.8 | 803 | 1060 | NA | NA | NA |
|  | PreLD | 79.3 | 188 | 240 | 3.5 | 1.16  (0.83-1.62) | 0.4 |
|  | LD | 91.1 | 303 | 335 | 15.3 | 3.03  (2.05-4.48) | <0.001 |
|  | PostLD | 86.8 | 162 | 187 | 11 | 2.07  (1.33-3.23) | <0.001 |
| East Lothian | 2019 | 71.2 | 800 | 1124 | NA | NA | NA |
|  | PreLD | 70.7 | 166 | 233 | -0.5 | 1  (0.73-1.37) | 0.98 |
|  | LD | 87.3 | 352 | 405 | 16.1 | 2.69  (1.96-3.69) | <0.001 |
|  | PostLD | 80.2 | 157 | 198 | 9 | 1.55  (1.07-2.24) | 0.02 |
| East Renfrewshire | 2019 | 74.6 | 745 | 998 | NA | NA | NA |
|  | PreLD | 76.6 | 157 | 199 | 2 | 1.27  (0.88-1.84) | 0.21 |
|  | LD | 84.8 | 280 | 327 | 10.2 | 2.02  (1.44-2.84) | <0.001 |
|  | PostLD | 85.2 | 136 | 159 | 10.6 | 2.01  (1.26-3.19) | <0.001 |
| Edinburgh | 2019 | 68.1 | 3140 | 4611 | NA | NA | NA |
|  | PreLD | 68.1 | 709 | 1037 | 0 | 1.01  (0.88-1.17) | 0.86 |
|  | LD | 80.1 | 1315 | 1642 | 12 | 1.88  (1.64-2.16) | <0.001 |
|  | PostLD | 73.1 | 562 | 766 | 5 | 1.29  (1.09-1.53) | <0.001 |
| Falkirk | 2019 | 68.8 | 1052 | 1530 | NA | NA | NA |
|  | PreLD | 67.8 | 245 | 343 | -1 | 1.14  (0.88-1.47) | 0.33 |
|  | LD | 85.4 | 450 | 530 | 16.6 | 2.56  (1.97-3.32) | <0.001 |
|  | PostLD | 80.8 | 216 | 265 | 12 | 2  (1.44-2.78) | <0.001 |
| Fife | 2019 | 67 | 2379 | 3553 | NA | NA | NA |
|  | PreLD | 70.9 | 535 | 762 | 3.9 | 1.16  (0.98-1.38) | 0.08 |
|  | LD | 70.5 | 856 | 1214 | 3.5 | 1.18  (1.02-1.36) | 0.02 |
|  | PostLD | 68 | 414 | 608 | 1 | 1.05  (0.88-1.27) | 0.58 |
| Glasgow City | 2019 | 67 | 4209 | 6281 | NA | NA | NA |
|  | PreLD | 76.4 | 1079 | 1413 | 9.4 | 1.59  (1.39-1.82) | <0.001 |
|  | LD | 80.6 | 1942 | 2404 | 13.6 | 2.07  (1.85-2.32) | <0.001 |
|  | PostLD | 80.4 | 921 | 1146 | 13.4 | 2.02  (1.73-2.35) | <0.001 |
| Highland | 2019 | 56.7 | 1156 | 2039 | NA | NA | NA |
|  | PreLD | 55.1 | 254 | 434 | -1.6 | 1.08  (0.87-1.33) | 0.48 |
|  | LD | 68.5 | 511 | 746 | 11.8 | 1.66  (1.39-1.98) | <0.001 |
|  | PostLD | 58 | 216 | 371 | 1.3 | 1.06  (0.85-1.33) | 0.58 |
| Inverclyde | 2019 | 75.7 | 535 | 707 | NA | NA | NA |
|  | PreLD | 62.6 | 100 | 151 | -13.1 | 0.63  (0.43-0.92) | 0.02 |
|  | LD | 82 | 161 | 195 | 6.3 | 1.52  (1.01-2.29) | 0.04 |
|  | PostLD | 82.1 | 87 | 106 | 6.4 | 1.47  (0.87-2.49) | 0.15 |
| Midlothian | 2019 | 65.3 | 733 | 1122 | NA | NA | NA |
|  | PreLD | 64.8 | 177 | 258 | -0.5 | 1.16  (0.87-1.55) | 0.32 |
|  | LD | 82.8 | 349 | 421 | 17.5 | 2.57  (1.94-3.41) | <0.001 |
|  | PostLD | 83.5 | 163 | 196 | 18.2 | 2.62  (1.77-3.89) | <0.001 |
| Moray | 2019 | 54.1 | 447 | 827 | NA | NA | NA |
|  | PreLD | 62.7 | 111 | 181 | 8.6 | 1.35  (0.97-1.87) | 0.08 |
|  | LD | 86.4 | 264 | 308 | 32.3 | 5.1  (3.6-7.22) | <0.001 |
|  | PostLD | 86.3 | 130 | 150 | 32.2 | 5.53  (3.38-9.02) | <0.001 |
| North Ayrshire | 2019 | 56.5 | 648 | 1146 | NA | NA | NA |
|  | PreLD | 58.6 | 144 | 253 | 2.1 | 1.02  (0.77-1.34) | 0.91 |
|  | LD | 73 | 272 | 373 | 16.5 | 2.07  (1.6-2.67) | <0.001 |
|  | PostLD | 61.2 | 144 | 243 | 4.7 | 1.12  (0.84-1.48) | 0.44 |
| North Lanarkshire | 2019 | 69.8 | 2448 | 3509 | NA | NA | NA |
|  | PreLD | 68.3 | 566 | 791 | -1.5 | 1.09  (0.92-1.29) | 0.32 |
|  | LD | 83.4 | 1078 | 1286 | 13.6 | 2.25  (1.9-2.65) | <0.001 |
|  | PostLD | 78.4 | 488 | 624 | 8.6 | 1.56  (1.27-1.91) | <0.001 |
| Orkney Islands | 2019 | 53.9 | 111 | 206 | NA | NA | NA |
|  | PreLD | 55.7 | 19 | 33 | 1.8 | 1.16  (0.55-2.44) | 0.69 |
|  | LD | 73.2 | 50 | 67 | 19.3 | 2.8  (2.23-3.53) | <0.001 |
|  | PostLD | 71.6 | 29 | 41 | 17.7 | 1.89  (1.42-2.51) | <0.001 |
| Perth and Kinross | 2019 | 66.4 | 855 | 1287 | NA | NA | NA |
|  | PreLD | 65.6 | 177 | 263 | -0.8 | 1.04  (0.78-1.38) | 0.79 |
|  | LD | 78.2 | 351 | 448 | 11.8 | 1.83  (1.42-2.35) | <0.001 |
|  | PostLD | 72.7 | 156 | 217 | 6.3 | 1.29  (0.94-1.78) | 0.11 |
| Renfrewshire | 2019 | 69.1 | 1210 | 1750 | NA | NA | NA |
|  | PreLD | 73.2 | 301 | 400 | 4.1 | 1.36  (1.06-1.74) | 0.02 |
|  | LD | 83.5 | 507 | 607 | 14.4 | 2.26  (1.78-2.87) | <0.001 |
|  | PostLD | 74.9 | 216 | 287 | 5.8 | 1.36  (1.02-1.81) | 0.04 |
| Scottish Borders | 2019 | 67.1 | 658 | 981 | NA | NA | NA |
|  | PreLD | 66.9 | 134 | 204 | -0.2 | 0.94  (0.68-1.29) | 0.7 |
|  | LD | 78.7 | 250 | 317 | 11.6 | 1.83  (1.36-2.47) | <0.001 |
|  | PostLD | 70.8 | 114 | 154 | 3.7 | 1.4  (0.95-2.05) | 0.09 |
| Shetland Islands | 2019 | 29.8 | 68 | 228 | NA | NA | NA |
|  | PreLD | 54.1 | 19 | 35 | 24.3 | 2.79  (1.36-5.76) | 0.01 |
|  | LD | 39.5 | 27 | 69 | 9.7 | 2.8  (2.23-3.53) | <0.001 |
|  | PostLD | 24.5 | 12 | 41 | -5.3 | 1.89  (1.42-2.51) | <0.001 |
| South Ayrshire | 2019 | 61.5 | 583 | 948 | NA | NA | NA |
|  | PreLD | 70.8 | 128 | 182 | 9.3 | 1.48  (1.05-2.09) | 0.02 |
|  | LD | 81.4 | 274 | 333 | 19.9 | 2.91  (2.13-3.97) | <0.001 |
|  | PostLD | 78.6 | 132 | 169 | 17.1 | 2.23  (1.52-3.29) | <0.001 |
| South Lanarkshire | 2019 | 70.1 | 2271 | 3241 | NA | NA | NA |
|  | PreLD | 71.3 | 548 | 731 | 1.2 | 1.28  (1.06-1.54) | 0.01 |
|  | LD | 81.8 | 960 | 1171 | 11.7 | 1.94  (1.64-2.3) | <0.001 |
|  | PostLD | 81 | 460 | 567 | 10.9 | 1.84  (1.47-2.3) | <0.001 |
| West Dunbartonshire | 2019 | 65.8 | 573 | 871 | NA | NA | NA |
|  | PreLD | 75.6 | 136 | 185 | 9.8 | 1.44  (1.01-2.06) | 0.04 |
|  | LD | 79.4 | 249 | 311 | 13.6 | 2.09  (1.53-2.85) | <0.001 |
|  | PostLD | 78 | 128 | 164 | 12.2 | 1.85  (1.25-2.75) | <0.001 |
| West Lothian | 2019 | 66.7 | 1238 | 1857 | NA | NA | NA |
|  | PreLD | 70.1 | 301 | 418 | 3.4 | 1.29  (1.02-1.63) | 0.04 |
|  | LD | 84.6 | 583 | 687 | 17.9 | 2.8  (2.23-3.53) | <0.001 |
|  | PostLD | 79.1 | 257 | 325 | 12.4 | 1.89  (1.42-2.51) | <0.001 |
| Western Isles | 2019 | 52.6 | 113 | 215 | NA | NA | NA |
|  | PreLD | 64.6 | 24 | 38 | 12 | 1.55  (0.76-3.15) | 0.23 |
|  | LD | 77.1 | 55 | 72 | 24.5 | 1.87  (1.57-.22) | <0.001 |
|  | PostLD | 63.2 | 23 | 35 | 10.6 | 2.04  (1.62-2.57) | <0.001 |

**E Second dose MMR**

| **HSCP** | **Time period** | **% uptake (within 4 weeks)** | **Number received** | **Number eligible** | **% point change from 2019** | **OR compared to 2019**  **(95% CI)** | **p value** |
| --- | --- | --- | --- | --- | --- | --- | --- |
| Angus | 2019 | 43.8 | 498 | 1137 | NA | NA | NA |
|  | PreLD | 54.7 | 147 | 275 | 10.9 | 1.47  (1.13-1.92) | <0.001 |
|  | LD | 74.1 | 266 | 367 | 30.3 | 3.38  (2.61-4.37) | <0.001 |
|  | PostLD | 62.7 | 118 | 189 | 18.9 | 2.13  (1.55-2.93) | <0.001 |
| Argyll and Bute | 2019 | 57.3 | 436 | 761 | NA | NA | NA |
|  | PreLD | 55.8 | 92 | 166 | -1.5 | 0.93  (0.66-1.3) | 0.66 |
|  | LD | 61.2 | 169 | 279 | 3.9 | 1.15  (0.87-1.51) | 0.34 |
|  | PostLD | 60.8 | 65 | 108 | 3.5 | 1.13  (0.75-1.7) | 0.57 |
| Clackmannanshire and Stirling | 2019 | 52.3 | 770 | 1471 | NA | NA | NA |
|  | PreLD | 54.7 | 193 | 320 | 2.4 | 1.38  (1.08-1.77) | 0.01 |
|  | LD | 68.3 | 341 | 504 | 16 | 1.9  (1.54-2.36) | <0.001 |
|  | PostLD | 60.5 | 144 | 237 | 8.2 | 1.41  (1.07-1.87) | 0.02 |
| Dumfries and Galloway | 2019 | 63.2 | 871 | 1379 | NA | NA | NA |
|  | PreLD | 60.6 | 199 | 314 | -2.6 | 1.01  (0.78-1.3) | 0.94 |
|  | LD | 71 | 308 | 437 | 7.8 | 1.39  (1.1-1.76) | 0.01 |
|  | PostLD | 72.5 | 187 | 257 | 9.3 | 1.56  (1.16-2.09) | <0.001 |
| Dundee City | 2019 | 32.9 | 485 | 1476 | NA | NA | NA |
|  | PreLD | 39.4 | 143 | 346 | 6.5 | 1.44  (1.13-1.83) | <0.001 |
|  | LD | 58.9 | 309 | 523 | 26 | 2.95  (2.4-3.62) | <0.001 |
|  | PostLD | 52.8 | 137 | 256 | 19.9 | 2.35  (1.8-3.08) | <0.001 |
| East Ayrshire | 2019 | 39.7 | 522 | 1316 | NA | NA | NA |
|  | PreLD | 26.6 | 100 | 304 | -13.1 | 0.75  (0.57-0.97) | 0.03 |
|  | LD | 43.8 | 192 | 445 | 4.1 | 1.15  (0.93-1.44) | 0.2 |
|  | PostLD | 43.5 | 86 | 198 | 3.8 | 1.17  (0.86-1.58) | 0.31 |
| East Dunbartonshire | 2019 | 56.4 | 674 | 1196 | NA | NA | NA |
|  | PreLD | 63.6 | 197 | 300 | 7.2 | 1.48  (1.14-1.93) | <0.001 |
|  | LD | 82.6 | 370 | 447 | 26.2 | 3.72  (2.84-4.88) | <0.001 |
|  | PostLD | 85.1 | 188 | 223 | 28.7 | 4.16  (2.85-6.07) | <0.001 |
| East Lothian | 2019 | 60 | 762 | 1271 | NA | NA | NA |
|  | PreLD | 56.2 | 161 | 271 | -3.8 | 0.98  (0.75-1.28) | 0.87 |
|  | LD | 78.5 | 315 | 401 | 18.5 | 2.45  (1.88-3.18) | <0.001 |
|  | PostLD | 59.1 | 112 | 184 | -0.9 | 1.04  (0.76-1.43) | 0.81 |
| East Renfrewshire | 2019 | 62.5 | 731 | 1169 | NA | NA | NA |
|  | PreLD | 63.3 | 176 | 258 | 0.8 | 1.29  (0.96-1.71) | 0.09 |
|  | LD | 83.5 | 309 | 369 | 21 | 3.09  (2.28-4.17) | <0.001 |
|  | PostLD | 84.1 | 156 | 186 | 21.6 | 3.12  (2.07-4.69) | <0.001 |
| Edinburgh | 2019 | 56.3 | 2727 | 4846 | NA | NA | NA |
|  | PreLD | 60.5 | 660 | 1117 | 4.2 | 1.12  (0.98-1.28) | 0.09 |
|  | LD | 65.6 | 1103 | 1679 | 9.3 | 1.49  (1.33-1.67) | <0.001 |
|  | PostLD | 61.3 | 492 | 806 | 5 | 1.22  (1.05-1.42) | 0.01 |
| Falkirk | 2019 | 49 | 793 | 1619 | NA | NA | NA |
|  | PreLD | 47.6 | 194 | 366 | -1.4 | 1.17  (0.94-1.47) | 0.16 |
|  | LD | 62 | 353 | 579 | 13 | 1.63  (1.34-1.97) | <0.001 |
|  | PostLD | 62.8 | 178 | 284 | 13.8 | 1.75  (1.35-2.27) | <0.001 |
| Fife | 2019 | 47 | 1799 | 3826 | NA | NA | NA |
|  | PreLD | 50.4 | 446 | 906 | 3.4 | 1.09  (0.95-1.26) | 0.23 |
|  | LD | 52.2 | 673 | 1285 | 5.2 | 1.24  (1.09-1.41) | <0.001 |
|  | PostLD | 44.7 | 257 | 573 | -2.3 | 0.92  (0.77-1.09) | 0.33 |
| Glasgow City | 2019 | 50.9 | 3262 | 6411 | NA | NA | NA |
|  | PreLD | 61.8 | 960 | 1543 | 10.9 | 1.59  (1.42-1.78) | <0.001 |
|  | LD | 70.8 | 1682 | 2378 | 19.9 | 2.33  (2.11-2.58) | <0.001 |
|  | PostLD | 72.6 | 782 | 1077 | 21.7 | 2.56  (2.22-2.95) | <0.001 |
| Highland | 2019 | 50.7 | 1144 | 2257 | NA | NA | NA |
|  | PreLD | 31.8 | 223 | 516 | -18.9 | 0.74  (0.61-0.9) | <0.001 |
|  | LD | 58.4 | 474 | 803 | 7.7 | 1.4  (1.19-1.65) | <0.001 |
|  | PostLD | 52.3 | 192 | 356 | 1.6 | 1.14  (0.91-1.43) | 0.26 |
| Inverclyde | 2019 | 61.9 | 445 | 719 | NA | NA | NA |
|  | PreLD | 63.2 | 111 | 177 | 1.3 | 1.04  (0.74-1.45) | 0.84 |
|  | LD | 71.3 | 153 | 214 | 9.4 | 1.54  (1.11-2.15) | 0.01 |
|  | PostLD | 66.4 | 91 | 128 | 4.5 | 1.51  (1-2.28) | 0.05 |
| Midlothian | 2019 | 60.2 | 736 | 1223 | NA | NA | NA |
|  | PreLD | 56.8 | 168 | 293 | -3.4 | 0.89  (0.69-1.15) | 0.37 |
|  | LD | 75.8 | 299 | 405 | 15.6 | 1.87  (1.45-2.4) | <0.001 |
|  | PostLD | 68 | 137 | 204 | 7.8 | 1.35  (0.99-1.85) | 0.06 |
| North Ayrshire | 2019 | 39.4 | 526 | 1334 | NA | NA | NA |
|  | PreLD | 35.4 | 100 | 280 | -4 | 0.85  (0.65-1.12) | 0.25 |
|  | LD | 47.7 | 205 | 427 | 8.3 | 1.42  (1.14-1.77) | <0.001 |
|  | PostLD | 42.7 | 94 | 224 | 3.3 | 1.11  (0.83-1.48) | 0.47 |
| North Lanarkshire | 2019 | 53 | 1986 | 3749 | NA | NA | NA |
|  | PreLD | 53.1 | 508 | 845 | 0.1 | 1.34  (1.15-1.56) | <0.001 |
|  | LD | 70.7 | 888 | 1252 | 17.7 | 2.17  (1.89-2.49) | <0.001 |
|  | PostLD | 66.7 | 436 | 656 | 13.7 | 1.76  (1.48-2.09) | <0.001 |
| Orkney Islands | 2019 | 37 | 71 | 192 | NA | NA | NA |
|  | PreLD | 50.6 | 16 | 33 | 13.6 | 1.6  (0.76-3.37) | 0.21 |
|  | LD | 63.6 | 43 | 66 | 26.6 | 2.46  (2.02-2.99) | <0.001 |
|  | PostLD | 77.3 | 24 | 31 | 40.3 | 1.6  (1.25-2.04) | <0.001 |
| Perth and Kinross | 2019 | 45.5 | 654 | 1436 | NA | NA | NA |
|  | PreLD | 51.5 | 190 | 356 | 6 | 1.37  (1.08-1.73) | 0.01 |
|  | LD | 63.2 | 284 | 465 | 17.7 | 1.88  (1.52-2.32) | <0.001 |
|  | PostLD | 59.1 | 121 | 203 | 13.6 | 1.76  (1.31-2.38) | <0.001 |
| Renfrewshire | 2019 | 57.5 | 1064 | 1852 | NA | NA | NA |
|  | PreLD | 60.9 | 252 | 408 | 3.4 | 1.2  (0.96-1.49) | 0.11 |
|  | LD | 70.6 | 445 | 627 | 13.1 | 1.81  (1.49-2.2) | <0.001 |
|  | PostLD | 68.8 | 215 | 310 | 11.3 | 1.68  (1.29-2.17) | <0.001 |
| Scottish Borders | 2019 | 55.5 | 616 | 1110 | NA | NA | NA |
|  | PreLD | 51.9 | 122 | 224 | -3.6 | 0.96  (0.72-1.28) | 0.78 |
|  | LD | 64.6 | 218 | 335 | 9.1 | 1.49  (1.16-1.93) | <0.001 |
|  | PostLD | 59.1 | 111 | 189 | 3.6 | 1.14  (0.83-1.56) | 0.41 |
| Shetland Islands | 2019 | 24.5 | 68 | 278 | NA | NA | NA |
|  | PreLD | 38 | 16 | 42 | 13.5 | 1.9  (0.96-3.75) | 0.06 |
|  | LD | 19 | 16 | 70 | -5.5 | 2.46  (2.02-2.99) | <0.001 |
|  | PostLD | 19.9 | 9 | 44 | -4.6 | 1.6  (1.25-2.04) | <0.001 |
| South Ayrshire | 2019 | 42.6 | 447 | 1050 | NA | NA | NA |
|  | PreLD | 37.3 | 100 | 244 | -5.3 | 0.94  (0.71-1.24) | 0.65 |
|  | LD | 54.6 | 187 | 354 | 12 | 1.51  (1.19-1.92) | <0.001 |
|  | PostLD | 56.4 | 94 | 166 | 13.8 | 1.76  (1.27-2.45) | <0.001 |
| South Lanarkshire | 2019 | 53.3 | 1839 | 3452 | NA | NA | NA |
|  | PreLD | 52 | 453 | 798 | -1.3 | 1.15  (0.99-1.35) | 0.07 |
|  | LD | 67.7 | 844 | 1247 | 14.4 | 1.84  (1.6-2.1) | <0.001 |
|  | PostLD | 64.5 | 364 | 563 | 11.2 | 1.6  (1.33-1.93) | <0.001 |
| West Dunbartonshire | 2019 | 53 | 529 | 998 | NA | NA | NA |
|  | PreLD | 62 | 125 | 200 | 9 | 1.48  (1.08-2.02) | 0.01 |
|  | LD | 68.4 | 203 | 294 | 15.4 | 1.98  (1.5-2.61) | <0.001 |
|  | PostLD | 71 | 111 | 152 | 18 | 2.4  (1.64-3.51) | <0.001 |
| West Lothian | 2019 | 59.3 | 1238 | 2089 | NA | NA | NA |
|  | PreLD | 59.3 | 291 | 493 | 0 | 0.99  (0.81-1.21) | 0.9 |
|  | LD | 79 | 576 | 737 | 19.7 | 2.46  (2.02-2.99) | <0.001 |
|  | PostLD | 69.9 | 239 | 342 | 10.6 | 1.6  (1.25-2.04) | <0.001 |
| Western Isles | 2019 | 45.6 | 104 | 228 | NA | NA | NA |
|  | PreLD | 41.4 | 20 | 48 | -4.2 | 0.85  (0.45-1.6) | 0.62 |
|  | LD | 47.8 | 43 | 89 | 2.1 | 3.38  (2.61-4.37) | <0.001 |
|  | PostLD | 64.1 | 27 | 42 | 18.5 | 2.13  (1.55-2.93) | <0.001 |
