# Supplementary material for: Uptake of infant and preschool immunisations in Scotland and England during the COVID-19 pandemic: An observational study of routinely collected data
Source: PLoS Med. 2022 Feb 22;19(2):e1003916. doi: 10.1371/journal.pmed.1003916 (PMC8863286; doi:10.1371/journal.pmed.1003916)
Supplement: S4 Table — OR and 95% CI shown are for change in uptake compared to 2019. p-Values calculated using aggregate binary logistic regression and rounded to 2 decimal places. Statistically significant change in uptake compared to 2019 are shaded green. CI, confidence interval; LD, lockdown, NA, not applicable; OR, odds ratio; SIMD, Scottish Index of Multiple Deprivation. (DOCX) [file pmed.1003916.s008.docx]

**Supplementary Table S4**

| **Immunisation** | **Deprivation quintile** | **Time period** | **% received within 4 weeks**  **(Number received/total eligible)** | **% point change from 2019** | **OR (95%CI) for uptake compared to 2019** | **p-value** |
| --- | --- | --- | --- | --- | --- | --- |
| **First 6in1** | 1 - most deprived | 2019 | 91.9  (11025/11996) | NA | NA | NA |
|  | 1 - most deprived | PreLD | 91.5  (2383/2580) | -0.4 | 1.1 (0.9-1.3) | 0.44 |
|  | 1 - most deprived | LD | 93.9  (3815/4067) | 2 | 1.3 (1.2-1.5) | <0.001 |
|  | 1 - most deprived | PostLD | 94  (1906/2026) | 2.1 | 1.4 (1.2-1.7) | <0.001 |
|  | 2 | 2019 | 93.1  (9740/10461) | NA | NA | NA |
|  | 2 | PreLD | 91.6  (2119/2289) | -1.5 | 0.9 (0.8-1.1) | 0.36 |
|  | 2 | LD | 95.1  (3311/3484) | 2 | 1.4 (1.2-1.7) | <0.001 |
|  | 2 | PostLD | 93.9  (1641/1746) | 0.8 | 1.2 (0.9-1.4) | 0.18 |
|  | 3 | 2019 | 93.7  (8457/9030) | NA | NA | NA |
|  | 3 | PreLD | 93.1  (1814/1931) | -0.6 | 1.1 (0.9-1.3) | 0.64 |
|  | 3 | LD | 95.2  (3070/3224) | 1.5 | 1.4 (1.1-1.6) | <0.001 |
|  | 3 | PostLD | 95  (1468/1546) | 1.3 | 1.3 (1-1.6) | 0.05 |
|  | 4 | 2019 | 95.6  (9804/10251) | NA | NA | NA |
|  | 4 | PreLD | 95.3  (2038/2142) | -0.3 | 0.9 (0.7-1.1) | 0.31 |
|  | 4 | LD | 95.9  (3328/3470) | 0.3 | 1.1 (0.9-1.3) | 0.5 |
|  | 4 | PostLD | 94.4  (1677/1776) | -1.2 | 0.8 (0.6-1) | 0.02 |
|  | 5 - least deprived | 2019 | 96.3  (8442/8769) | NA | NA | NA |
|  | 5 - least deprived | PreLD | 95.7  (1724/1794) | -0.6 | 1(0.7-1.3) | 0.73 |
|  | 5 - least deprived | LD | 96.7  (2759/2853) | 0.4 | 1.1 (0.9-1.4) | 0.28 |
|  | 5 - least deprived | PostLD | 96.3  (1373/1427) | 0 | 1 (0.7-1.3) | 0.92 |
| **Second 6in1** | 1 - most deprived | 2019 | 79.8  (9633/12078) | NA | NA | NA |
|  | 1 - most deprived | PreLD | 78  (1984/2492) | -1.8 | 1(0.9-1.1) | 0.87 |
|  | 1 - most deprived | LD | 86.3  (3533/4096) | 6.5 | 1.6 (1.4-1.8) | <0.001 |
|  | 1 - most deprived | PostLD | 86.3  (1737/2013) | 6.5 | 1.6 (1.4-1.8) | <0.001 |
|  | 2 | 2019 | 82.9  (8702/10499) | NA | NA | NA |
|  | 2 | PreLD | 83.4  (1880/2256) | 0.5 | 1 (0.9-1.2) | 0.61 |
|  | 2 | LD | 88.4  (3133/3549) | 5.5 | 1.6 (1.4-1.7) | <0.001 |
|  | 2 | PostLD | 86.9  (1509/1736) | 4 | 1.4 (1.2-1.6) | <0.001 |
|  | 3 | 2019 | 85.2  (7711/9055) | NA | NA | NA |
|  | 3 | PreLD | 85.4  (1675/1968) | 0.2 | 1 (0.9-1.1) | 0.96 |
|  | 3 | LD | 89.5  (2851/3182) | 4.3 | 1.5 (1.3-1.7) | <0.001 |
|  | 3 | PostLD | 89.4  (1387/1553) | 4.2 | 1.5 (1.2-1.7) | <0.001 |
|  | 4 | 2019 | 88.5  (9157/10348) | NA | NA | NA |
|  | 4 | PreLD | 89  (1892/2121) | 0.5 | 1.1 (0.9-1.3) | 0.35 |
|  | 4 | LD | 92.2  (3221/3494) | 3.7 | 1.5 (1.3-1.8) | <0.001 |
|  | 4 | PostLD | 90.2  (1540/1708) | 1.7 | 1.2 (1-1.4) | 0.04 |
|  | 5 - least deprived | 2019 | 89.1  (7925/8890) | NA | NA | NA |
|  | 5 - least deprived | PreLD | 88.5  (1655/1837) | -0.6 | 1.1 (0.9-1.3) | 0.23 |
|  | 5 - least deprived | LD | 93.3  (2668/2862) | 4.2 | 1.7 (1.4-2) | <0.001 |
|  | 5 - least deprived | PostLD | 91.7  (1280/1395) | 2.6 | 1.4 (1.1-1.7) | <0.001 |
| **Third 6in1** | 1 - most deprived | 2019 | 66.2  (8007/12102) | NA | NA | NA |
|  | 1 - most deprived | PreLD | 65.5  (1727/2640) | -0.7 | 1 (0.9-1.1) | 0.46 |
|  | 1 - most deprived | LD | 77.1  (3175/4114) | 10.9 | 1.7 (1.6-1.9) | <0.001 |
|  | 1 - most deprived | PostLD | 74.9  (1427/1905) | 8.7 | 1.5 (1.4-1.7) | <0.001 |
|  | 2 | 2019 | 70.3  (7432/10569) | NA | NA | NA |
|  | 2 | PreLD | 68.5  (1607/2331) | -1.8 | 0.9 (0.9-1) | 0.19 |
|  | 2 | LD | 80  (2836/3550) | 9.7 | 1.7 (1.5-1.8) | <0.001 |
|  | 2 | PostLD | 79.4  (1336/1683) | 9.1 | 1.6 (1.4-1.8) | <0.001 |
|  | 3 | 2019 | 73.2  (6670/9107) | NA | NA | NA |
|  | 3 | PreLD | 74.3  (1499/2045) | 1.1 | 1 (0.9-1.1) | 0.96 |
|  | 3 | LD | 82  (2610/3178) | 8.8 | 1.7 (1.5-1.9) | <0.001 |
|  | 3 | PostLD | 80.6  (1242/1542) | 7.4 | 1.5 (1.3-1.7) | <0.001 |
|  | 4 | 2019 | 77.5  (7991/10307) | NA | NA | NA |
|  | 4 | PreLD | 77.5  (1799/2322) | 0 | 1 (0.9-1.1) | 0.96 |
|  | 4 | LD | 86.2  (2911/3376) | 8.7 | 1.8 (1.6-2) | <0.001 |
|  | 4 | PostLD | 82.7  (1414/1709) | 5.2 | 1.4 (1.2-1.6) | <0.001 |
|  | 5 - least deprived | 2019 | 79.7  (7091/8893) | NA | NA | NA |
|  | 5 - least deprived | PreLD | 80.9  (1626/2032) | 1.2 | 1 (0.9-1.1) | 0.77 |
|  | 5 - least deprived | LD | 87  (2478/2841) | 7.3 | 1.7 (1.5-2) | <0.001 |
|  | 5 - least deprived | PostLD | 85.2  (1121/1316) | 5.5 | 1.5 (1.2-1.7) | <0.001 |
| **First MMR** | 1 - most deprived | 2019 | 62.4  (7561/12121) | NA | NA | NA |
|  | 1 - most deprived | PreLD | 63.8  (1790/2721) | 1.4 | 1.2 (1.1-1.3) | <0.001 |
|  | 1 - most deprived | LD | 73.7  3217/4358) | 11.3 | 1.7 (1.6-1.8) | <0.001 |
|  | 1 - most deprived | PostLD | 70.6  (1529/2168) | 8.2 | 1.4 (1.3-1.6) | <0.001 |
|  | 2 | 2019 | 63.8  (6720/10533) | NA | NA | NA |
|  | 2 | PreLD | 64.6  (1579/2378) | 0.8 | 1.1 (1-1.2) | 0.02 |
|  | 2 | LD | 76.2  (2920/3825) | 12.4 | 1.8 (1.7-2) | <0.001 |
|  | 2 | PostLD | 72.9  (1325/1814) | 9.1 | 1.5 (1.4-1.7) | <0.001 |
|  | 3 | 2019 | 64  (5936/9281) | NA | NA | NA |
|  | 3 | PreLD | 68.1  (1376/2024) | 4.1 | 1.2 (1.1-1.3) | <0.001 |
|  | 3 | LD | 77.9  (2541/3251) | 13.9 | 2 (1.8-2.2) | <0.001 |
|  | 3 | PostLD | 74.6  (1260/1685) | 10.6 | 1.7 (1.5-1.9) | <0.001 |
|  | 4 | 2019 | 66.5  (7102/10673) | NA | NA | NA |
|  | 4 | PreLD | 67.9  (1597/2292) | 1.4 | 1.2 (1-1.3) | <0.001 |
|  | 4 | LD | 80.5  (3041/3776) | 14 | 2.1 (1.9-2.3) | <0.001 |
|  | 4 | PostLD | 76.6  (1379/1801) | 10.1 | 1.6 (1.5-1.8) | <0.001 |
|  | 5 - least deprived | 2019 | 70.4  (6563/9325) | NA | NA | NA |
|  | 5 - least deprived | PreLD | 74  (1423/1927) | 3.6 | 1.2 (1.1-1.3) | <0.001 |
|  | 5 - least deprived | LD | 85.1  (2730/3208) | 14.7 | 2.4 (2.2-2.7) | <0.001 |
|  | 5 - least deprived | PostLD | 79  (1238/1566) | 8.6 | 1.6 (1.4-1.8) | <0.001 |
| **Second MMR** | 1 - most deprived | 2019 | 46  (5785/12580) | NA | NA | NA |
|  | 1 - most deprived | PreLD | 47.9  (1482/2935) | 1.9 | 1.2 (1.1-1.3) | <0.001 |
|  | 1 - most deprived | LD | 60.3  (2727/4538) | 14.3 | 1.8 (1.7-1.9) | <0.001 |
|  | 1 - most deprived | PostLD | 57.7  (1236/2139) | 11.7 | 1.6 (1.5-1.8) | <0.001 |
|  | 2 | 2019 | 50.2  (5169/10291) | NA | NA | NA |
|  | 2 | PreLD | 53.3  (1270/2300) | 3.1 | 1.2 (1.1-1.3) | <0.001 |
|  | 2 | LD | 63.7  (2276/3570) | 13.5 | 1.7 (1.6-1.9) | <0.001 |
|  | 2 | PostLD | 60.3  (1001/1661) | 10.1 | 1.5 (1.4-1.7) | <0.001 |
|  | 3 | 2019 | 51.7  (4701/9100) | NA | NA | NA |
|  | 3 | PreLD | 51.8  (1127/2099) | 0.1 | 1.2 (1.1-1.3) | <0.001 |
|  | 3 | LD | 64.5  (1937/3007) | 12.8 | 2 (1.8-2.2) | <0.001 |
|  | 3 | PostLD | 62.3  (916/1470) | 10.6 | 1.7 (1.5-1.9) | <0.001 |
|  | 4 | 2019 | 54.2  (4998/9219) | NA | NA | NA |
|  | 4 | PreLD | 53.3  (1219/2135) | -0.9 | 1.1 (1-1.2) | 0.02 |
|  | 4 | LD | 69.8  (2238/3211) | 15.6 | 1.9 (1.8-2.1) | <0.001 |
|  | 4 | PostLD | 66.4  (1014/1528) | 12.2 | 1.7 (1.5-1.9) | <0.001 |
|  | 5 - least deprived | 2019 | 59.4  (5148/8670) | NA | NA | NA |
|  | 5 - least deprived | PreLD | 61.6  (1281/2010) | 2.2 | 1.2 (1.1-1.3) | <0.001 |
|  | 5 - least deprived | LD | 75.6  (2105/2787) | 16.2 | 2.1 (1.9-2.3) | <0.001 |
|  | 5 - least deprived | PostLD | 72  (1000/1391) | 12.6 | 1.7 (1.5-2) | <0.001 |

Table S4: Uptake of pre-school immunisations by time period and SIMD and percent point change in uptake compared to baseline 2019. Odds ratio and 95% confidence intervals shown are for change in uptake compared to 2019. *p*-values calculated using aggregate binary logistic regression and rounded to 2 decimal places. LD = lockdown, NA = not applicable. Statistically significant change in uptake compared to 2019 are shaded green.
