# Supplementary material for: Uptake of infant and preschool immunisations in Scotland and England during the COVID-19 pandemic: An observational study of routinely collected data
Source: PLoS Med. 2022 Feb 22;19(2):e1003916. doi: 10.1371/journal.pmed.1003916 (PMC8863286; doi:10.1371/journal.pmed.1003916)
Supplement: S5 Table — The baseline comparisons showed are for time period 2019 and deprivation quintile SIMD 1. If the 95% CI did not include 1, the interaction of time period and SIMD was considered statistically significant, that is; there was a significant difference in the level of change (2019- time period) between the deprivation quintile and SIMD 1. For example, the increase in uptake during lockdown for SIMD 5 was statistically greater than the increase in uptake for SIMD 1. The ROR can be used to calculate the OR for uptake compared to the baseline levels by multiplying the ROR with the relevant OR in S3 table. p-Values calculated using aggregate binary logistic regression and rounded to 2 decimal places. CI, confidence interval; LD, lockdown; ns, not statistically significant (coloured green), interaction was statistically significant; OR, odds ratio; ROR, ratio of odds ratio, calculated by taking the exponential function of the coefficient of the interaction term from the interaction model; SIMD, Scottish Index of Multiple Deprivation. (DOCX) [file pmed.1003916.s009.docx]

**Supplementary Table S5**

| **Immunisation** | **Interaction term**  **(baseline comparisons = 2019, SIMD 1)** | **ROR**  **(exp of coeff of interaction model)** | **95% Confidence intervals** | ***p* -value** |
| --- | --- | --- | --- | --- |
| **First 6in1** | PreLD:SIMD2 | 0.87 | (0.68-1.1)  ns | 0.23 |
|  | LD:SIMD2 | 1.06 | (0.85-1.33)  ns | 0.59 |
|  | PostLD:SIMD2 | 0.83 | (0.62-1.1)  ns | 0.2 |
|  | PreLD:SIMD3 | 0.99 | (0.76-1.28)  ns | 0.92 |
|  | LD:SIMD3 | 1.01 | (0.8-1.28)  ns | 0.91 |
|  | PostLD:SIMD3 | 0.91 | (0.67-1.25)  ns | 0.56 |
|  | PreLD:SIMD4 | 0.84 | (0.64-1.1)  ns | 0.2 |
|  | LD:SIMD4 | 0.8 | (0.63-1.02)  ns | 0.07 |
|  | PostLD:SIMD4 | 0.55 | (0.41-0.74)  sig | <0.001 |
|  | PreLD:SIMD5 | 0.9 | (0.66-1.22)  ns | 0.48 |
|  | LD:SIMD5 | 0.85 | (0.65-1.12)  ns | 0.25 |
|  | PostLD:SIMD5 | 0.7 | (0.5-1.01)  ns | 0.05 |
| **Second 6in1** | PreLD:SIMD2 | 1.04 | (0.89-1.23)  ns | 0.62 |
|  | LD:SIMD2 | 0.98 | (0.84-1.14)  ns | 0.76 |
|  | PostLD:SIMD2 | 0.86 | (0.7-1.05)  ns | 0.14 |
|  | PreLD:SIMD3 | 1.01 | (0.85-1.2)  ns | 0.95 |
|  | LD:SIMD3 | 0.94 | (0.8-1.11)  ns | 0.47 |
|  | PostLD:SIMD3 | 0.91 | (0.73-1.13)  ns | 0.41 |
|  | PreLD:SIMD4 | 1.08 | (0.9-1.3)  ns | 0.39 |
|  | LD:SIMD4 | 0.96 | (0.81-1.14)  ns | 0.67 |
|  | PostLD:SIMD4 | 0.75 | (0.6-0.93)  sig | 0.01 |
|  | PreLD:SIMD5 | 1.12 | (0.92-1.36)  ns | 0.27 |
|  | LD:SIMD5 | 1.05 | (0.87-1.27)  ns | 0.6 |
|  | PostLD:SIMD5 | 0.85 | (0.67-1.08)  ns | 0.18 |
| **Third 6in1** | PreLD:SIMD2 | 0.97 | (0.85-1.1)  ns | 0.63 |
|  | LD:SIMD2 | 0.97 | (0.86-1.1)  ns | 0.62 |
|  | PostLD:SIMD2 | 1.06 | (0.9-1.26)  ns | 0.46 |
|  | PreLD:SIMD3 | 1.04 | (0.9-1.19)  ns | 0.61 |
|  | LD:SIMD3 | 0.97 | (0.85-1.11)  ns | 0.66 |
|  | PostLD:SIMD3 | 0.99 | (0.83-1.18)  ns | 0.92 |
|  | PreLD:SIMD4 | 1.03 | (0.9-1.19)  ns | 0.67 |
|  | LD:SIMD4 | 1.05 | (0.92-1.2)  ns | 0.49 |
|  | PostLD:SIMD4 | 0.91 | (0.77-1.08)  ns | 0.29 |
|  | PreLD:SIMD5 | 1.05 | (0.91-1.22)  ns | 0.51 |
|  | LD:SIMD5 | 1 | (0.87-1.16)  ns | 0.97 |
|  | PostLD:SIMD5 | 0.96 | (0.79-1.16)  ns | 0.66 |
| **First MMR** | PreLD:SIMD2 | 0.97 | (0.85-1.1)  ns | 0.61 |
|  | LD:SIMD2 | 1.08 | (0.96-1.21)  ns | 0.21 |
|  | PostLD:SIMD2 | 1.07 | (0.92-1.24)  ns | 0.4 |
|  | PreLD:SIMD3 | 1.03 | (0.9-1.18)  ns | 0.65 |
|  | LD:SIMD3 | 1.19 | (1.05-1.34)  sig | 0.01 |
|  | PostLD:SIMD3 | 1.16 | (0.99-1.35)  ns | 0.06 |
|  | PreLD:SIMD4 | 1 | (0.87-1.14)  ns | 0.96 |
|  | LD:SIMD4 | 1.22 | (1.09-1.38)  sig | <0.001 |
|  | PostLD:SIMD4 | 1.14 | (0.98-1.33)  ns | 0.1 |
|  | PreLD:SIMD5 | 1.02 | (0.89-1.18)  ns | 0.73 |
|  | LD:SIMD5 | 1.41 | (1.24-1.61)  sig | <0.001 |
|  | PostLD:SIMD5 | 1.1 | (0.94-1.3)  ns | 0.25 |
| **Second MMR** | PreLD:SIMD2 | 1.02 | (0.9-1.15)  ns | 0.75 |
|  | LD:SIMD2 | 0.99 | (0.89-1.09)  ns | 0.78 |
|  | PostLD:SIMD2 | 0.93 | (0.81-1.08)  ns | 0.35 |
|  | PreLD:SIMD3 | 0.91 | (0.8-1.03)  ns | 0.12 |
|  | LD:SIMD3 | 0.96 | (0.86-1.07)  ns | 0.44 |
|  | PostLD:SIMD3 | 0.96 | (0.83-1.11)  ns | 0.61 |
|  | PreLD:SIMD4 | 0.94 | (0.83-1.06)  ns | 0.31 |
|  | LD:SIMD4 | 1.1 | (0.98-1.23)  ns | 0.09 |
|  | PostLD:SIMD4 | 1.04 | (0.9-1.2)  ns | 0.63 |
|  | PreLD:SIMD5 | 1 | (0.88-1.14)  ns | 0.96 |
|  | LD:SIMD5 | 1.19 | (1.06-1.34)  sig | <0.001 |
|  | PostLD:SIMD5 | 1.09 | (0.93-1.27)  ns | 0.29 |

Table S5: To assess whether the differences between change in uptake were statistically significant between SIMD quintiles, the interaction between time period and SIMD quintile was added into the model. The baseline comparisons showed are for time period 2019 and deprivation quintile SIMD 1. ROR = ratio of odds ratio, calculated by taking the exponential function of the coefficient of the interaction term from the interaction model. If the 95% confidence intervals did not include 1, the interaction of time period and SIMD was considered statistically significant, that is; there was a significant difference in the level of change (2019- time period) between the deprivation quintile and SIMD 1. For example, the increase in uptake during lockdown for SIMD 5 was statistically greater than the increase in uptake for SIMD 1. The ROR can be used to calculate the odds ratio for uptake compared to the baseline levels by multiplying the ROR with the relevant OR in table S4. LD= lockdown, SIMD = Scottish Index of Multiple Deprivation, ns = not statistically significant (coloured green) = interaction was statistically significant. *p*-values calculated using aggregate binary logistic regression and rounded to 2 decimal places.
