# Supplementary material for: Uptake of infant and preschool immunisations in Scotland and England during the COVID-19 pandemic: An observational study of routinely collected data
Source: PLoS Med. 2022 Feb 22;19(2):e1003916. doi: 10.1371/journal.pmed.1003916 (PMC8863286; doi:10.1371/journal.pmed.1003916)
Supplement: S6 Table — Uptake of preschool immunisations at an older age by time period and point percentage change from 2019 with OR and 95% CI compared to baseline of 2019. Children are categorised into the time period at which they became eligible for the immunisation as before and uptake data were extracted at a later stage when they reached the ages indicated in the immunisation column. Statistically significant changes are coloured green. p-Values calculated using aggregate binary logistic regression and rounded to 2 decimal places. CI, confidence interval; LD, lockdown; NA, not applicable; OR, odds ratio. (DOCX) [file pmed.1003916.s010.docx]

**Supplementary table S6**

| **Immunisation** | **Time period** | **% uptake**  **(no received/no eligible)** | **% point change from 2019** | **OR for uptake compared to 2019**  **(95% CI)** | ***p*-value** |
| --- | --- | --- | --- | --- | --- |
| First6in1  (uptake by age 24weeks) | 2019 | 97.9  (49542/50609) | NA | NA | NA |
|  | Pre LD | 97.7  (10514/10761) | -0.2 | 0.92  (0.8-1.06) | 0.22 |
|  | LD | 97.6  (16724/17133) | -0.3 | 0.88  (0.79-0.99) | 0.03 |
|  | Post LD | 97.5  (8319/8531) | -0.4 | 0.85  (0.73-0.98) | 0.03 |
| Second6in1  (uptake by age 28 weeks) | 2019 | 96.7  (49291/50975) | NA | NA | NA |
|  | Pre LD | 96.2  (10306/10698) | -0.5 | 0.9  (0.8-1.01) | 0.06 |
|  | LD | 96.6  (16639/17222) | -0.1 | 0.98  (0.89-1.07) | 0.61 |
|  | Post LD | 96.6  (8125/8412) | -0.1 | 0.97  (0.85-1.1) | 0.61 |
| Third6in1  (uptake by age 32 weeks) | 2019 | 94  (48029/51085) | NA | NA | NA |
|  | Pre LD | 94.1  (10728/11394) | 0.1 | 1.02  (0.94-1.12) | 0.59 |
|  | LD | 94.8  (16199/17093) | 0.8 | 1.15  (1.07-1.24) | <0.001 |
|  | Post LD | 93.5  (7644/8172) | -0.5 | 0.92  (0.84-1.01) | 0.09 |
| FirstMMR  (uptake by age 16 months) | 2019 | 91.1  (47386/52015) | NA | NA | NA |
|  | Pre LD | 91.3  (10389/11370) | 0.2 | 1.03  (0.96-1.11) | 0.36 |
|  | LD | 92.5  (17076/18463) | 1.4 | 1.2  (1.13-1.28) | <0.001 |
|  | Post LD | 91.6  (8285/9047) | 0.5 | 1.06  (0.98-1.15) | 0.14 |
| SecondMMR  (uptake by age 3years 8 months) | 2019 | 80.8  (40376/49940) | NA | NA | NA |
|  | Pre LD | 83.2  (9471/11495) | 2.4 | 1.11  (1.05-1.17) | <0.001 |
|  | LD | 86.1  (14763/17145) | 5.3 | 1.47  (1.4-1.54) | <0.001 |
|  | Post LD | 84.4  (6915/8196) | 3.6 | 1.28  (1.2-1.36) | <0.001 |

Table S6: Scotland. Uptake of pre-school immunisations at an older age by time period and point percentage change from 2019 with odds ratio and 95% confidence intervals compared to baseline of 2019. Children are categorised into the time-period at which they became eligible for the immunisation as before and uptake data were extracted at a later stage when they reached the ages indicated in the immunisation column. LD = lockdown, NA = not applicable. Statistically significant changes are coloured green. *p*-values calculated using aggregate binary logistic regression and rounded to 2 decimal places.
