# Supplementary material for: Uptake of infant and preschool immunisations in Scotland and England during the COVID-19 pandemic: An observational study of routinely collected data
Source: PLoS Med. 2022 Feb 22;19(2):e1003916. doi: 10.1371/journal.pmed.1003916 (PMC8863286; doi:10.1371/journal.pmed.1003916)
Supplement: S7 Table — Uptake of preschool immunisations at an older age by time period and point percentage change from 2019 with OR and 95% CI compared to baseline of 2019. Children are categorised into the time period at which they became eligible for the immunisation as before and uptake data were extracted at a later stage when they reached the ages indicated in the immunisation column. Statistically significant changes are coloured green. p-Values calculated using aggregate binary logistic regression and rounded to 2 decimal places. CI, confidence interval; LD, lockdown; NA, not applicable; OR, odds ratio. (DOCX) [file pmed.1003916.s011.docx]

**Supplementary table S7**

| **Immunisation** | **Time period** | **% uptake**  **(no received/no eligible)** | **% point change from 2019** | **OR for uptake compared to 2019**  **(95% CI)** | ***p*-value** |
| --- | --- | --- | --- | --- | --- |
| First6in1  (uptake by age 6 months) | 2019 | 96.3  (571531/593700) | NA | NA | NA |
|  | Pre LD | 95.8  (132779/138608) | 0.5 | 0.88 (0.86-0.91) | <0.001 |
|  | LD | 95.8  (171660/179180) | 0.5 | 0.89 (0.86-0.91) | <0.001 |
|  | Post LD | 95.6  (90702/94862) | 0.7 | 0.85 (0.82 -0.87) | <0.001 |
| Second6in1  (uptake by age 6 months) | 2019 | 93.9  (559382/595815) | NA | NA | NA |
|  | Pre LD | 92.1  (131578/142833) | 1.8 | 0.76 (0.74-0.78) | <0.001 |
|  | LD | 93.0  (164780/177221) | 0.9 | 0.86 (0.84-0.88) | <0.001 |
|  | Post LD | 92.5  (87818/94960) | 1.4 | 0.80 (0.78-0.82) | <0.001 |
| Third6in1  (uptake by age 6 months) | 2019 | 88.7  (529163/596355) | NA | NA | NA |
|  | Pre LD | 85.6  (126284/147479) | 3.1 | 0.76 (0.74-0.77) | <0.001 |
|  | LD | 86.6  (154912/178953) | 2.1 | 0.82 (0.81-0.83) | <0.001 |
|  | Post LD | 86.0  (79991/92990) | 2.7 | 0.78 (0.77-0.80) | <0.001 |
| FirstMMR  (uptake by age 18 months) | 2019 | 88.1  (541107/613923) | NA | NA | NA |
|  | Pre LD | 86.7  (119885/138353) | 1.4 | 0.87(0.86-0.89) | <0.001 |
|  | LD | 86.2  (173451/201164) | 1.9 | 0.84(0.83-0.85) | <0.001 |
|  | Post LD | 86.2  (85939/99685) | 1.9 | 0.84(0.83-0.86) | <0.001 |

Table S7: England. Uptake of pre-school immunisations at an older age by time period and point percentage change from 2019 with odds ratio and 95% confidence intervals compared to baseline of 2019. Children are categorised into the time-period at which they became eligible for the immunisation as before and uptake data were extracted at a later stage when they reached the ages indicated in the immunisation column. LD = lockdown, NA = not applicable. Statistically significant changes are coloured green. *p*-values calculated using aggregate binary logistic regression and rounded to 2 decimal places.
