# Supplementary material for: Uptake of infant and preschool immunisations in Scotland and England during the COVID-19 pandemic: An observational study of routinely collected data
Source: PLoS Med. 2022 Feb 22;19(2):e1003916. doi: 10.1371/journal.pmed.1003916 (PMC8863286; doi:10.1371/journal.pmed.1003916)
Supplement: Attachment — Submitted filename: PMEDICINE-D-21-03163_R1_reviewed.pdf [file pmed.1003916.s015.pdf]

PLOS Medicine

Uptake of infant and pre-school immunisations in Scotland and England during the COVID-19 pandemic: an observational study of routinely collected data

--Manuscript Draft--

**Manuscript Number:**	PMEDICINE-D-21-03163R1
**Full Title:**	Uptake of infant and pre-school immunisations in Scotland and England during the COVID-19 pandemic: an observational study of routinely collected data
**Short Title:**	Uptake of routine childhood immunisation during the COVID-19 pandemic
**Article Type:**	Research Article
**Abstract:**	Background<br>In 2020, the COVID-19 pandemic and control measures such as national lockdowns threatened to disrupt routine childhood immunisation programmes. Initial reports from the early weeks of lockdown in the UK and worldwide suggested that uptake could fall putting children at risk from multiple other infectious diseases. In Scotland and England, enhanced surveillance of national data for childhood immunisations was established to inform and rapidly assess the impact of the pandemic on infant and preschool immunisation uptake rates.<br>Methods and findings<br>We undertook an observational study using routinely collected data for the year prior to the pandemic (2019), and immediately before, during and after the first period of the UK 'lockdown' in 2020. Data were obtained for Scotland from the Public Health Scotland "COVID19 wider impacts on the health care system" dashboard (https://scotland.shinyapps.io/phs-covid-wider-impact/) and for England from ImmForm.<br>Five vaccinations delivered at different ages were evaluated; three doses of the '6-in-1' DTaP/IPV/Hib/HepB vaccine and two doses of MMR. Uptake in the periods in 2020 compared to that in the baseline year of 2019 using binary logistic regression analysis. For Scotland, we analysed timely uptake of immunisations, defined as uptake within four weeks of the child becoming eligible by age for each immunisation and data were also analysed by geographical region and indices of deprivation. For both Scotland and England, we assessed whether immunisations were up to date at approximately 6 months (all doses 6-in-1) and 16-18 months (first MMR) of age.<br>We found that uptake rates within four weeks of eligibility in Scotland for all the five vaccine visits were higher during the 2020 lockdown period than in 2019. The difference ranged from 1.3% for the first dose of the 6-in-1 vaccine (95.3 vs 94%, OR 1.28, CI 1.18-1.39) to 14.3% for the second MMR dose (66.1 vs 51.8 %, OR 1.8, CI 1.74-1.87). Significant increases in uptake were seen across all deprivation levels, though, for MMR, there was evidence of greater improvement for children living in the least deprived areas.<br>In England, fewer children who had been due to receive their immunisations during the lockdown period were up to date at 6 months (6-in-1) or 18 months (first dose MMR). The fall in percentage uptake ranged from 0.5% for first 6-in1 (95.8 vs 96.3%, OR 0.89, CI 0.86-0.91) to 2.1% for third 6-in-1 (86.6 vs 88.7%, OR 0.82, CI 0.81-0.83).<br>Conclusions<br>This study suggests that the national lockdown in Scotland was associated with a positive effect on timely childhood immunisation uptake, however in England a lower percentage of children were up to date at 6 and 18 months. Reason for the improve uptake in Scotland may include active measures taken to promote immunisation at local and national level during this period. Promoting immunisation uptake and addressing potential vaccine hesitancy is particularly important given the ongoing pandemic and COVID-19 vaccination campaigns.
**Additional Information:**	
**Question**	**Response**
**Financial Disclosure**<br><br>Enter a financial disclosure statement that	The author received no specific funding for this work. RM is funded by the Health Data Research UK BREATHE hub who had no role in study design, data collection and analysis, decision to publish, or preparation of the manuscript.

Powered by Editorial Manager® and ProduXion Manager® from Aries Systems Corporation

describes the sources of funding for the work included in this submission. Review the submission guidelines for detailed requirements. View published research articles from *PLOS Medicine* for specific examples.<br><br>This statement is required for submission and **will appear in the published article** if the submission is accepted. Please make sure it is accurate.<br><br>**Unfunded studies**<br>Enter: *The author(s) received no specific funding for this work.*<br><br>**Funded studies**<br>Enter a statement with the following details:<br>• Initials of the authors who received each award<br>• Grant numbers awarded to each author<br>• The full name of each funder<br>• URL of each funder website<br>• Did the sponsors or funders play any role in the study design, data collection and analysis, decision to publish, or preparation of the manuscript?<br>• **NO** - Include this sentence at the end of your statement: *The funders had no role in study design, data collection and analysis, decision to publish, or preparation of the manuscript.*<br>• **YES** - Specify the role(s) played.<br><br>* typeset	
**Competing Interests**<br><br>Use the instructions below to enter a competing interest statement for this submission. On behalf of all authors, disclose any competing interests that could be perceived to bias this work—acknowledging all financial support and any other relevant financial or non-financial competing interests.<br><br>This statement **will appear in the published article** if the submission is accepted. Please make sure it is	I have read the journal's policy and the authors of this manuscript have the following ompeting interests: AS is a member of the Scottish Government Chief Medical Officer’s COVID-19 Advisory Group. All other authors declare no competing interests.

accurate. View published research articles from *PLOS Medicine* for specific examples.<br><br>**NO authors have competing interests**<br><br>Enter: *The authors have declared that no competing interests exist.*<br><br>**Authors with competing interests**<br>Enter competing interest details beginning with this statement:<br><br>*I have read the journal's policy and the authors of this manuscript have the following competing interests: [insert competing interests here]*<br><br>* typeset<br><br>This statement is required for submission and **will appear in the published article** if the submission is accepted. Please make sure it is accurate and that any funding sources listed in your Funding Information later in the submission form are also declared in your Financial Disclosure statement.	
**Ethics Statement**<br><br>You must include an Ethics Statement in the Methods section of your manuscript if your study involved:<br>• Human participants<br>• Human specimens or tissue<br>• Vertebrate animals or cephalopods<br>• Vertebrate embryos or tissues<br>• Field research<br><br>**Not including a statement when required will delay the review process.**<br><br>General guidance is provided below.	I confirm that I have read and understood PLOS' requirements for an Ethics Statement.

Please consult the guidelines on human subjects research and animal research for detailed instructions.<br><br>**Format for specific study types**<br><br>**Human Subject Research (involving human participants and/or tissue)**<br>• Give the name of the institutional review board or ethics committee that approved the study<br>• Include the approval number and/or a statement indicating approval of this research<br>• Indicate the form of consent obtained (written/oral) or the reason that consent was not obtained (e.g. the data were analyzed anonymously)<br><br>**Animal Research (involving vertebrate animals, embryos or tissues)**<br>• Provide the name of the Institutional Animal Care and Use Committee (IACUC) or other relevant ethics board that reviewed the study protocol, and indicate whether they approved this research or granted a formal waiver of ethical approval<br>• Include an approval number if one was obtained<br>• If the study involved *non-human primates*, add *additional details* about animal welfare and steps taken to ameliorate suffering<br><br>**Field Research**<br><br>Include the following details if this study involves the collection of plant, animal, or other materials from a natural setting:<br>• Field permit number<br>• Name of the institution or relevant body that granted permission<br><br>Please check the box to confirm your understanding of this policy, then read and agree to one of the statements.	

Please respond:<br>as follow-up to "**Ethics Statement**<br><br>You must include an Ethics Statement in the Methods section of your manuscript if your study involved:<br>• Human participants<br>• Human specimens or tissue<br>• Vertebrate animals or cephalopods<br>• Vertebrate embryos or tissues<br>• Field research<br><br>**Not including a statement when required will delay the review process.**<br><br>General guidance is provided below. Please consult the guidelines on human subjects research and animal research for detailed instructions.	My study does not require an ethics statement.

Powered by Editorial Manager® and ProduXion Manager® from Aries Systems Corporation

**Format for specific study types**<br><br>**Human Subject Research (involving human participants and/or tissue)**<br>• Give the name of the institutional review board or ethics committee that approved the study<br>• Include the approval number and/or a statement indicating approval of this research<br>• Indicate the form of consent obtained (written/oral) or the reason that consent was not obtained (e.g. the data were analyzed anonymously)<br><br>**Animal Research (involving vertebrate animals, embryos or tissues)**<br>• Provide the name of the Institutional Animal Care and Use Committee (IACUC) or other relevant ethics board that reviewed the study protocol, and indicate whether they approved this research or granted a formal waiver of ethical approval<br>• Include an approval number if one was obtained<br>• If the study involved *non-human primates*, add *additional details* about animal welfare and steps taken to ameliorate suffering<br><br>**Field Research**<br><br>Include the following details if this study involves the collection of plant, animal, or other materials from a natural setting:<br>• Field permit number<br>• Name of the institution or relevant body that granted permission<br><br>Please check the box to confirm your understanding of this policy, then read and agree to one of the statements. "	
**Data Availability**<br><br>Authors are required to make all data underlying the findings described fully available, without restriction, and from the time of publication. PLOS allows rare exceptions to address legal and ethical concerns. See the PLOS Data Policy and	No - some restrictions will apply

FAQ for detailed information.<br><br>A Data Availability Statement describing where the data can be found is required at submission. Your answers to this question constitute the Data Availability Statement and **will be published in the article**, if accepted.<br><br>**Important:** Stating 'data available on request from the author' is not sufficient. If your data are only available upon request, select 'No' for the first question and explain your exceptional situation in the text box.<br><br>Do the authors confirm that all data underlying the findings described in their manuscript are fully available without restriction?	
**Describe where the data may be found in full sentences. If you are copying our sample text, replace any instances of XXX with the appropriate details.**<br><br>• If the data are **held or will be held in a public repository**, include URLs, accession numbers or DOIs. If this information will only be available after acceptance, indicate this by ticking the box below. For example: *All XXX files are available from the XXX database (accession number(s) XXX, XXX.*).<br>• If the data are all contained **within the manuscript and/or Supporting Information files**, enter the following: *All relevant data are within the manuscript and its Supporting Information files.*<br>• If neither of these applies but you are able to provide **details of access elsewhere**, with or without limitations, please do so. For example:<br><br>*Data cannot be shared publicly because of [XXX]. Data are available from the XXX Institutional Data Access / Ethics Committee (contact via XXX) for*	All relevant data are within the manuscript and its Supporting Information files and further Scottish data can be accessed via the Public health Scotland wider impacts dashboard https://scotland.shinyapps.io/phs-covid-wider-impact/. Aggregated English data could be made available if in agreement with information governance regulations and upon request from the authors. All code used in the analyses will be made publicly available via the EAVE II GitHub page (https://github.com/EAVE-II) .

researchers who meet the criteria for access to confidential data.<br><br>*The data underlying the results presented in the study are available from (include the name of the third party and contact information or URL).*<br>• This text is appropriate if the data are owned by a third party and authors do not have permission to share the data.<br><br>* typeset	
Additional data availability information:	

Powered by Editorial Manager® and ProduXion Manager® from Aries Systems Corporation

Manuscript

Click here to access/download;Manuscript;Impact of COVID on pre-school immunisations PLOSMed.docx

Full Title

Uptake of infant and pre-school immunisations in Scotland and England during the COVID-19 pandemic: an observational study of routinely collected data

Short Title

Uptake of routine childhood immunisation during the COVID-19 pandemic

Authors

Fiona McQuaid[1*]. Rachel Mulholland [2]. Yuma Sangpang Rai [3], Utkarsh Agrawal[4], Helen Bedford[5], J. Claire Cameron[6], Cheryl Gibbons[6], Partho Roy[3], Aziz Sheikh[2], Ting Shi[2], Colin R Simpson[2,7], Judith Tait[6], Elise Tessier[3], Steve Turner[8], Jaime Villacampa Ortega[6], Joanne White[3], Rachael Wood[2,6]

[1]Department of Child Life and Health, University of Edinburgh, Edinburgh, UK

[2]Usher Institute, University of Edinburgh, Edinburgh, UK

[3]Immunisation and Countermeasures Division, National Infections Service, Public Health England, London, UK

[4]School of Medicine, University of St Andrews, St Andrews, UK

[5]UCL Great Ormond Street Institute of Child Health, London, UK

[6]Public Health Scotland, Glasgow and Edinburgh, UK

[7]School of Health, Wellington Faculty of Health, Wellington, NZ

[8]Women and Children Division, NHS Grampian, Aberdeen, UK

***Corresponding author:**

Email: Fiona.mcquaid@ed.ac.uk (FM)

Contributor information

The study was conceived and designed by FM, RW and RM. The data were analysed by FM, RM, YR with oversight from RW and ET. FM drafted the manuscript. JT and JVO were responsible for the Public Health Scotland wider impacts of COVID-19 database from which the Scottish data were extracted. All authors have reviewed the statistical analysis plan and approved the manuscript.

Competing interests

All authors have completed the ICMJE uniform disclosure form at www.icmje.org/coi_disclosure.pdf . AS is a member of the Scottish Government Chief Medical Officer's COVID-19 Advisory Group. RM is funded by the Health Data Research UK BREATHE hub. All other authors declare no competing interests

Data sharing statement

All data used in this study are publicly available on the Public Health Scotland "Wider impacts of COVID-19" dashboard which can be accessed via https://scotland.shinyapps.io/phs-covid-wider-impact/. The code used to analyse the data is publicly available via the EAVE II GitHub (https://github.com/EAVE-II).

Abstract

Background

In 2020, the COVID-19 pandemic and control measures such as national lockdowns threatened to disrupt routine childhood immunisation programmes. Initial reports from the early weeks of lockdown in the UK and worldwide suggested that immunisation uptake would fall, putting children at risk from multiple other infectious diseases. In Scotland and England, enhance[illegible]rveillance of national data for childhood immunisations was established to inform and rapidly assess the impact of the pandemic on infant and preschool immunisation uptake rates.

Methods and findings

We undertook an observational study using routinely collected data for the year prior to the pandemic (2019), and immediately before, during and after the fi[illegible]eriod of the UK ‘lockdown’ in 2020. Data were obtained for Scotland from the Public Health Scotland “COVID19 wider impacts on the health care system” dashboard (https://scotland.shinyapps.io/phs-covid-wider-impact/) and for England from ImmForm.

Five vaccinations delivered at different ages were evaluated; three doses of the ‘6-in-1’ DTaP/IPV/Hib/HepB vaccine and two doses of MMR. Uptake in the periods in 2020 compared to that in the baseline year of 2019 using binary logistic regression analysis. For Scotland, we analysed timely uptake of immunisations, defined as uptake within four weeks of the child becoming eligible by age for each immunisation and data were also analysed by geographical region and indices of deprivation. For both Scotland and England, we assessed whether immunisations were up to date at approximately 6 months (all doses 6-in-1) and 16-18 months (first MMR) of age.

We found that uptake rates within four weeks of eligibility in Scotland for all the five vaccine visits were higher during the 2020 lockdown period than in 2019. The difference ranged from 1.3% for the first dose of the 6-in-1 vaccine (95.3 vs 94%, OR 1.28, CI 1.18-1.39) to 14.3% for the second MMR dose (66.1 vs 51.8 %, OR 1.8, CI 1.74-1.87). Significant increases in uptake were seen across all deprivation levels, though, for MMR, there was evidence of greater improvement for children living in the least deprived areas.

In England, fewer children who had been due to receive their immunisations during the lockdown period were up to date at 6 months (6-in-1) or 18 months (first dose MMR). The fall in percentage uptake ranged from 0.5% for first 6-in1 (95.8 vs 96.3%, OR 0.89, CI 0.86-0.91) to 2.1% for third 6-in-1 (86.6 vs 88.7%, OR 0.82, CI 0.81-0.83).

Conclusions

This study suggests that the national lockdown in Scotland was associated with a positive effect on timely childhood immunisation uptake, however in England a lower percentage of children were up to date at 6 and 18 months. Reason for the improve uptake in Scotland may include active measures taken to promote immunisation at local and national level during this period. Promoting immunisation uptake and addressing potential vaccine hesitancy is particularly important given the ongoing pandemic and COVID-19 vaccination campaigns.

Introduction

The COVID-19 pandemic and associated control measures such as national 'lockdowns', involving varying restrictions on leaving the home, work and socialising, have had a profound impact on daily life and the delivery of healthcare worldwide. In the UK, a national lockdown was announced on the 23rd March 2020 with instructions that people should only leave their home for a limited number of "essential" reasons. (1) This was accompanied by the reconfiguration of acute healthcare services to support the anticipated influx of COVID-19 patients, cancellation of most elective activity and pausing of screening programmes. (2) During lockdown, there was evidence of a change in healthcare-seeking behaviour – for example, in Scotland the uptake of both emergency and elective hospital based care dropped substantially over the lockdown period. (3) However, within child health, key routine services including childhood immunisations continued through the UK. (4)

It has become increasingly apparent that younger children are at low risk of severe disease due to Severe acute respiratory syndrome coronavirus 2 (SARS-CoV-2) (5) and may be less susceptible to infection with the virus. (6) Yet the wider impact of the pandemic on children in terms of education, mental and physical health and safeguarding is not yet fully understood and is likely to be profound. (7, 8) One particular area of concern early in the lockdown period was the potential effect on the uptake of routine childhood immunisations. (9) Maintaining high population vaccine coverage is vital for both direct and indirect (via herd immunity) protection against non-COVID-19 infectious diseases.

In July 2020, the World Health Organization (WHO) warned of a potential decline in routine immunisation rates associated with the COVID-19 pandemic, citing a poll from May 2020 in which respondents from 82 countries suggested disruption to immunisation programmes was widespread (10). Initial reports from England, (11) Pakistan (12) South Africa, (13)

Singapore (14) and the USA, (15) were concerning, suggesting a fall in children receiving their scheduled vaccinations in the very early weeks of national lockdowns, though the full impact has not yet been assessed. However, the English and US studies relied on surrogate measures of vaccine uptake; number of vaccines delivered/ordered (without a corresponding denominator) (11, 15) and the Singaporean study used convenience sampling with multiple assumptions for missing data (14). Longer-term data were available from the KwaZulu-Natal province of South Africa (13) and Sindh province of Pakistan (12), which appeared to show some recovery after an initial fall in uptake; however, in Pakistan, part of the lockdown restrictions involved shutting down of outreach immunisation programme. The overall impact of lockdown on immunisation uptake in higher income countries which maintained their routine immunisation programmes is unclear.

Given the prolonged, and repeated periods of lockdown, it is important to evaluate the overall effect on childhood immunisation uptake. The aim of this study was therefore to provide a longer perspective than previous studies by describing the pattern of childhood immunisation uptake in Scotland and England before, during and immediately after the first national lockdown implemented in response to the pandemic (23rd March-31st July), with comparisons to baseline data from 2019, by geographical area and socio-economic deprivation.

Methods

Study design

This observational study took advantage of the natural experiment afforded by the COVID-19 pandemic and used routinely collected data for the year prior to the pandemic (2019) and immediately before, during and after the first period of ‘lockdown’ imposed by the United Kingdom and Scottish governments in 2020. Data were available for Scotland and England, however, as discussed below, variations in time points at which the data were collected precluded direct comparisons. Of note, this analysis relates to the first national lockdown which began on 23rd March 2020 with restrictions easing gradually from over June and July 2020.

The vaccines chosen as indicators of preschool immunisation uptake were the hexavalent DTaP/IPV/Hib/HepB vaccine, (referred to here as ‘6in1’), which protects against Diphtheria, Tetanus, Pertussis, Polio, *Haemophilus influenzae* type b and Hepatitis B, and the Measles, Mumps and Rubella vaccine (referred to as ‘MMR’). In the UK, the 6in1 vaccine is recommended at age eight (‘first dose 6in1’), 12 (‘second dose 6in1’) and 16 (‘third dose 6in1’) weeks of age, and MMR is given at 12 months (‘first dose MMR’) and 3 years 4 months (‘second dose MMR’) (16). Uptake of the additional immunisations offered at the same ages (Meningococcal C, Rotavirus and Pneumococcal) was not directly measured.

For Scotland, we chose to primarily examine uptake within four weeks of eligibility as this represents timely uptake of vaccinations as per the recommended UK schedule (16) leading to the child being protected at the earliest recommended opportunity. All children living in Scotland who became eligible by age for any of the pre-school immunisations of interest from January 2019 up to and including the week beginning 28th September 2020 were included. Of secondary interest, and to allow descriptive comparisons with data from

England, we also analysed uptake at approximately 6 months (range 24-32 weeks) for the 6in1 and 16 months for the first dose MMR. For England, equivalent data on uptake within four weeks were not available therefore the analysis was conducted on uptake by six (6in1) or 18 months of age (first MMR) and monthly, rather than weekly, data were used.

Vaccine uptake was analysed in the following four time periods: 1st January to 31st December 2019 ("2019"), 1st January to week beginning 16th March 2020 ("pre-lockdown"), 23rd March to week beginning 27th July ("lockdown") and week beginning 3rd August to week beginning 28th September 2020 inclusive ("post-lockdown"). These time periods were chosen to correspond with the beginning of the first UK-wide lockdown as announced by the UK government on the 23rd March 2020 (1). The end of the lockdown period is less well-defined and varied both in approach and timescale between Scotland and England (17, 18). Broadly speaking, by the end of July 2020, there was a substantial reduction in 'lockdown' restrictions in both countries with the opening of many non-essential businesses and limited indoor meeting between households permitted, therefore a pragmatic approach was taken to define the lockdown period as 23rd March 2020 until 31st July 2020. Children were included in the time period at which they first became eligible by age. As data from England were available by month only, the pre-lockdown period included January to end of March 2020.

Data sources

The Scottish data used in this paper were extracted in January 2021 from the PHS "COVID19 wider impacts on the health care system" dashboard, which is publicly accessible at https://scotland.shinyapps.io/phs-covid-wider-impact/. The dashboard includes aggregate information on immunisation uptake, including by the geographical area in which the child is living and the corresponding Scottish Index of Multiple Deprivation (SIMD) (both assigned based on the child's postcode registered on the Scottish national vaccination call-recall

system, SIRS). The SIMD breaks Scotland into 6976 small areas of similar population size and assigns one of five SIMD quintile based on indicators of deprivation including income, education and housing, with 1 representing the most deprived areas and 5, the least. (19) We defined geographical areas by the Health and Social Care Partnership (HSCP) in which the child lives. Within Scotland there are 31 HSCPs which provide integrated health and social care to their population.

English data were extracted from the ImmForm system, for January 2019 (representing the first extract for pre-lockdown period) to September 2020 (representing the final extract for post-lockdown period), which automatically receives monthly aggregate data on vaccine uptake from 92-95% of English GP practices, provided by GP System Suppliers. These data are validated and analysed by Public Health England to check completeness, query any anomalous results, and are used to describe epidemiological trends, as well as being used directly locally by the NHS for performance management. Data were available for immunisation uptake at age six months (each dose of the 6in1immunisation) and 18 months (first MMR) only.

Statistical methods

The primary outcome examined was the change in percentage uptake, within four weeks of eligibility, of each of the immunisations of interest between baseline uptake rates in 2019 and during the lockdown period for Scotland as a whole and by geographical areas or level of deprivation. Of secondary interest were comparisons between 2019 and the other time points listed above. Broadly comparable analyses were conducted to examine the primary outcome for England as a whole. Due to differences in data collection methods and age of child at data extraction point, comparisons between Scottish and English data were descriptive only. The prespecified analysis plan is included within the supplementary materials.

To compare uptake rates between time periods, aggregate binary logistic regression modelling was conducted, using vaccination status (vaccinated vs unvaccinated) as the dependent variable, and time period as the explanatory variable.. Separate analyses were carried out for each vaccine and country. When comparing HSCP or deprivation, an additional interaction with HSPC or SIMD quintile was included in the model. Odds ratios with 95% confidence intervals were calculated for uptake in the period of interest compared to the 2019 baseline. Given the nature of the aggregated data available, no adjusting for potential confounders or effect modifiers was possible.

All analyses and generation of figures were performed using R/R Studio (4.0.3). All code is publicly available via the EAVE II GitHub page (https://github.com/EAVE-II) and data can be accessed via the PHS wider impacts dashboard https://scotland.shinyapps.io/phs-covid-wider-impact/. Aggregated English data could be made available if in agreement with information governance regulations and upon request from the authors. Further methodological details can be found in supplemental data.

Ethics and funding

Ethical approval for this specific study was not required as we have used publicly available, anonymised, aggregated data. Results have been reported using the STROBE (20) and RECORD (21) guidelines. No specific funding was received for this project, RM is funded by the Health Data Research UK BREATHE hub. ImmForm data are not publicly available, however, an agreement was accepted for England to only share the national figures and analyses outputs.

Results

Preschool immunisation uptake increased during the lockdown period in Scotland

Across Scotland, the percentage of preschool children receiving their immunisations within four weeks of becoming eligible increased during the lockdown period for all five immunisations (Fig1, Table 1, Table S1). The change in percentage uptake compared to the 2019 baseline ranged from 1.3% for the first dose 6in1 (OR 1.28, CI 1.18-1.39) to 14.3% or the second dose MMR (OR 1.8, CI 1.74-1.87) (Table 1). Across all the immunisations visits, this equated to an additional 7,508 infants/preschool children receiving their immunisations in a timely manner over the lockdown period compared to the baseline rates of 2019. Uptake rates dipped immediately before the announcement of a national lockdown in mid-March 2020 (Fig1), then peaked throughout June before starting to decrease. However, uptake remained significantly higher than 2019 during the post lockdown period (Fig 1, Table 1). Of note, prior to lockdown for both MMR doses, there was already a modest, increase in uptake compared to 2019 (Table 1).

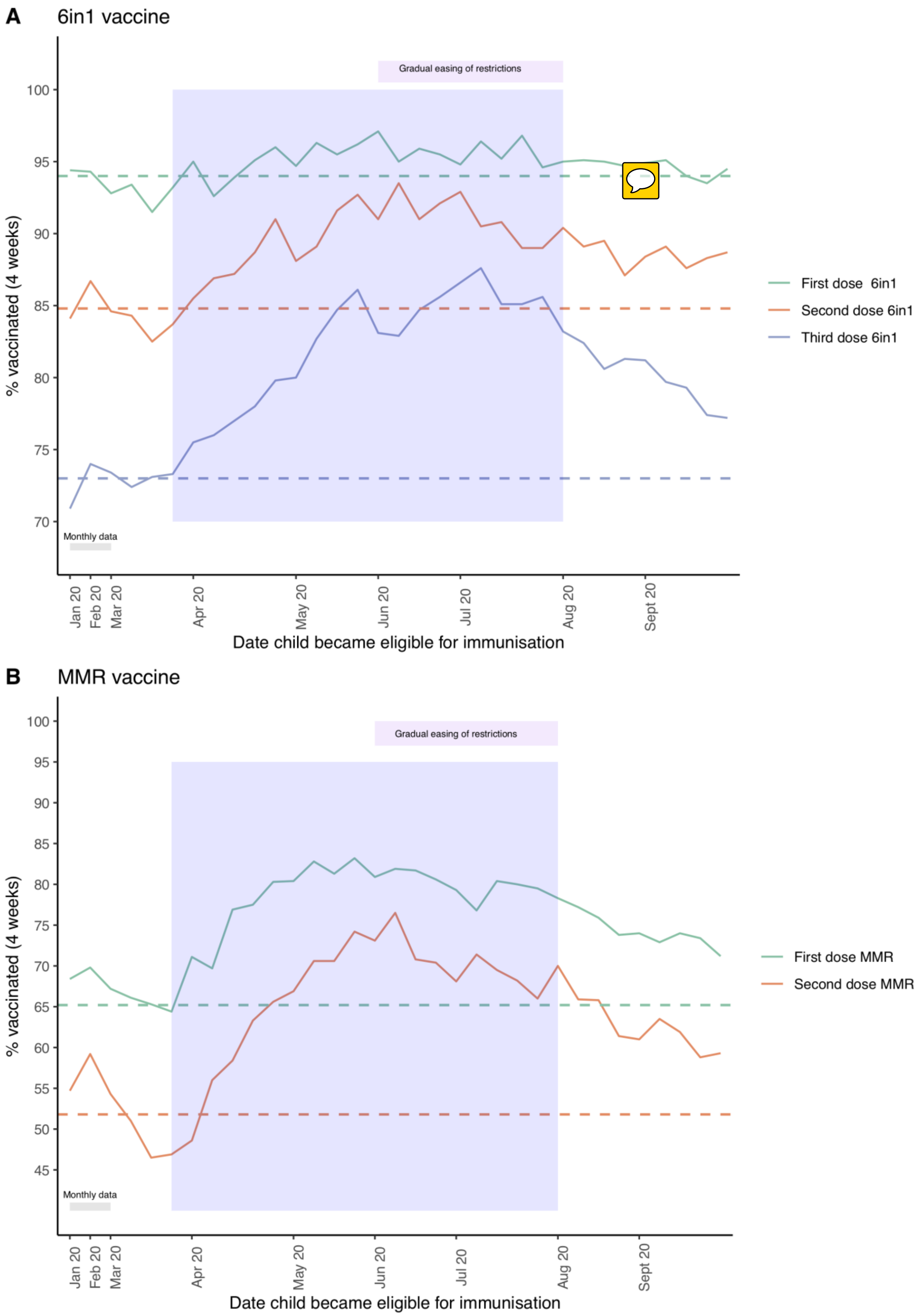

Fig 1: Percentage of children in Scotland immunised within 4 weeks of eligibility

(A) all doses 6in1 vaccine, (B) MMR vaccine across January to September 2020. Lockdown period = blue shaded area. For January and February, a single mean monthly value is plotted and from March onwards, weekly uptake is shown (see table S1 for full data). Dashed horizontal lines indicated the mean uptake in 2019 of the immunisation with the corresponding colour. The increase in uptake during the lockdown period was statistically significant (see Table 1 for details).

235 Table 1: Uptake of pre-school immunisations by time-period with odds ratio compared to baseline of 2019.

**Immunisation**	**Time period**	**% uptake within 4 weeks of eligibility (no. received/total eligible)**	**% point change from 2019**	**OR for uptake compared to 2019 (95%CI)**	***p*-value**
First6in1	2019	94 (47567/50609)	NA	NA	NA
	Pre LD	93.3 (10103/10761)	-0.7	0.98 (0.9-1.07)	0.68
	LD	95.3 (16371/17133)	1.3	1.28 (1.18-1.39)	<0.001
	Post LD	94.6 (8075/8531)	0.6	1.13 (1.02-1.25)	0.02
Second6in1	2019	84.8 (43221/50975)	0	NA	NA
	Pre LD	84.4	-0.4	1.03 (0.97-1.09)	0.39

		(9106/10698)			
	LD	89.7 (15443/17222)	4.9	1.56 (1.47-1.65)	<0.001
	Post LD	88.7 (7459/8412)	3.9	1.4 (1.31-1.51)	<0.001
Third6in1	2019	73 (37266/51083)	NA	NA	NA
	Pre LD	72.8 (8276/11394)	-0.2	0.98 (0.94-1.03)	0.49
	LD	82.1 (14039/17093)	9.1	1.7 (1.63-1.78)	<0.001
	Post LD	80.3 (6555/8172)	7.3	1.5 (1.42-1.59)	<0.001
FirstMMR	2019	65.2 (33935/52015)	NA	NA	NA
	Pre LD	67.4	2.2	1.16 (1.11-1.21)	<0.001

		(7782/11370)			
	LD	78.4 (14482/18463)	13.2	1.94 (1.86-2.02)	<0.001
	Post LD	74.5 (6740/9047)	9.3	1.56 (1.48-1.64)	<0.001
SecondMMR	2019	51.8 (25844/49940)	NA	NA	NA
	Pre LD	53.1 (6390/11495)	1.3	1.17 (1.12-1.22)	<0.001
	LD	66.1 (11303/17145)	14.3	1.8 (1.74-1.87)	<0.001
	Post LD	63.1 (5171/8196)	11.3	1.59 (1.52-1.67)	<0.001

LD = lockdown, NA = not applicable, statistically significant changes in uptake compared to 2019 are shaded green. *p*-value rounded to 2 decimal places.B

Variation in uptake of preschool immunisations by geographical area

Baseline data from 2019 showed the percentage uptake of preschool immunisations within four weeks of eligibility varied widely by geographical HSCP and immunisation (Fig2, FigS1, Table S2). In keeping with the rise in mean uptake across Scotland for all vaccines, the percentage of children immunised in most HSCP increased between 2019 and lockdown. However, not all followed this pattern with a minority demonstrating a fall in uptake (Fig2, FigS1&2, Table S2). Care must be taken when interpreting percentage results from the island HSCPs (Shetland Islands, Orkney Islands and Western Isles) given the very small numbers of children involved (Table S2).

A

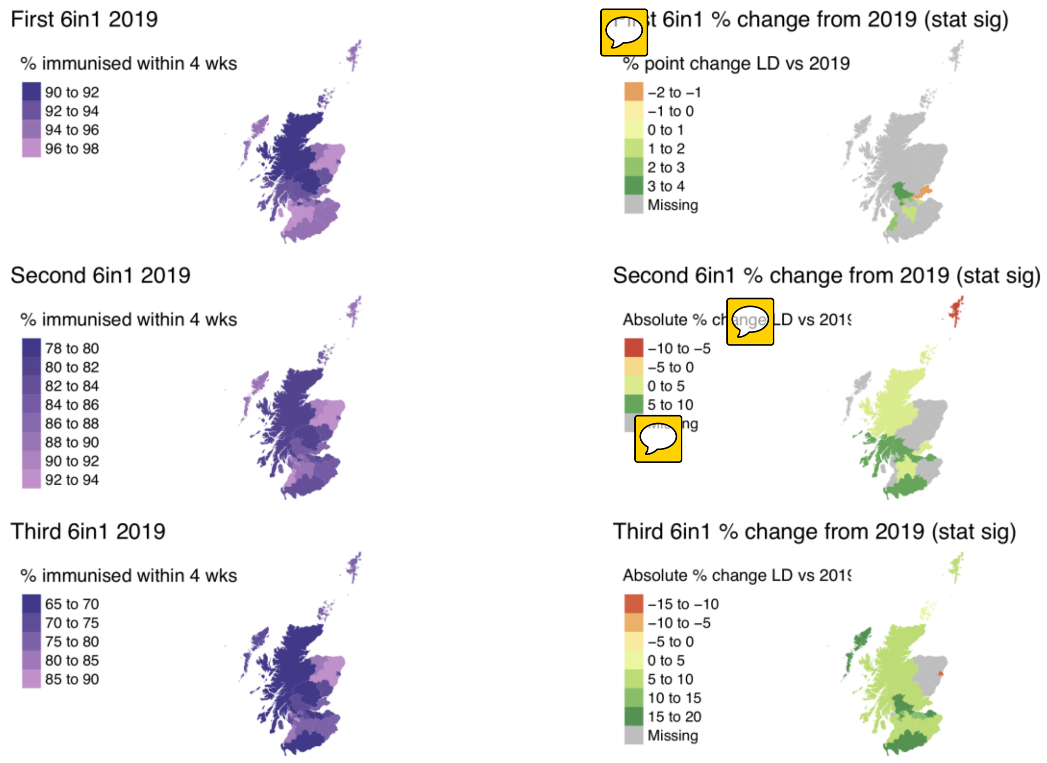

B

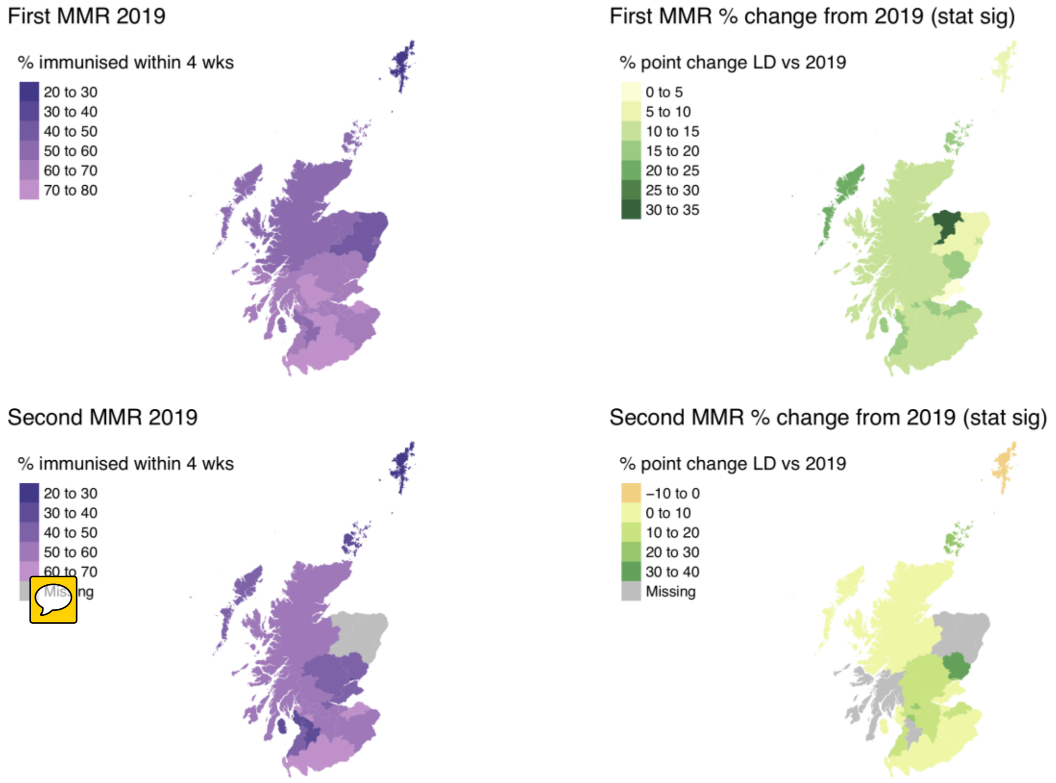

Fig 2: Choropleth maps showing baseline mean perce[illegible]ge uptake by HSCP

Left hand side = 2019, Right hand side = percentage point difference between 2019 and lockdown for areas in which the difference was statistically significant. Grey = no statistically significant changes in uptake between 2019 and lockdown, with the exception of Grampian HSCP for MMR dose 2 (see text).

For individual HSCP, the statistical significance of the change in uptake varied by immunisation (Fig 2 and Table S2). Despite a general trend of improvement for the first 6in1 vaccine, we found a significant change for only eight of the 31 HSCP, mainly centred around the more densely populated, urban central belt of Scotland (Glasgow City, Edinburgh, Stirling and Clackmannanshire, East Dunbartonshire, Falkirk, Fife, South Lanarkshire, South Ayrshire, Fig2). However, this pattern evolved with the different immunisation visits and with almost all HSCP showing a rise in uptake for both MMR immunisations, with percentage point increases as high as 30% (Angus, 74.1% vs 43.8%, OR 3.38 ,CI 2.61-4.37) (Fig 2, table S2).

Preschool immunisation uptake increased across all deprivation levels

Percentage uptake within four weeks of becoming eligible rose across all SIMD quintiles, between 2019 and lockdown, for all immunisations (Fig3). The magnitude of this rise varied by quintile and vaccine (Fig S3, Table S3) from 0.3% (SIMD 4, first 6in1 dose, OR 1.1, 95% CI 0.9-1.3) to 16.2% (SIMD 5, second MMR dose, OR 2.1, 95% CI 1.9-2.3). The increase in uptake between 2019 and lockdown was statistically significant for all except first dose 6in1 for the least deprived quintiles (4 and 5) (Table S3). In the post-lockdown period, percentage uptake remained significantly higher than the 2019 baseline for all quintiles for each vaccine except for the first 6in1 dose, in which only the most deprived quintile retained a significant increase (Fig S3, Table S3).

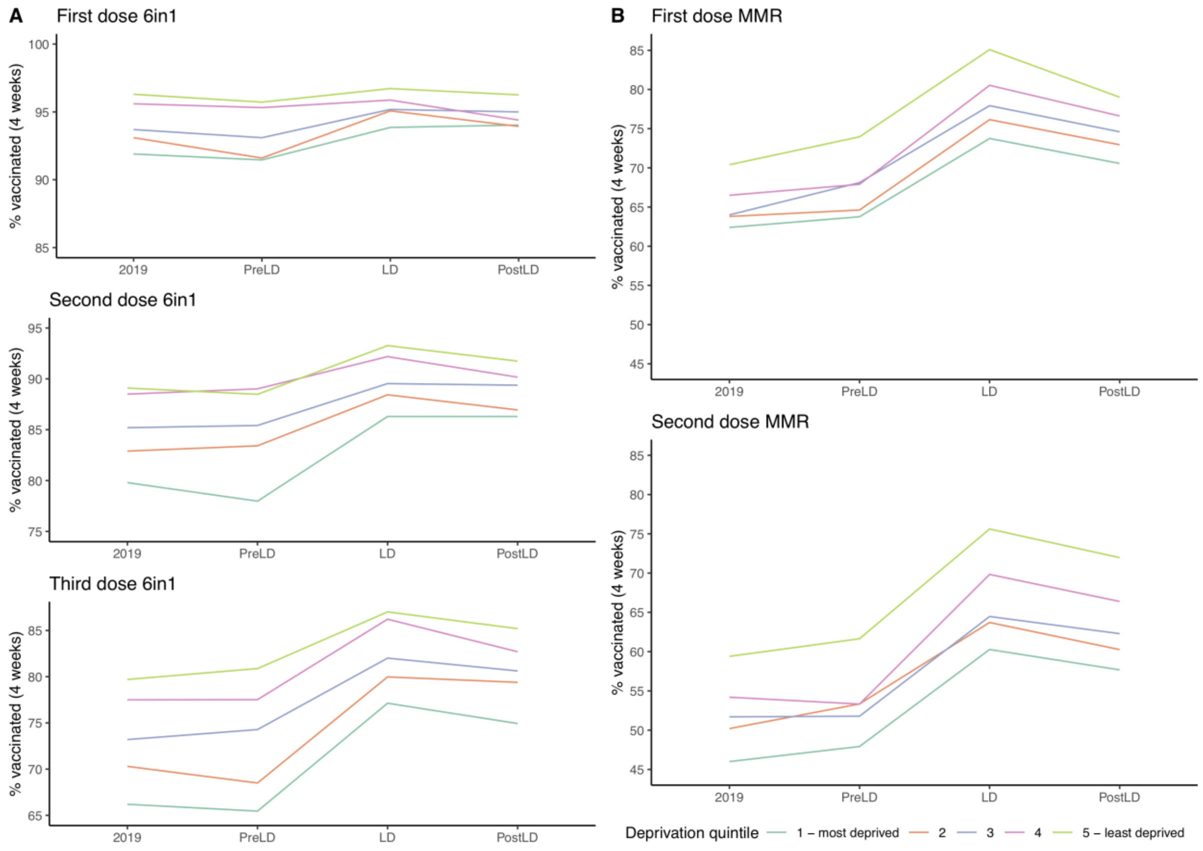

Fig 3: Mean percentage immunised by SIMD [illegible]intile. A= 6in1 vaccine, B= MMR vaccine. See Figure S3 and Table S3 for absolute percentage rise compared to 2019 and significance levels.

In keeping with previous observations (22), children in the least deprived quintiles were more likely to be immunised and this relationship was broadly maintained throughout the study period (Fig3 & FigS4). While all quintiles improved uptake between 2019 and lockdown, whether the inequality between most and least deprived increased or decreased varied by vaccine type. For all doses of the 6in1 vaccine there was a tendency to a convergent improvement i.e., the gap in percentage uptake between the quintiles lessened, while for both MMR doses there was further divergence in uptake rates between most and least deprived (Fig 3, Fig S4, Table S4). The interaction of SIMD quintile and time period was non-significant for all 6in1 doses; that is to say all SIMD quintiles improved equally for this immunisation (Table S4). However, for the first MMR dose, the improvement in uptake was

statistically greater for SIMD quintiles 3-5 compared to SIMD 1, and for the second MMR dose, SIMD 5 showed a significantly larger increase in uptake between 2019 and lockdown (Table S4). This suggests that for the MMR immunisation, the factors leading to an improvement in uptake had greater positive impact for children living in less deprived areas.

'Catch up' immunisation rates and comparison with data from England

Thus far, the Scottish data presented has related to children receiving their immunisations as per the recommended schedule (within four weeks of the child becoming eligible by age). representing a 'gold standard' in which the child is protected as early as possible. It is recognised that some children will receive their immunisations after this point. This 'catch-up' effect can be seen in uptakes rate for all Scottish children by the time they reached between six and eight months (6in1), 16 months (first MMR) or three years eight months (second MMR) (Fig 4A). Those becoming eligible during lockdown showed minimal change in this longer-term measure of uptake for the three doses of the 6in1 immunisation, a small increase in uptake of the first MMR, and a more substantial increase in uptake of the second MMR (Table S5). These data suggest that while lockdown was associated with a beneficial effect on timely uptake of all infant and pre-school immunisations, the impact on longer term or final achieved uptake was more variable, possibly reflecting a ceiling effect on maximal uptake, for the earliest immunisations.

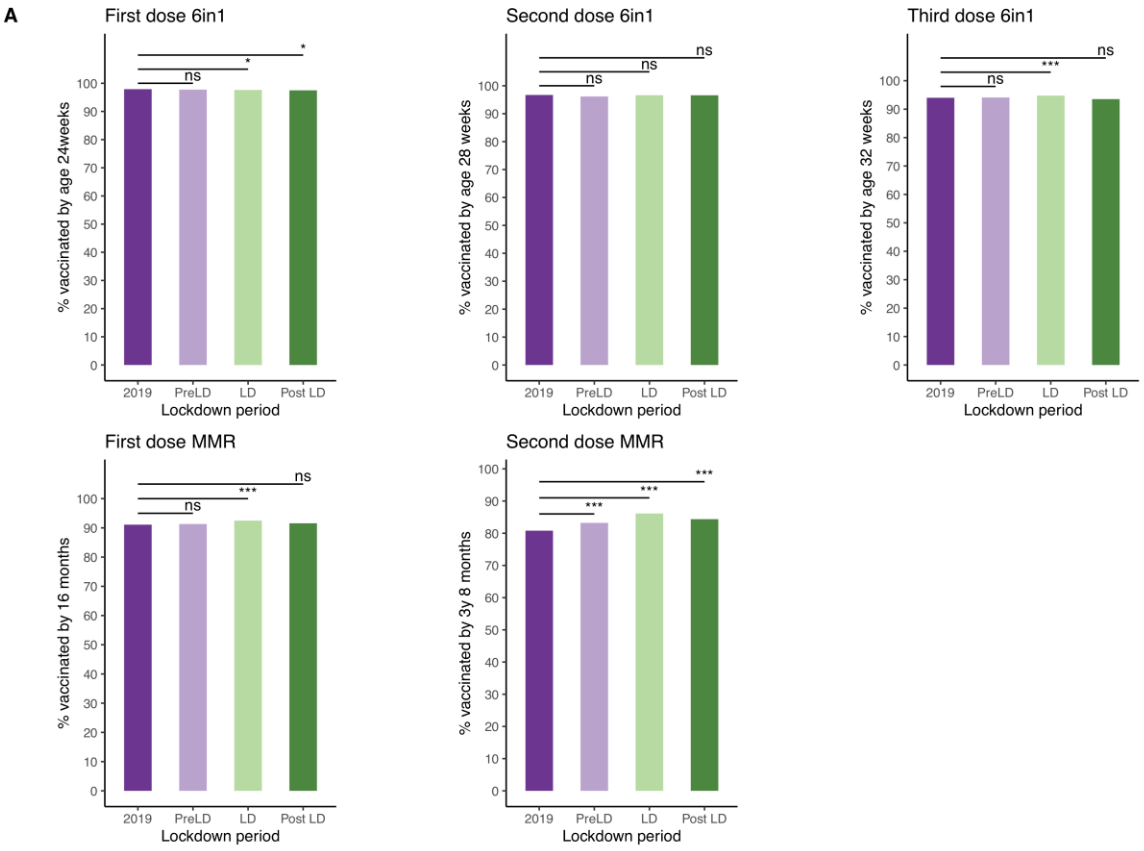

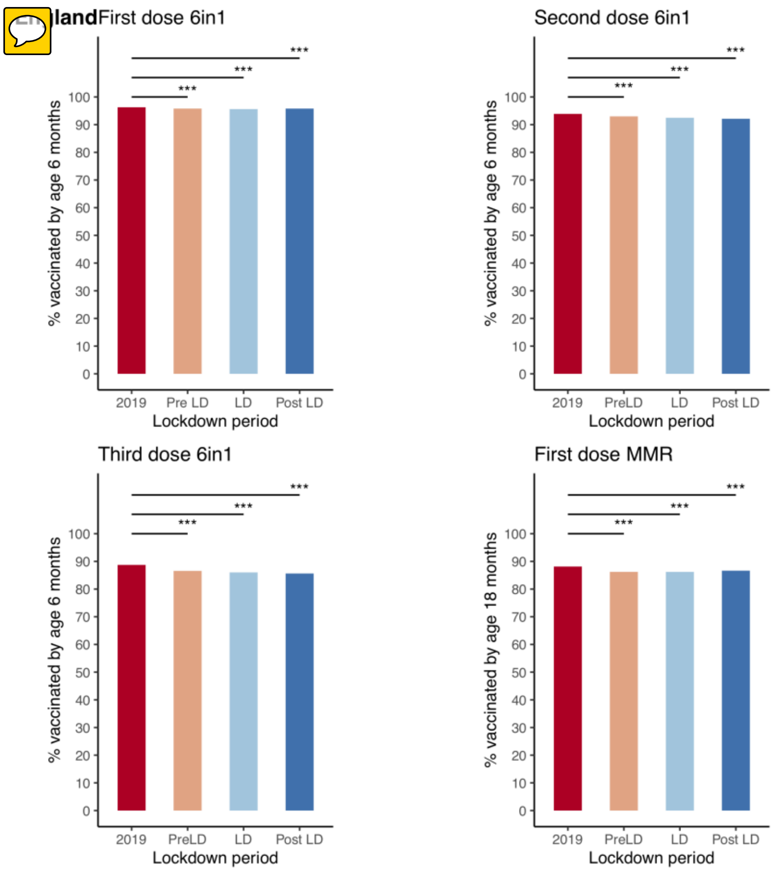

Fig 4: Percentage of children up to date at 6/18months.

Overall mean percentage of children immunised by approximately 6 months of age (all doses of 6 in1, see y-axis for specific ages) or 16-18 months (first MMR) for Scotland and England. Each bar contains children who became eligible for the immunisation of interest during the time period indicated. A= Scotland, B = England *** *p*-value < 0.001, * *p*-value <0.05, ns = not significant

For England, broadly equivalent data were available for children aged six months (all doses 6in1) and 18 months (first dose MMR) who had become eligible for their immunisations during the time periods of interest (Fig 4B, Table S6). These data demonstrated a small, but statistically significant fall in uptake for all the immunisations in the lockdown periods compared to 2019, ranging from 0.5% (first dose 6in1, 95.8% vs 96.3%, OR 0.89, CI 0.86-0.91) to 2.1% (third dose 6in1, 86.6% vs 88.7, OR 0.82, CI 0.81-0.83) (Table S6). However, much of the fall in uptake took place in the pre-lockdown period, particularly for the third dose 6in1, with a gradual recovery seen over the lockdown period itself (Fig S5). A general trend towards falling MMR uptake can also be seen to pre-date the COVID-19 pandemic (Fig S5).

Discussion

Contrary to initial reports which focused only on the very early weeks of national lockdowns implemented in response to the COVID-19 pandemic, we found that early uptake of infant and preschool immunisations (within four weeks of a child becoming eligible) rose significantly for the duration of the first lockdown period in Scotland, resulting in thousands more children receiving their immunisations at the scheduled time. This is an important message to send to support public and professional confidence in the preschool immunisation programme and will help normalise timely immunisation uptake for both parents and health services. Improving public and professional confidence is particularly vital given in the current climate of promoting vaccination against SARS-CoV-2. Encouragingly, positive results were seen across all levels of deprivation, though some geographical variations were observed across Scotland. Improvement was also seen in longer term uptake of the first and second MMR immunisations (immunisation within four months of becoming eligible) in the lockdown period. Findings in England differed, with a small fall in longer term uptake of immunisations observed for the lockdown period.

Strengths and limitations

It is important to acknowledge the limitations of the data presented, many of which arise from opportunistically using routinely collected data rather than that obtained from a specific study design. The SIRS electronic system is well-established and captures data on the entire child population in Scotland. However, the aggregate surveillance data derived from the system that we could access lacked detailed information on several potentially important factors, not least of which was ethnicity, which is known to affect both immunisation uptake and attitudes towards immunisation. (23) In the 2011 Scottish Census, 92% of the population of Scotland identified themselves as White Scottish/British, and only 4% as non-white, whereas in

England, 81% described themselves as White British and 14% non-white. (24) It may not be appropriate to extrapolate these data to countries with a significantly different ethnic make-up and it is plausible that some of the difference seen between the Scottish and English data could be due to these factors.

In using the mean percentage of the entire year 2019 as our baseline comparator, we potentially run the risk of confusing normal seasonal variation in immunisation uptake with the impact of lockdown measures. Ideally, direct weekly or monthly comparisons would be made between 2019 and 2020, however quarterly trends published for previous years including 2019 do not show major difference in uptake throughout the year and in fact show a gradual decline in uptake year on year since 2015. (25) In addition, it is possible that 2020 uptake rates have been underestimated, due to a lag in data entry into SIRS which would particularly effect the 'catch-up' rates (Fig4A).

Therefore, caution must be taken not to over-interpret the results presented here or extrapolate to significant different populations with varying baseline immunisation uptakes rates and less robust immunisation programmes, the organisation of which may have been adversely affected by the pandemic. Nevertheless, this study has efficiently and quickly produced useful and valid results which have the potential to aid the development of future research and guide policy.

Interpretation and implications for policy, practice and research

Despite these encouraging data, it is not possible to ascertain from the numbers alone which are the key contributing factors to improving uptake. This is a key avenue of future research as lessons learnt can then be taken forward to optimise future immunisation programmes, both within the pandemic setting and beyond. While the concept of vaccine hesitancy is a popular media topic, previous studies have shown that the reasons given by parents for not

vaccinating their children are often much more practical. In fact, a pre-pandemic report by the Royal Society for Public Health in the UK found that the major barriers for parents who wanted to immunise their children were timing and availability of appointments and childcare duties. (26) Although for those who actively chose not to immunise their children, fear of side effects was a key concern and the negative effects of social media are increasing. (26) The lockdown has had a major effect on parental working patterns with almost 9 million UK employees furloughed and millions more working from home, often with the additional tasks of managing childcare as schools and nurseries were closed. (27) While this had made life significantly more challenging for many, more flexible working patterns may have made attending immunisation appointments easier for some.

Jarchow-MacDonald and colleagues (28) from NHS Lothian (which consists of HSCPs Edinburgh, West Lothian, East Lothian and Midlothian) have suggested that ensuring the accessibility of immunisation centres, either by public transport or by providing mobile services to shielding families, was important in maintaining uptake during the pandemic, as was directly communicating with families with a pre-appointment phone call and reminder postcards. This gave families an opportunity to discuss the immunisation with a healthcare professional, a strategy that has been showed to be important in addressing parental concerns. (29) In fact, the reminder alone may have been sufficient to encourage parents to attend the first appointment. (30) The clear commitment of the Scottish Government to maintain the immunisation programme was also felt to be an important factor. (28)

In England, Bell et al (31) conducted a large scale online survey of parents of children under the age of 18 months to assess their experiences of accessing immunisations during the early part of the UK lockdown (19th April- 11th May 2020). They highlighted the uncertainty of some families about whether the immunisation service was continuing, particularly amongst non-white ethnic groups. While most felt it was important that their child was immunised on

time, Bell et al reported that many parents felt the risk of catching a vaccine-preventable infectious disease was reduced due to limited mixing with others. This suggests that an enhanced appreciation of the utility of immunisation was not a major motivator for parents to ensure they attended the immunisation appointments. These issues may well be reflected in the fall in immunisation uptake in England during the earlier part of lockdown reported here and a delay in receiving the first dose 6-in-1 may well led to delays in subsequent doses meaning the infants were unable to ‘catch-up’ by six months of age.

Other factors which may have had an impact on promoting timely immunisation uptake could include a reduction in fever, cough and colds which may otherwise have caused parents to delay immunisations. Though specific data on this point are lacking, a significant fall in the detection of Rhinovirus in adults was observed in 2020 compared to 2019 (32) suggests that ‘normal’ childhood respiratory infection are likely to have decreased. More work is required to fully dissect the key factors in improving timely preschool immunisation uptake in Scotland. Clarity and publicity about the continuation of the immunisation programmes, telephone reminders and the opportunity to discuss with healthcare professions seem likely to have had the most impact.

Conclusions

The ongoing COVID-19 pandemic continues to stretch health services and adversely affect all areas of life, with children disproportionately bearing the burden of the indirect consequences of pandemic control measures such as the closure of schools and limited social contact. However, opportunities have also been created in terms of enhanced surveillance of health programmes. In this study, we have used such data to investigate the effect of the pandemic on infant and preschool immunisation uptake. We have demonstrated that a robust child immunisation service can continue to deliver high and even increasing uptake rates.

Families will respond despite the many difficulties they face, to ensure that children continue to be protected again vaccine-preventable diseases. The challenge now is to use and expand on this knowledge to promote future vaccination programmes, including those targeting SARS-CoV-2.

Acknowledgments

We thank Public Health Scotland for making the data publicly available and acknowledge the support of the HDR UK BREATHE Hub and EAVE II collaborators.

References

1. Prime minister's statement on coronavirus (COVID 19): 23 March 2020 [press release]. 2020. Availale from https://www.gov.uk/government/speeches/pm-address-to-the-nation-on-coronavirus-23-march-2020, last accessed 25th May 2021

2. Scottish Government. Re-mobilise, Reciver, Re-design: th framework for NHS Scotland. 2020. Available from https://www.gov.scot/publications/re-mobilise-recover-re-design-framework-nhs-scotland/, last accessed 25th May 2021

3. Mulholland RH, Wood R, Stagg HR, et al. Impact of COVID-19 on accident and emergency attendances and emergency and planned hospital admissions in Scotland: an interrupted time-series analysis. J R Soc Med. 2020;113(11):444-53.

4. Joint Committee on Vaccination and Immunisation. JCVI statement on immunisation prioritisation 2020. Available from: https://www.gov.uk/government/publications/jcvi-statement-on-immunisation-prioritisation/statement-from-jcvi-on-immunisation-prioritisation, last accessed 25th May 2021

5. Götzinger F, Santiago-García B, Noguera-Julián A, et al. COVID-19 in children and adolescents in Europe: a multinational, multicentre cohort study. Lancet Child Adolesc Health. 2020;4(9):653-61.

6. Viner RM, Mytton OT, Bonell C, et al. Susceptibility to SARS-CoV-2 Infection Among Children and Adolescents Compared With Adults: A Systematic Review and Meta-analysis. JAMA Pediatr. 2020.

7. Araújo LA, Veloso CF, Souza MC, Azevedo JMC, Tarro G. The potential impact of the COVID-19 pandemic on child growth and development: a systematic review. J Pediatr (Rio J). 2020.

8. RCPCH workforce team. The impact of COVID - 19 on child health services - report. 7th May 2020. Avilable from https://www.rcpch.ac.uk/sites/default/files/managed-

pdf/Impact%20-of-COVID-19-child-health-services-web.pdf.pdf, last accessed 25th May 2021.

9. Saxena S, Skirrow H, Bedford H. Routine vaccination during covid-19 pandemic response. BMJ. 2020;369:m2392.

10. World Health Organization, WHO and UNICEF warn of a decline in vaccinations during COVID-19 [press release 15 July 2020]. Geneva/New York. Available from https://www.who.int/news/item/15-07-2020-who-and-unicef-warn-of-a-decline-in-vaccinations-during-covid-19, last accessed 25th May 2021.

11. McDonald HI, Tessier E, White JM, et al. Early impact of the coronavirus disease (COVID-19) pandemic and physical distancing measures on routine childhood vaccinations in England, January to April 2020. Euro Surveill. 2020;25(19).

12. Chandir S, Siddiqi DA, Mehmood M, et al. Impact of COVID-19 pandemic response on uptake of routine immunizations in Sindh, Pakistan: An analysis of provincial electronic immunization registry data. Vaccine. 2020;38(45):7146-55.

13. Jensen C, McKerrow NH. Child health services during a COVID-19 outbreak in KwaZulu-Natal Province, South Africa. S Afr Med J. 2020;0(0):13185.

14. Zhong Y, Clapham HE, Aishworiya R, et al. Childhood vaccinations: Hidden impact of COVID-19 on children in Singapore. Vaccine. 2021;39(5):780-5.

15. Santoli JM, Lindley MC, DeSilva MB, et al. Effects of the COVID-19 Pandemic on Routine Pediatric Vaccine Ordering and Administration - United States, 2020. MMWR Morb Mortal Wkly Rep. 2020;69(19):591-3.

16. Department of Health. Immunisation against infectious disease: The Green book. Public Health England; 2021.

17. PM annouces easing of lockdown restrictions: 23 June 2020 [press release]. Available from https://www.gov.uk/government/news/pm-announces-easing-of-lockdown-restrictions-23-june-2020, last accessed 25th May 2021

18. Scottish Government. Scotland's routemap through and out of the crisis. 2020. Available from https://www.gov.scot/publications/coronavirus-covid-19-framework-decision-making-scotlands-route-map-through-out-crisis/pages/2/2020. last accessed 25th May 2020

19. Scottish Government . Introducing the Scottish Index of Multiple Deprivation 2020. National statistics publication; 2020.

20. von Elm E, Altman DG, Egger M, et al. The Strengthening the Reporting of Observational Studies in Epidemiology (STROBE) statement: guidelines for reporting observational studies. J Clin Epidemiol. 2008;61(4):344-9.

21. Benchimol EI, Smeeth L, Guttmann A, et al. The REporting of studies Conducted using Observational Routinely-collected health Data (RECORD) statement. PLoS Med. 2015;12(10):e1001885.

22. Haider EA, Willocks LJ, Anderson N. Identifying inequalities in childhood immunisation uptake and timeliness in southeast Scotland, 2008-2018: A retrospective cohort study. Vaccine. 2019;37(37):5614-24.

23. Forster AS, Rockliffe L, Chorley AJ, et al. Ethnicity-specific factors influencing childhood immunisation decisions among Black and Asian Minority Ethnic groups in the UK: a systematic review of qualitative research. J Epidemiol Community Health. 2017;71(6):544-9.

24. National Office of Statistics, National Records of Scotland 2011 census aggregate data Scotland NRo, Northern, 2016. Available from http://dx.doi.org/10.5257/census/aggregate-2011-1, last accessed 25th May 2021

25. Public Health Scotland Data and Intelligence. Childhood Immunisation Statistics Scotland - Quarter ending 30 September 2019. 2019. Available from https://www.isdscotland.org/Health-Topics/Child-Health/publications/data-tables2017.asp?id=2574#2574, last accessed 25th May 2021

26. Royal Society for Public Health. Moving the needle: Promoting vaccination uptake across the life course. 2019. Available from https://www.rsph.org.uk/static/uploaded/3b82db00-a7ef-494c-85451e78ce18a779.pdf, last accessed 25th May 2021

27. HM revenus and Customs. Coronavirus Job Retention Scheme Official Statistics. 2020. Available from https://assets.publishing.service.gov.uk/government/uploads/system/uploads/attachment_data/file/891249/Coronavirus_Job_Retention_Scheme_Statistics_June_2020.pdf, last accessed 25th May 2021

28. Jarchow-MacDonald AA, Burns R, Miller J, Kerr L, Willocks LJ. Keeping childhood immunisation rates stable during the COVID-19 pandemic. Lancet Infect Dis. 2021.

29. Campbell H, Edwards A, Letley L, Bedford H, Ramsay M, Yarwood J. Changing attitudes to childhood immunisation in English parents. Vaccine. 2017;35(22):2979-85.

30. Jacobson Vann JC, Szilagyi P. Patient reminder and patient recall systems to improve immunization rates. Cochrane Database Syst Rev. 2005(3):CD003941.

31. Bell S, Clarke R, Paterson P, Mounier-Jack S. Parents' and guardians' views and experiences of accessing routine childhood vaccinations during the coronavirus (COVID-19) pandemic: A mixed methods study in England. PLoS One. 2020;15(12):e0244049.

32. Poole S, Brendish NJ, Tanner AR, Clark TW. Physical distancing in schools for SARS-CoV-2 and the resurgence of rhinovirus. Lancet Respir Med. 2020;8(12):e92-e3.

Supporting information captions

S1 Fig: Percentage uptake by HSCP for 2019 (pale orange) and lockdown (dark orange) with HSCP ordered by uptake for 2019 (note: this varies by immunisation). Dashed horizontal lines indicated the mean uptake for all of Scotland for the time period of the corresponding colour. A = 6in1 immunisation, B = MMR.

S2 Fig: Choropleth maps showing all (significant or not) percentage point changes between 2019 and lockdown for each HSCP for all immunisations.

S3 Fig: Absolute percentage change in uptake compared to 2019 for each immunisations and SIMD for each time period (A = pre lockdown, B= lockdown, C= post lockdown). Significance rates varied by immunisation and SIMD, for details see table S3.

S4 Fig: Combined odds ratio plot with 95% confidence intervals comparing each SIMD quintile (2-5) to SIMD 1-most deprived for 2019 (dark blue) and LD (light blue).

S5 Fig: Percentage of children in England immunised by 6 months of age (first, second and third dose 6in1) or 18 months of age (first dose MMR) from January 2019 to September 2020. The start and end of the lockdown period is indicated by the dashed blue lines.

S1 Table: Percentage uptake of each immunisation by year (2019), month (Jan and Feb 2020) or week as per data availability. W/B = week beginning.

S2 Tables A-E: Percentage uptake, percent point change in uptake compared to 2019 and significance level for this change for each HSCP at each time-period. Each table shows results for a different immunisation. Results were considered significant if *p*-value <0.05 and 95% CI did not include 1. Statistically significant p values are shaded green and significant results for the 2019-LD comparisons are plotted on Figure 2. HSCP = Health and Social Care

Partnership, OR = odds ratio, CI = confidence interval, NA = not applicable, PreLD = pre lockdown, LD = lockdown, PostLD = post lockdown. *p*-value rounded to 2 decimal places.

S3 Table: Uptake of pre-school immunisations by time period and SIMD and percent point change in uptake compared to baseline 2019. Odds ratio and 95% confidence intervals shown are for change in uptake compared to 2019. *p*-value rounded to 2 decimal places. LD = lockdown, NA = not applicable. Statistically significant change in uptake compared to 2019 are shaded green.

S4 Table: To assess whether the differences between change in uptake were statistically significant between SIMD quintiles, the interaction between time period and SIMD quintile was added into the model. The baseline comparisons showed are for time period 2019 and deprivation quintile SIMD 1. ROR = ratio of odds ratio, calculated by taking the exponential function of the coefficient of the interaction term from the interaction model. If the 95% confidence intervals did not include 1, the interaction of time period and SIMD was considered statistically significant, that is; there was a significant difference in the level of change (2019- time period) between the deprivation quintile and SIMD 1. For example, the increase in uptake during lockdown for SIMD 5 was statistically greater than the increase in uptake for SIMD 1. The ROR can be used to calculate the odds ratio for uptake compared to the baseline levels by multiplying the ROR with the relevant OR in table S3. LD= lockdown, SIMD = Scottish Index of Multiple Deprivation, ns = not statistically significant (coloured green) = interaction was statistically significant. *p*-value rounded to 2 decimal places.

S5 Table: Scotland. Uptake of pre-school immunisations at an older age by time period and point percentage change from 2019 with odds ratio and 95% confidence intervals compared to baseline of 2019. Children are categorised into the time-period at which they became eligible for the immunisation as before and uptake data were extracted at a later stage when

they reached the ages indicated in the immunisation column. LD = lockdown, NA = not applicable. Statistically significant changes are coloured green.

S6 Table: England. Uptake of pre-school immunisations at an older age by time period and point percentage change from 2019 with odds ratio and 95% confidence intervals compared to baseline of 2019. Children are categorised into the time-period at which they became eligible for the immunisation as before and uptake data were extracted at a later stage when they reached the ages indicated in the immunisation column. LD = lockdown, NA = not applicable. Statistically significant changes are coloured green.

S1 File: Childhood immunisation V1.0 Final analysis plan

S2 File: Supplemental methods

S3 File: RECORD plus STROBE checklist

S1 Fig

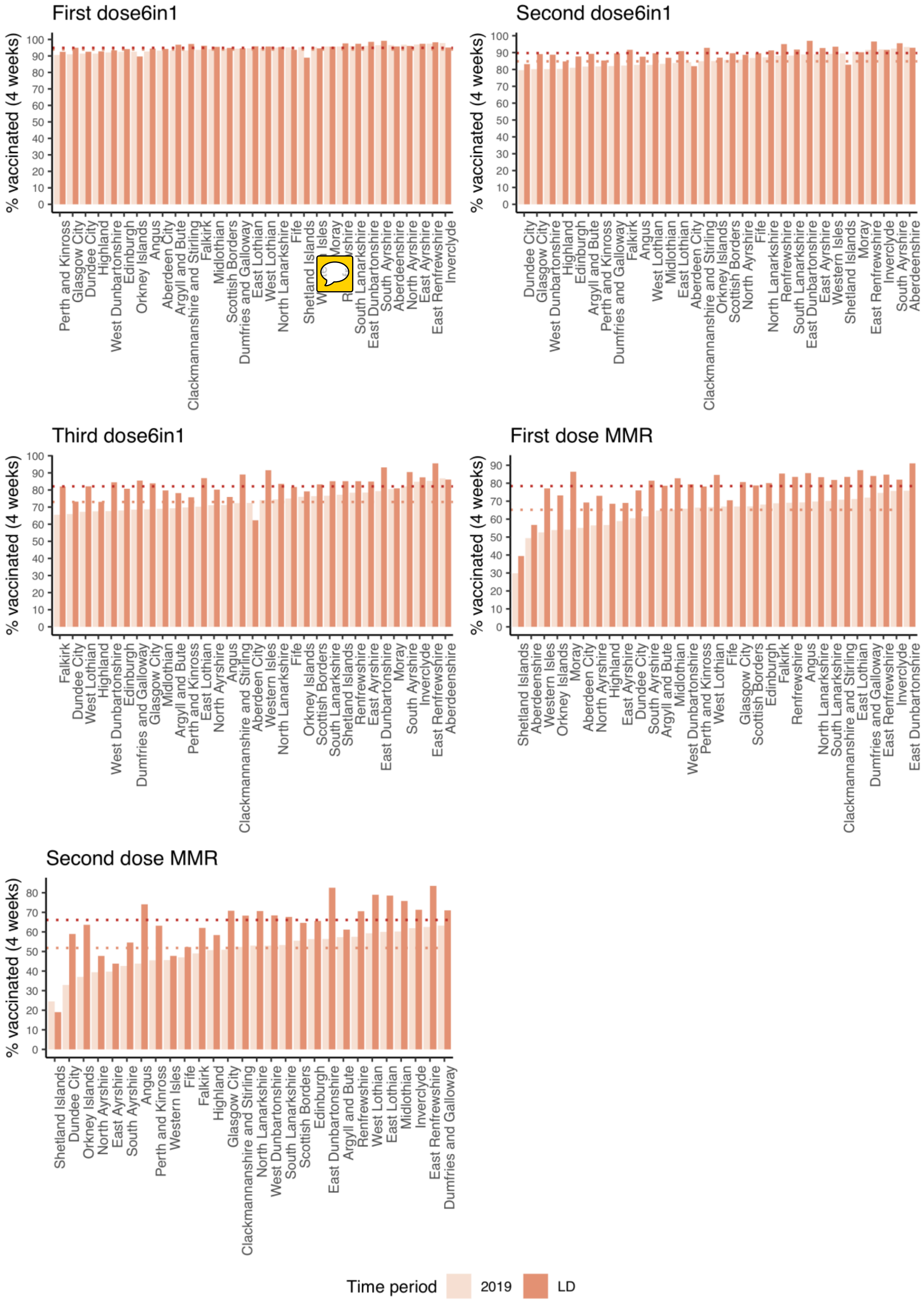

First dose6in1
Second dose6in1
Third dose6in1
First dose MMR
Second dose MMR
% vaccinated (4 weeks)
Time period
2019
LD

S2 Fig

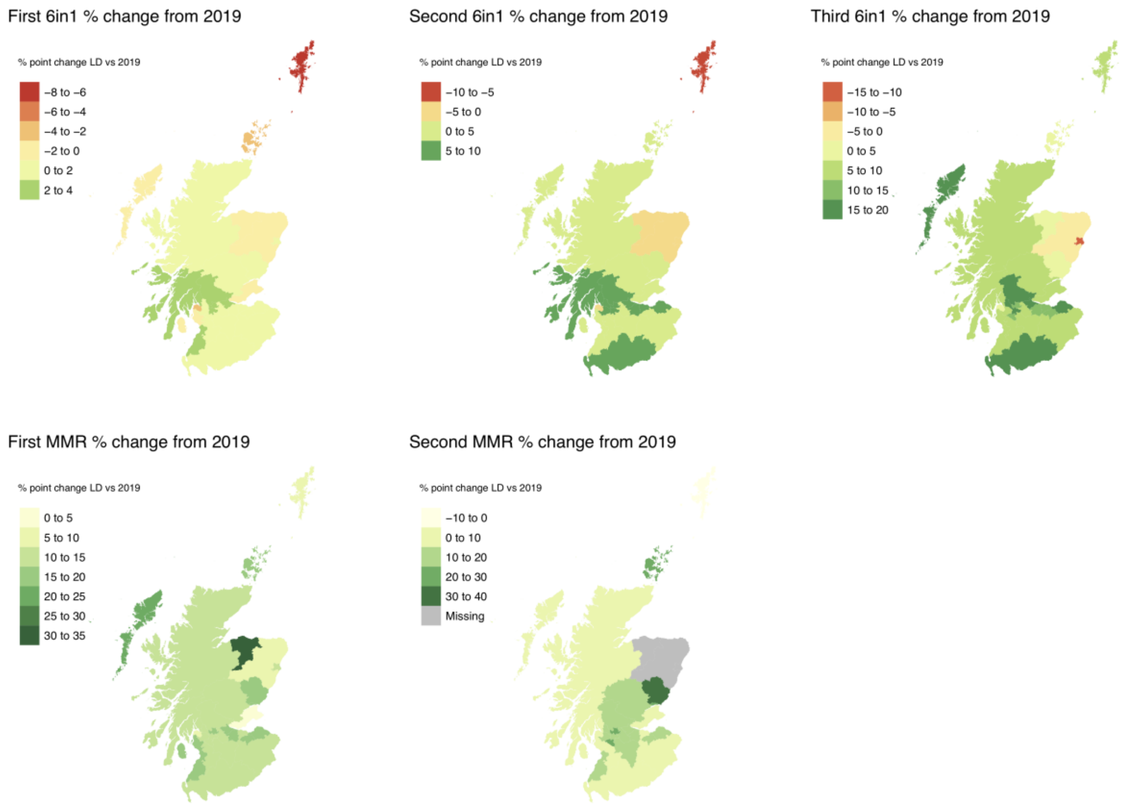
First 6in1 % change from 2019
% point change LD vs 2019
−8 to −6
−6 to −4
−4 to −2
−2 to 0
0 to 2
2 to 4
Second 6in1 % change from 2019
% point change LD vs 2019
−10 to −5
−5 to 0
0 to 5
5 to 10
Third 6in1 % change from 2019
% point change LD vs 2019
−15 to −10
−10 to −5
−5 to 0
0 to 5
5 to 10
10 to 15
15 to 20
First MMR % change from 2019
% point change LD vs 2019
0 to 5
5 to 10
10 to 15
15 to 20
20 to 25
25 to 30
30 to 35
Second MMR % change from 2019
% point change LD vs 2019
−10 to 0
0 to 10
10 to 20
20 to 30
30 to 40
Missing

S3 Fig

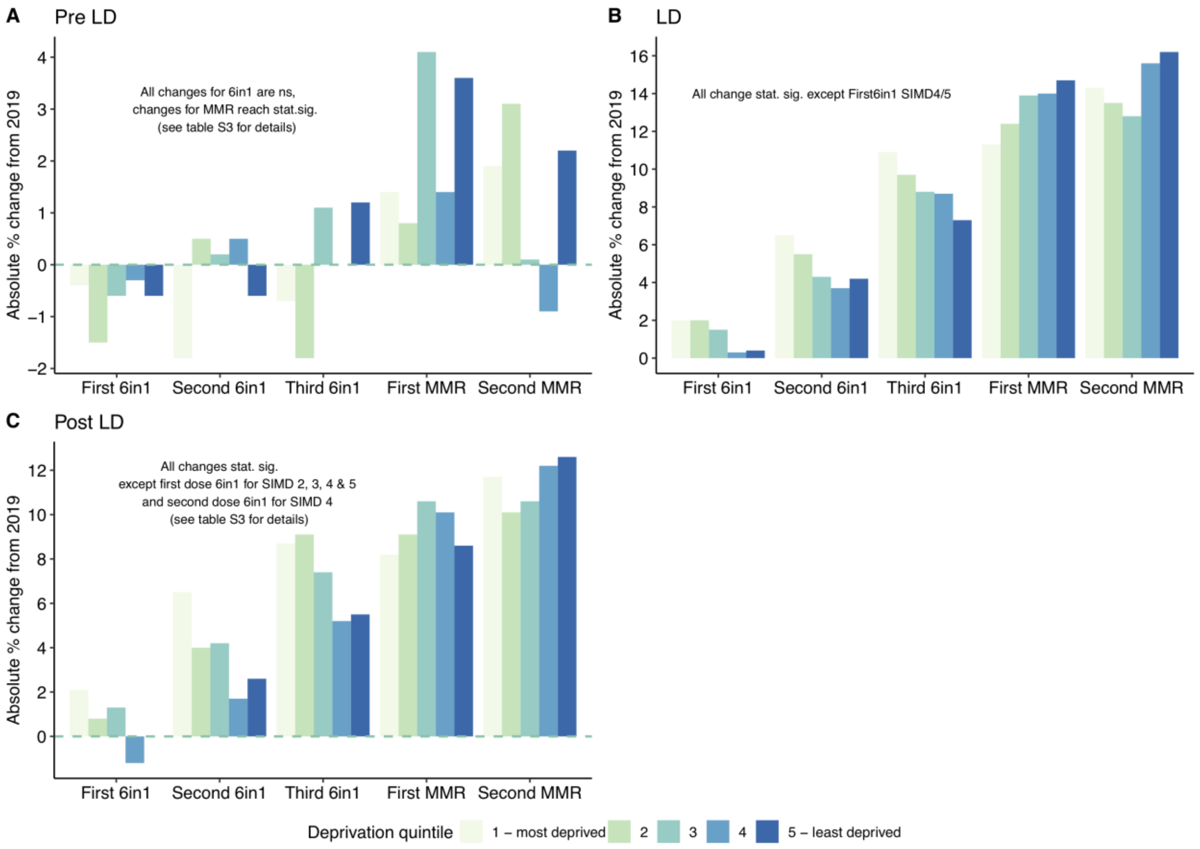
A
Pre LD
All changes for 6in1 are ns,
changes for MMR reach stat.sig.
(see table S3 for details)
Absolute % change from 2019
B
LD
All change stat. sig. except First6in1 SIMD4/5
C
Post LD
All changes stat. sig.
except first dose 6in1 for SIMD 2, 3, 4 & 5
and second dose 6in1 for SIMD 4
(see table S3 for details)
First 6in1
Second 6in1
Third 6in1
First MMR
Second MMR
Deprivation quintile
1 – most deprived
2
3
4
5 – least deprived

S4 Fig

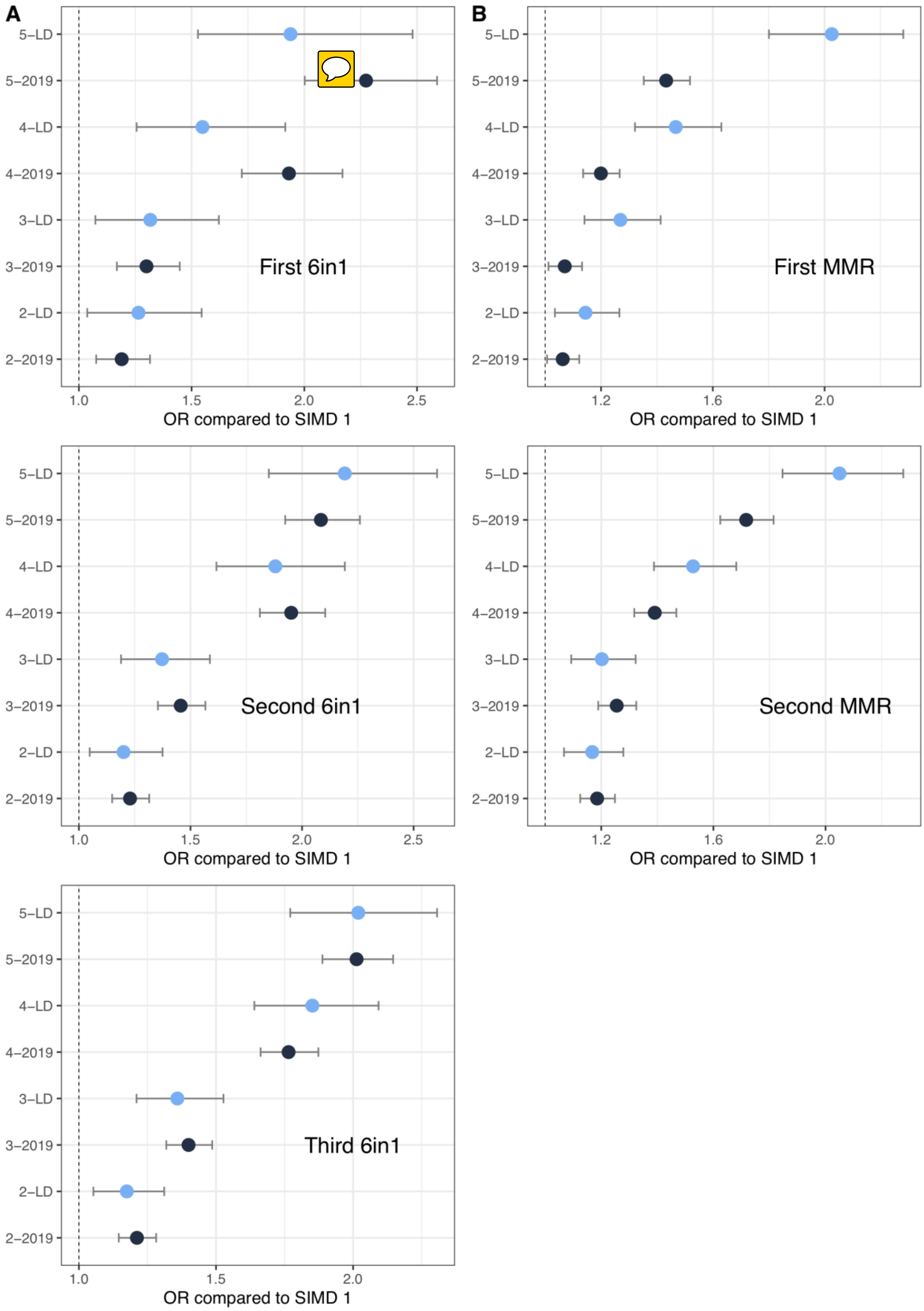
A
B
First 6in1
First MMR
Second 6in1
Second MMR
Third 6in1
5–LD
5–2019
4–LD
4–2019
3–LD
3–2019
2–LD
2–2019
1.0
1.5
2.0
2.5
1.2
1.6
OR compared to SIMD 1

S5 Fig

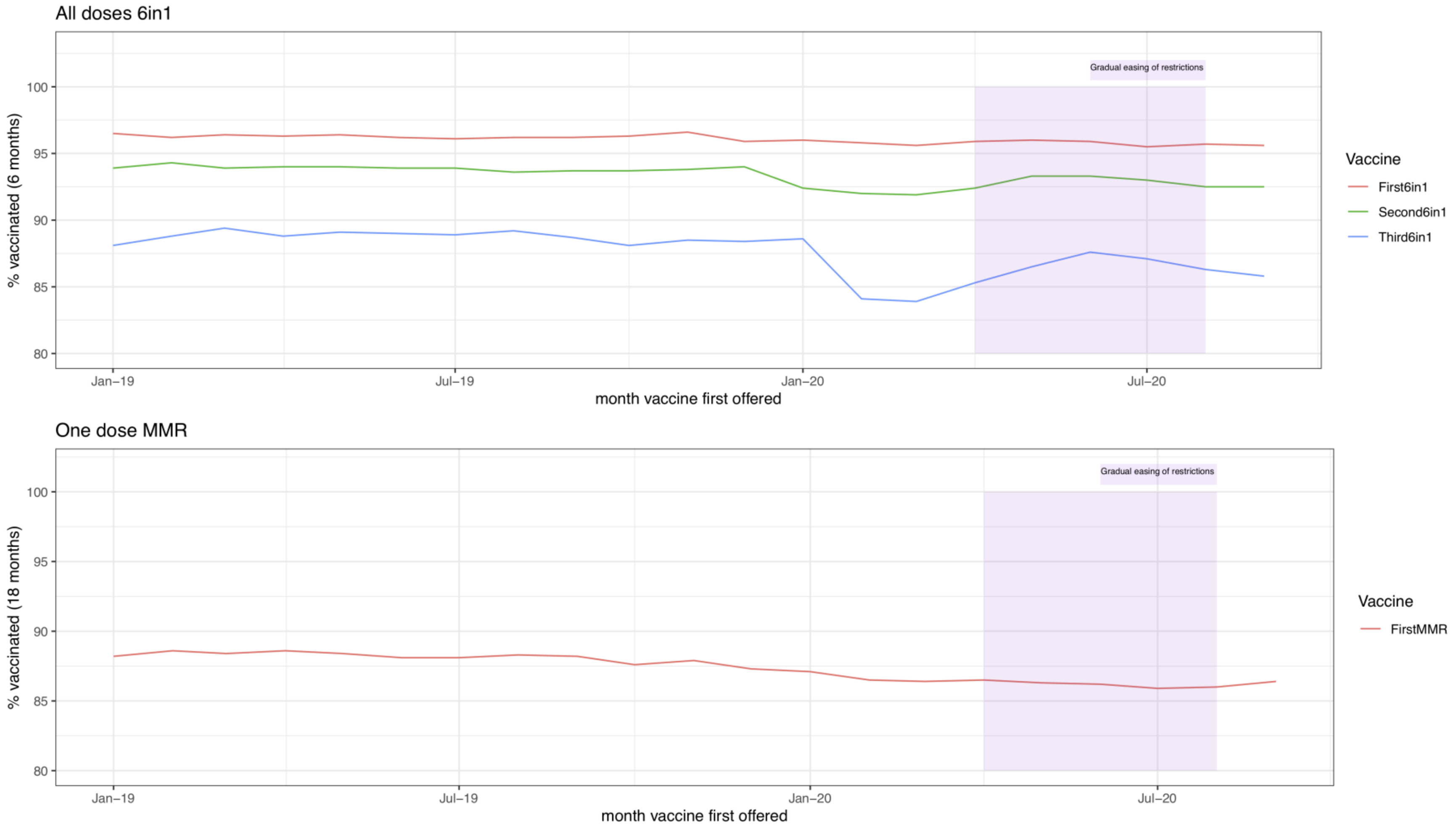
All doses 6in1
Gradual easing of restrictions
% vaccinated (6 months)
100
95
90
85
80
Jan–19
Jul–19
Jan–20
Jul–20
month vaccine first offered
Vaccine
First6in1
Second6in1
Third6in1
One dose MMR
Gradual easing of restrictions
% vaccinated (18 months)
100
95
90
85
80
Jan–19
Jul–19
Jan–20
Jul–20
month vaccine first offered
Vaccine
FirstMMR

Tables S1-6

Supplementary Table S1

% uptake within 4 weeks of eligibility (number received/total eligible)					
**Time period**	**First 6in1**	**Second 6in1**	**Third 6in1**	**First MMR**	**Second MMR**
2019	94 (47567/50609)	84.8 (43221/50975)	73 (37266/51083)	65.2 (33935/52015)	51.8 (25844/49940)
Jan-20	94.4 (3393/3593)	84.1 (3101/3689)	70.9 (2916/4112)	68.4 (2578/3767)	54.7 (2151/3934)
Feb-20	94.3 (4079/4325)	86.7 (3878/4472)	74 (3403/4600)	69.8 (3309/4739)	59.2 (2845/4804)
W/B 02-MAR-20	92.8 (813/876)	84.6 (729/862)	73.4 (628/856)	67.2 (636/947)	54.3 (491/904)
W/B 09-MAR-20	93.4 (883/945)	84.3 (763/905)	72.4 (624/862)	66.1 (656/993)	51 (474/930)
W/B 16-MAR-20	91.5 (935/1022)	82.5 (635/770)	73.1 (705/964)	65.3 (603/924)	46.5 (429/923)
W/B 23-MAR-20	93.2 (846/908)	83.7 (761/909)	73.3 (644/879)	64.4 (588/913)	46.9 (430/916)
W/B 30-MAR-20	95 (864/909)	85.5 (749/876)	75.5 (651/862)	71.1 (692/973)	48.6 (472/972)
W/B 06-APR-20	92.6 (892/963)	86.9 (821/945)	76 (688/905)	69.7 (636/912)	56 (509/909)
W/B 13-APR-20	93.9 (835/889)	87.2 (891/1022)	77 (593/770)	76.9 (749/974)	58.4 (558/955)
W/B 20-APR-20	95.1 (851/895)	88.7 (805/908)	78 (709/909)	77.5 (732/945)	63.3 (567/896)
W/B 27-APR-20	96 (881/918)	91 (827/909)	79.8 (699/876)	80.3 (789/982)	65.6 (563/858)
W/B 04-MAY-20	94.7 (838/885)	88.1 (848/963)	80 (756/945)	80.4 (781/971)	66.9 (590/882)
W/B 11-MAY-20	96.3 (894/928)	89.1 (792/889)	82.7 (845/1022)	82.8 (815/984)	70.6 (653/925)
W/B 18-MAY-20	95.5 (857/897)	91.6 (820/895)	84.7 (769/908)	81.3 (784/964)	70.6 (602/853)
W/B 25-MAY-20	96.2 (840/873)	92.7 (851/918)	86.1 (783/909)	83.2 (820/986)	74.2 (636/857)
W/B 01-JUN-20	97.1 (835/860)	91 (805/885)	83.1 (800/963)	80.9 (728/900)	73.1 602/824)
W/B 08-JUN-20	95 (899/946)	93.5 (868/928)	82.9 (737/889)	81.9 (801/978)	76.5 (739/966)
W/B 15-JUN-20	95.9	91	84.7	81.7	70.8

	(880/918)	(816/897)	(758/895)	(793/971)	(644/909)
W/B 22-JUN-20	95.5 (804/842)	92.1 (804/873)	85.6 (786/918)	80.6 (789/979)	70.4 (650/923)
W/B 29-JUN-20	94.8 (795/839)	92.9 (799/860)	86.6 (766/885)	79.3 (783/987)	68.1 (608/893)
W/B 06-JUL-20	96.4 (863/895)	90.5 (856/946)	87.6 (813/928)	76.8 (788/1026)	71.4 (637/892)
W/B 13-JUL-20	95.2 (894/939)	90.8 (834/918)	85.1 (763/897)	80.4 (805/1001)	69.5 (629/905)
W/B 20-JUL-20	96.8 (864/893)	89 (749/842)	85.1 (743/873)	80 (813/1016)	68.2 (597/875)
W/B 27-JUL-20	94.6 (885/936)	89 (747/839)	85.6 (736/860)	79.5 (796/1001)	66 (617/935)
W/B 03-AUG-20	95 (916/964)	90.4 (809/895)	83.2 (787/946)	78.3 (808/1032)	70 (656/937)
W/B 10-AUG-20	95.1 (847/891)	89.1 (837/939)	82.4 (756/918)	77.2 (761/986)	65.9 (602/913)
W/B 17-AUG-20	95 (914/962)	89.5 (799/893)	80.6 (679/842)	75.9 (749/987)	65.8 (571/868)
W/B 24-AUG-20	94.7 (946/999)	87.1 (815/936)	81.3 (682/839)	73.8 (721/977)	61.4 (551/897)
W/B 31-AUG-20	94.9 (885/933)	88.4 (852/964)	81.2 (727/895)	74 (709/958)	61 (540/885)
W/B 07-SEP-20	95.1 (939/987)	89.1 (794/891)	79.7 (748/939)	72.9 (725/995)	63.5 (589/927)
W/B 14-SEP-20	94 (885/941)	87.6 (843/962)	79.3 (708/893)	74 (751/1015)	61.9 (569/919)
W/B 21-SEP-20	93.5 (864/924)	88.3 (882/999)	77.4 (724/936)	73.4 (782/1066)	58.8 (549/933)
W/B 28-SEP-20	94.5 (879/930)	88.7 (828/933)	77.2 (744/964)	71.2 (734/1031)	59.3 (544/917)

Supplementary Tables S2

A First dose 6in1

HSCP	Time period	% uptake (within 4 weeks)	Number received	Number eligible	% point change from 2019	OR compared to 2019 (95% CI)	p value
Aberdeen City	2019	93.2	2077	2228	NA	NA	NA
	PreLD	90.5	382	425	-2.7	0.65 (0.45-0.92)	0.02
	LD	94.2	696	739	1	1.18 (0.83-1.67)	0.36
	PostLD	91.6	334	365	-1.6	0.78 (0.52-1.17)	0.24
Aberdeenshire	2019	96.6	2452	2538	NA	NA	NA
	PreLD	95.8	513	540	-0.8	0.67 (0.43-1.04)	0.07
	LD	95.7	793	830	-0.9	0.75 (0.51-1.11)	0.16
	PostLD	92.1	354	385	-4.5	0.4 (0.26-0.61)	<0.001
Angus	2019	92.7	939	1013	NA	NA	NA
	PreLD	92.4	205	219	-0.3	1.15 (0.64-2.08)	0.63
	LD	94.5	312	330	1.8	1.37 (0.8-2.32)	0.25
	PostLD	93.9	158	169	1.2	1.13 (0.59-2.18)	0.71
Argyll and Bute	2019	93.3	608	652	NA	NA	NA
	PreLD	93.4	140	150	0.1	1.01 (0.5-2.06)	0.97
	LD	96.8	234	243	3.5	1.88 (0.9-3.92)	0.09
	PostLD	91.3	96	106	-2	0.69 (0.34-1.43)	0.32
Clackmannanshire and Stirling	2019	93.3	1092	1170	NA	NA	NA
	PreLD	92.9	272	288	-0.4	1.21 (0.7-2.11)	0.49
	LD	97.3	398	408	4	2.84 (1.46-5.55)	<0.001

	PostLD	95.9	191	199	2.6	1.71 (0.81-3.59)	0.16
Dumfries and Galloway	2019	94.4	1116	1182	NA	NA	NA
	PreLD	91.1	231	247	-3.3	0.85 (0.49-1.5)	0.58
	LD	94.6	404	429	0.2	0.96 (0.59-1.54)	0.85
	PostLD	93.7	194	206	-0.7	0.96 (0.51-1.8)	0.89
Dundee City	2019	91.4	1289	1411	NA	NA	NA
	PreLD	89.1	266	290	-2.3	1.05 (0.66-1.66)	0.84
	LD	92.5	426	461	1.1	1.15 (0.78-1.7)	0.48
	PostLD	92.1	206	225	0.7	1.03 (0.62-1.7)	0.92
East Ayrshire	2019	96.9	1122	1158	NA	NA	NA
	PreLD	95.1	241	252	-1.8	0.7 (0.35-1.4)	0.32
	LD	97.4	420	431	0.5	1.23 (0.62-2.43)	0.56
	PostLD	94.1	181	193	-2.8	0.48 (0.25-0.95)	0.03
East Dunbartonshire	2019	96.1	958	997	NA	NA	NA
	PreLD	95.7	187	193	-0.4	1.27 (0.53-3.04)	0.59
	LD	98.6	339	344	2.5	2.76 (1.08-7.06)	0.03
	PostLD	99.4	154	155	3.3	6.27 (0.86-45.96)	0.07
East Lothian	2019	94.6	1031	1090	NA	NA	NA
	PreLD	93.9	194	204	-0.7	1.11 (0.56-2.21)	0.77
	LD	95.8	330	346	1.2	1.18 (0.67-2.08)	0.57
	PostLD	95.3	194	203	0.7	1.23 (0.6-2.53)	0.57
East Renfrewshire	2019	97.4	868	891	NA	NA	NA
	PreLD	96	193	201	-1.4	0.64 (0.28-1.45)	0.28
	LD	98.3	290	295	0.9	1.54	0.39

						(0.58-4.08)	
	PostLD	97.4	149	153	0	0.99 (0.34-2.89)	0.98
Edinburgh	2019	92.5	4130	4464	NA	NA	NA
	PreLD	89.9	883	964	-2.6	0.88 (0.68-1.14)	0.33
	LD	94.2	1517	1613	1.7	1.28 (1.01-1.62)	0.04
	PostLD	94.4	751	796	1.9	1.35 (0.98-1.86)	0.07
Falkirk	2019	93.7	1382	1475	NA	NA	NA
	PreLD	95	291	308	1.3	1.15 (0.68-1.96)	0.6
	LD	96.2	513	534	2.5	1.64 (1.01-2.67)	0.04
	PostLD	95.8	245	256	2.1	1.5 (0.79-2.84)	0.21
Fife	2019	95.2	3234	3396	NA	NA	NA
	PreLD	93.4	700	744	-1.8	0.8 (0.57-1.12)	0.19
	LD	93.7	1078	1150	-1.5	0.75 (0.56-1)	0.05
	PostLD	92.3	520	562	-2.9	0.62 (0.44-0.88)	0.01
Glasgow City	2019	91	5855	6435	NA	NA	NA
	PreLD	92.5	1254	1346	1.5	1.35 (1.07-1.7)	0.01
	LD	95	2029	2136	4	1.88 (1.52-2.32)	<0.001
	PostLD	94.8	1069	1128	3.8	1.79 (1.36-2.36)	<0.001
Highland	2019	91.4	1821	1992	NA	NA	NA
	PreLD	88.8	389	433	-2.6	0.83 (0.59-1.18)	0.3
	LD	92.8	587	635	1.4	1.15 (0.82-1.6)	0.42
	PostLD	92.9	336	360	1.5	1.31 (0.84-2.05)	0.23
Inverclyde	2019	97.8	618	632	NA	NA	NA
	PreLD	96.9	146	151	-0.9	0.66 (0.23-1.87)	0.43

	LD	95.1	217	227	-2.7	0.49 (0.22-1.12)	0.09
	PostLD	97.1	96	99	-0.7	0.72 (0.2-2.57)	0.62
Midlothian	2019	94.1	1051	1117	NA	NA	NA
	PreLD	94.4	212	226	0.3	0.95 (0.52-1.72)	0.87
	LD	95.5	336	352	1.4	1.32 (0.75-2.31)	0.33
	PostLD	95.8	191	200	1.7	1.33 (0.65-2.72)	0.43
Moray	2019	95.7	788	823	NA	NA	NA
	PreLD	95.2	209	222	-0.5	0.71 (0.37-1.37)	0.31
	LD	95.6	295	310	-0.1	0.87 (0.47-1.62)	0.67
	PostLD	97.4	141	145	1.7	1.57 (0.55-4.47)	0.4
North Ayrshire	2019	96.8	1082	1118	NA	NA	NA
	PreLD	98.2	261	270	1.4	0.96 (0.46-2.03)	0.92
	LD	96	371	386	-0.8	0.82 (0.45-1.52)	0.53
	PostLD	93.7	182	193	-3.1	0.55 (0.28-1.1)	0.09
North Lanarkshire	2019	95.1	3337	3510	NA	NA	NA
	PreLD	95.6	696	724	0.5	1.29 (0.86-1.94)	0.22
	LD	95.5	1049	1098	0.4	1.11 (0.8-1.54)	0.53
	PostLD	97.1	609	627	2	1.75 (1.07-2.87)	0.03
Orkney Islands	2019	92.6	189	204	NA	NA	NA
	PreLD	100	28	28	7.4	1.16 (0.55-2.44)	0.69
	LD	89.7	53	59	-2.9	1.27 (0.81-2.01)	0.3
	PostLD	100	37	37	7.4	0.8 (0.48-1.33)	0.39
Perth and Kinross	2019	90.6	1124	1240	NA	NA	NA
	PreLD	87.4	225	255	-3.2	0.77	0.24

						(0.51-1.19)	
	LD	92.5	411	444	1.9	1.29 (0.86-1.92)	0.22
	PostLD	93.2	191	206	2.6	1.31 (0.75-2.3)	0.34
Renfrewshire	2019	95.7	1617	1689	NA	NA	NA
	PreLD	97.8	377	385	2.1	2.1 (1-4.39)	0.05
	LD	97.6	587	602	1.9	1.74 (0.99-3.06)	0.05
	PostLD	96.6	239	248	0.9	1.18 (0.58-2.4)	0.64
Scottish Borders	2019	94.3	838	889	NA	NA	NA
	PreLD	93.9	183	193	-0.4	1.11 (0.56-2.23)	0.76
	LD	94.9	291	306	0.6	1.18 (0.65-2.13)	0.58
	PostLD	95.8	142	148	1.5	1.44 (0.61-3.42)	0.41
Shetland Islands	2019	95.3	203	213	NA	NA	NA
	PreLD	86.4	22	25	-8.9	0.36 (0.09-1.41)	0.14
	LD	89	54	60	-6.3	1.27 (0.81-2.01)	0.3
	PostLD	89.6	33	37	-5.8	0.8 (0.48-1.33)	0.39
South Ayrshire	2019	96.5	844	875	NA	NA	NA
	PreLD	94.1	187	198	-2.4	0.62 (0.31-1.26)	0.19
	LD	99.2	274	277	2.7	3.35 (1.02-11.06)	0.05
	PostLD	97.4	148	152	0.9	1.36 (0.47-3.91)	0.57
South Lanarkshire	2019	96	3066	3194	NA	NA	NA
	PreLD	94.6	622	646	-1.4	1.08 (0.69-1.69)	0.73
	LD	97.4	1084	1113	1.4	1.56 (1.04-2.35)	0.03
	PostLD	96.4	484	502	0.4	1.12 (0.68-1.86)	0.65
West Dunbartonshire	2019	92.1	796	864	NA	NA	NA

	PreLD	90.3	158	172	-1.8	0.96 (0.53-1.76)	0.9
	LD	93.4	274	292	1.3	1.3 (0.76-2.23)	0.34
	PostLD	95.8	144	150	3.7	2.05 (0.87-4.81)	0.1
West Lothian	2019	94.7	1754	1852	NA	NA	NA
	PreLD	93.9	342	363	-0.8	0.91 (0.56-1.48)	0.7
	LD	95.7	547	571	1	1.27 (0.81-2.01)	0.3
	PostLD	93.4	272	291	-1.3	0.8 (0.48-1.33)	0.39
Western Isles	2019	95.4	188	197	NA	NA	NA
	PreLD	95.8	33	35	0.4	0.79 (0.16-3.82)	0.77
	LD	94.6	56	59	-0.8	1.18 (0.83-1.67)	0.36
	PostLD	95.4	23	24	0	0.78 (0.52-1.17)	0.24

B Second dose 6in1

HSCP	Time period	% uptake (within 4 weeks)	Number received	Number eligible	% point change from 2019	OR compared to 2019 (95% CI)	p value
Aberdeen City	2019	84.3	1891	2244	NA	NA	NA
	PreLD	74.7	358	459	-9.6	0.66 (0.52-0.85)	<0.001
	LD	81.9	603	733	-2.4	0.87 (0.69-1.08)	0.2
	PostLD	82	281	344	-2.3	0.83 (0.62-1.12)	0.23
Aberdeenshire	2019	93.3	2411	2584	NA	NA	NA
	PreLD	87.4	470	530	-5.9	0.56 (0.41-0.77)	<0.001
	LD	92.9	753	811	-0.4	0.93 (0.68-1.27)	0.65
	PostLD	90.7	365	404	-2.6	0.67 (0.47-0.97)	0.03
Angus	2019	82.7	843	1019	NA	NA	NA
	PreLD	80	188	223	-2.7	1.12 (0.75-1.67)	0.57
	LD	87.6	288	330	4.9	1.43 (1-2.06)	0.05
	PostLD	85.7	136	160	3	1.18 (0.74-1.88)	0.48
Argyll and Bute	2019	81.7	523	640	NA	NA	NA
	PreLD	84.4	137	167	2.7	1.02 (0.66-1.59)	0.92
	LD	89.3	214	240	7.6	1.84 (1.17-2.9)	0.01
	PostLD	83.8	86	107	2.1	0.92 (0.55-1.54)	0.74
Clackmannanshire and Stirling	2019	84.9	1003	1182	NA	NA	NA
	PreLD	91.2	231	259	6.3	(1.47 (0.96-2.25)	0.07
	LD	92.8	416	448	7.9	2.32 (1.57-3.44)	<0.001
	PostLD	92.5	173	188	7.6	2.06 (1.19-3.57)	0.01
Dumfries and Galloway	2019	82	978	1193	NA	NA	NA

	PreLD	87.2	217	251	5.2	1.4<br>(0.95-2.07)	0.09
	LD	89.5	363	409	7.5	1.73<br>(1.23-2.44)	<0.001
	PostLD	90.3	197	218	8.3	2.06<br>(1.28-3.31)	<0.001
Dundee City	2019	79.6	1133	1423	NA	NA	NA
	PreLD	75.9	226	284	-3.7	1<br>(0.73-1.37)	0.99
	LD	83.2	385	463	3.6	1.26<br>(0.96-1.66)	0.1
	PostLD	83.9	199	238	4.3	1.31<br>(0.91-1.88)	0.15
East Ayrshire	2019	89	1052	1182	NA	NA	NA
	PreLD	88.2	214	242	-0.8	0.94<br>(0.61-1.46)	0.8
	LD	92.7	400	429	3.7	1.7<br>(1.12-2.59)	0.01
	PostLD	92.8	187	202	3.8	1.54<br>(0.88-2.69)	0.13
East Dunbartonshire	2019	88.9	893	1005	NA	NA	NA
	PreLD	96.4	186	196	7.5	2.33<br>(1.2-4.54)	0.01
	LD	97	331	342	8.1	3.77<br>(2.01-7.1)	<0.001
	PostLD	95.4	168	176	6.5	2.63<br>(1.26-5.5)	0.01
East Lothian	2019	83.8	932	1112	NA	NA	NA
	PreLD	87.1	169	198	3.3	1.13<br>(0.74-1.72)	0.59
	LD	90.9	326	360	7.1	1.85<br>(1.26-2.73)	<0.001
	PostLD	88.8	152	170	5	1.63<br>(0.98-2.73)	0.06
East Renfrewshire	2019	91.6	834	910	NA	NA	NA
	PreLD	95.8	171	181	4.2	1.56<br>(0.79-3.07)	0.2
	LD	96.6	282	292	5	2.57<br>(1.31-5.04)	0.01
	PostLD	96.3	156	162	4.7	2.37<br>(1.01-5.54)	0.05

Edinburgh	2019	81	3650	4505	NA	NA	NA
	PreLD	83.3	814	980	2.3	1.15<br>(0.96-1.38)	0.14
	LD	87.7	1387	1582	6.7	1.67<br>(1.41-1.97)	<0.001
	PostLD	83.9	679	810	2.9	1.21<br>(0.99-1.48)	0.06
Falkirk	2019	82.3	1243	1511	NA	NA	NA
	PreLD	82.8	260	315	0.5	1.02<br>(0.74-1.4)	0.91
	LD	91.7	473	517	9.4	2.32<br>(1.66-3.24)	<0.001
	PostLD	92.1	231	250	9.8	2.62<br>(1.61-4.26)	<0.001
Fife	2019	86.8	2966	3418	NA	NA	NA
	PreLD	83.7	627	732	-3.1	0.91<br>(0.72-1.14)	0.42
	LD	89.6	1030	1151	2.8	1.3<br>(1.05-1.6)	0.02
	PostLD	90.2	509	562	3.4	1.46<br>(1.08-1.98)	0.01
Glasgow City	2019	80.2	5196	6480	NA	NA	NA
	PreLD	83.2	1116	1347	3	1.19<br>(1.02-1.39)	0.02
	LD	89.1	1894	2125	8.9	2.03<br>(1.74-2.35)	<0.001
	PostLD	90.8	971	1069	10.6	2.45<br>(1.97-3.04)	<0.001
Highland	2019	80.3	1600	1992	NA	NA	NA
	PreLD	75.5	332	419	-4.8	0.93<br>(0.72-1.21)	0.61
	LD	84.8	565	669	4.5	1.33<br>(1.05-1.69)	0.02
	PostLD	80.1	258	321	-0.2	1<br>(0.75-1.35)	0.98
Inverclyde	2019	91.9	575	626	NA	NA	NA
	PreLD	91.2	142	156	-0.7	0.9<br>(0.48-1.67)	0.74
	LD	91.6	207	226	-0.3	0.97<br>(0.56-1.68)	0.9
	PostLD	88.7	91	101	-3.2	0.81	0.56

						(0.4-1.65)	
Midlothian	2019	83.3	935	1122	NA	NA	NA
	PreLD	84.1	181	218	0.8	0.98<br>(0.66-1.44)	0.91
	LD	86.9	308	354	3.6	1.34<br>(0.95-1.9)	0.1
	PostLD	93.9	172	183	10.6	3.13<br>(1.67-5.87)	<0.001
Moray	2019	90.4	751	831	NA	NA	NA
	PreLD	88.8	171	197	-1.6	0.7<br>(0.44-1.12)	0.14
	LD	90.2	279	311	-0.2	0.93<br>(0.6-1.43)	0.74
	PostLD	94.2	146	155	3.8	1.73<br>(0.85-.52)	0.13
North Ayrshire	2019	85.8	952	1109	NA	NA	NA
	PreLD	82.7	221	257	-3.1	1.01<br>(0.69-1.5)	0.95
	LD	88.7	354	399	2.9	1.3<br>(0.91-1.85)	0.15
	PostLD	85.4	171	201	-0.4	0.94<br>(0.62-1.44)	0.77
North Lanarkshire	2019	87.2	3090	3542	NA	NA	NA
	PreLD	85.6	623	724	-1.6	0.9<br>(0.72-1.14)	0.39
	LD	91.3	1037	1136	4.1	1.53<br>(1.22-1.93)	<0.001
	PostLD	89.3	508	566	2.1	1.28<br>(0.96-1.71)	0.09
Orkney Islands	2019	85	175	206	NA	NA	NA
	PreLD	93.2	29	31	8.2	2.57<br>(0.58-11.32)	0.21
	LD	87	53	61	2	1.88<br>(1.39-2.53)	<0.001
	PostLD	97	26	27	12	1.48<br>(1.02-2.14)	0.04
Perth and Kinross	2019	81.8	1016	1242	NA	NA	NA
	PreLD	76.9	211	264	-4.9	0.89<br>(0.63-1.24)	0.48
	LD	85.3	382	446	3.5	1.33<br>(0.98-1.79)	0.07

	PostLD	85.5	171	200	3.7	1.31 (0.86-1.99)	0.2
Renfrewshire	2019	88.2	1508	1710	NA	NA	NA
	PreLD	90.8	334	366	2.6	1.4 (0.95-2.07)	0.09
	LD	95	593	623	6.8	2.65 (1.78-3.93)	<0.001
	PostLD	92.2	246	269	4	1.43 (0.91-2.25)	0.12
Scottish Borders	2019	85.6	740	864	NA	NA	NA
	PreLD	83.3	178	212	-2.3	0.88 (0.58-1.33)	0.53
	LD	89.6	283	315	4	1.48 (0.98-2.24)	0.06
	PostLD	86.8	134	153	1.2	1.18 (0.7-1.98)	0.53
Shetland Islands	2019	89.5	197	220	NA	NA	NA
	PreLD	83.8	21	25	-5.8	0.61 (0.19-1.94)	0.41
	LD	82.8	56	68	-6.7	1.88 (1.39-2.53)	<0.001
	PostLD	77.3	25	32	-12.2	1.48 (1.02-2.14)	0.04
South Ayrshire	2019	92.4	830	898	NA	NA	NA
	PreLD	89.5	165	184	-2.9	0.71 (0.42-1.22)	0.21
	LD	95.5	277	290	3.1	1.75 (0.95-3.21)	0.07
	PostLD	92.3	121	132	-0.1	0.9 (0.46-1.75)	0.76
South Lanarkshire	2019	88.2	2821	3199	NA	NA	NA
	PreLD	85.9	590	666	-2.3	1.04 (0.8-1.35)	0.77
	LD	91.8	1014	1104	3.6	1.51 (1.19-1.92)	<0.001
	PostLD	90.8	489	539	2.6	1.31 (0.96-1.79)	0.09
West Dunbartonshire	2019	80.2	693	864	NA	NA	NA
	PreLD	85.5	131	160	5.3	1.11 (0.72-1.72)	0.63
	LD	88.7	263	296	8.5	1.97	<0.001

						(1.32-2.93)	
	PostLD	85	128	149	4.8	1.5<br>(0.92-2.46)	0.1
West Lothian	2019	82.7	1525	1845	NA	NA	NA
	PreLD	85.3	318	368	2.6	1.33<br>(0.97-1.84)	0.08
	LD	89.6	510	567	6.9	1.88<br>(1.39-2.53)	<0.001
	PostLD	87.5	254	290	4.8	1.48<br>(1.02-2.14)	0.04
Western Isles	2019	89.2	173	194	NA	NA	NA
	PreLD	83.3	30	36	-5.9	0.61<br>(0.23-1.63)	0.32
	LD	93.5	60	64	4.3	0.87<br>(0.69-1.08)	0.2
	PostLD	88.9	24	28	-0.3	0.83<br>(0.62-1.12)	0.23

C Third dose 6in1

HSCP	Time period	% uptake (within 4 weeks)	Number received	Number eligible	% point change from 2019	OR compared to 2019 (95% CI)	p value
Aberdeen City	2019	72.4	1609	2222	NA	NA	NA
	PreLD	65.8	345	520	-6.6	0.75<br>(0.61-0.92)	0.01
	LD	62.3	450	719	-10.1	0.64<br>(0.53-0.76)	<0.001
	PostLD	75	252	336	2.6	1.14<br>(0.88-1.49)	0.32
Aberdeenshire	2019	86.8	2266	2611	NA	NA	NA
	PreLD	82	446	536	-4.8	0.75<br>(0.59-0.97)	0.03
	LD	86.1	721	838	-0.7	0.94<br>(0.75-1.18)	0.58
	PostLD	80.8	336	416	-6	0.64<br>(0.49-0.84)	<0.001

Angus	2019	71.2	738	1036	NA	NA	NA
	PreLD	76	159	214	4.8	1.17 (0.84-1.63)	0.37
	LD	75.9	261	345	4.7	1.25 (0.95-1.66)	0.11
	PostLD	79	121	154	7.8	1.48 (0.98-2.23)	0.06
Argyll and Bute	2019	69.3	443	639	NA	NA	NA
	PreLD	72.9	120	170	3.6	1.06 (0.73-1.54)	0.75
	LD	78.2	178	230	8.9	1.51 (1.07-2.15)	0.02
	PostLD	78.8	94	118	9.5	1.73 (1.07-2.8)	0.02
Clackmannanshire and Stirling	2019	72.3	860	1189	NA	NA	NA
	PreLD	74.4	217	282	2.1	1.28 (0.94-1.73)	0.12
	LD	89	384	432	16.7	3.06 (2.21-4.24)	<0.001
	PostLD	86.1	167	193	13.8	2.46 (1.59-3.79)	<0.001
Dumfries and Galloway	2019	68.5	805	1175	NA	NA	NA
	PreLD	66.4	187	280	-2.1	0.92 (0.7-1.22)	0.58
	LD	85.5	339	401	17	2.51 (1.87-3.38)	<0.001
	PostLD	81	177	221	12.5	1.85 (1.3-2.63)	<0.001
Dundee City	2019	65.9	933	1416	NA	NA	NA
	PreLD	62	213	325	-3.9	0.98 (0.76-1.27)	0.9
	LD	73.2	326	442	7.3	1.45 (1.15-1.85)	<0.001
	PostLD	72.5	170	235	6.6	1.35 (1-1.84)	0.05
East Ayrshire	2019	78.5	930	1184	NA	NA	NA
	PreLD	78.4	204	260	-0.1	0.99 (0.72-1.38)	0.98
	LD	84.9	357	420	6.4	1.55 (1.14-2.09)	<0.001
	PostLD	87	172	198	8.5	1.81	0.01

						(1.17-2.79)	
East Dunbartonshire	2019	79.3	808	1019	NA	NA	NA
	PreLD	86.3	180	213	7	1.42 (0.95-2.13)	0.08
	LD	93.2	304	326	13.9	3.61 (2.28-5.71)	<0.001
	PostLD	92.4	152	165	13.1	3.05 (1.7-5.49)	<0.001
East Lothian	2019	70.2	786	1119	NA	NA	NA
	PreLD	67.9	153	225	-2.3	0.9 (0.66-1.23)	0.5
	LD	86.9	298	345	16.7	2.69 (1.92-3.75)	<0.001
	PostLD	82.9	140	167	12.7	2.2 (1.43-3.38)	<0.001
East Renfrewshire	2019	85.3	795	932	NA	NA	NA
	PreLD	84.5	145	167	-0.8	1.14 (0.7-1.84)	0.61
	LD	95.6	294	310	10.3	3.17 (1.85-5.41)	<0.001
	PostLD	89.6	134	147	4.3	1.78 (0.98-3.23)	0.06
Edinburgh	2019	68	3058	4494	NA	NA	NA
	PreLD	68.1	721	1043	0.1	1.05 (0.91-1.22)	0.5
	LD	80.7	1253	1545	12.7	2.02 (1.75-2.32)	<0.001
	PostLD	75.8	583	771	7.8	1.46 (1.22-1.74)	<0.001
Falkirk	2019	65.5	969	1480	NA	NA	NA
	PreLD	73.7	241	337	8.2	1.32 (1.02-1.72)	0.03
	LD	82	412	503	16.5	2.39 (1.86-3.07)	<0.001
	PostLD	83.3	212	254	17.8	2.66 (1.88-3.77)	<0.001
Fife	2019	75	2571	3426	NA	NA	NA
	PreLD	73.5	556	768	-1.5	0.87 (0.73-1.04)	0.13
	LD	81.7	940	1151	6.7	1.48 (1.25-1.75)	<0.001

	PostLD	86.4	484	561	11.4	2.09 (1.62-2.69)	<0.001
Glasgow City	2019	68.7	4454	6484	NA	NA	NA
	PreLD	72.9	1011	1424	4.2	1.12 (0.98-1.27)	0.09
	LD	83.9	1796	2140	15.2	2.38 (2.1-2.7)	<0.001
	PostLD	84.4	842	998	15.7	2.46 (2.06-2.94)	<0.001
Highland	2019	67.5	1340	1984	NA	NA	NA
	PreLD	61.4	279	461	-6.1	0.74 (0.6-0.91)	<0.001
	LD	73.1	480	653	5.6	1.33 (1.09-1.62)	<0.001
	PostLD	63.9	199	311	-3.6	0.85 (0.67-1.1)	0.22
Inverclyde	2019	84.8	542	639	NA	NA	NA
	PreLD	78.6	121	151	-6.2	0.72 (0.46-1.14)	0.16
	LD	87.4	206	238	2.6	1.15 (0.75-1.77)	0.52
	PostLD	84.6	91	105	-0.2	1.16 (0.64-2.13)	0.62
Midlothian	2019	69	791	1146	NA	NA	NA
	PreLD	66.6	151	222	-2.4	0.95 (0.7-1.3)	0.77
	LD	79.7	288	360	10.7	1.8 (1.35-2.39)	<0.001
	PostLD	82.4	143	173	13.4	2.14 (1.42-3.23)	<0.001
Moray	2019	80.5	680	845	NA	NA	NA
	PreLD	66.2	129	188	-14.3	0.53 (0.37-0.75)	<0.001
	LD	80.9	259	320	0.4	1.03 (0.74-1.43)	0.86
	PostLD	86.8	134	155	6.3	1.55 (0.95-2.53)	0.08
North Ayrshire	2019	71.1	781	1099	NA	NA	NA
	PreLD	74.3	198	268	3.2	1.15 (0.85-1.56)	0.36
	LD	80.2	334	417	9.1	1.55	0.08

						(0.95-2.53)	
	PostLD	67	117	175	-4.1	0.82<br>(0.58-1.15)	0.26
North Lanarkshire	2019	74.5	2651	3558	NA	NA	NA
	PreLD	77.7	579	758	3.2	1.11<br>(0.92-1.33)	0.28
	LD	83.6	959	1148	9.1	1.74<br>(1.46-2.06)	<0.001
	PostLD	77.8	404	517	3.3	1.22<br>(0.98-1.53)	0.07
Orkney Islands	2019	76	155	204	NA	NA	NA
	PreLD	82.2	27	33	6.2	1.42<br>(0.56-3.65)	0.46
	LD	79.2	39	51	3.2	2.22<br>(1.76-2.79)	<0.001
	PostLD	94.4	28	29	18.5	1.74<br>(1.27-2.39)	<0.001
Perth and Kinross	2019	69.8	847	1213	NA	NA	NA
	PreLD	57.3	204	313	-12.5	0.81<br>(0.62-1.05)	0.11
	LD	75.7	317	415	5.9	1.4<br>(1.08-1.81)	0.01
	PostLD	74.8	166	219	5	1.35<br>(0.97-1.89)	0.07
Renfrewshire	2019	78.4	1342	1712	NA	NA	NA
	PreLD	81.3	307	386	2.9	1.07<br>(0.82-1.41)	0.62
	LD	85.1	537	630	6.7	1.59<br>(1.24-2.04)	<0.001
	PostLD	84.5	228	271	6.1	1.46<br>(1.03-2.07)	0.03
Scottish Borders	2019	76.4	662	867	NA	NA	NA
	PreLD	68.3	165	239	-8.1	0.69<br>(0.5-0.95)	0.02
	LD	83.3	250	303	6.9	1.46<br>(1.04-2.04)	0.03
	PostLD	73.5	103	137	-2.9	0.94<br>(0.62-1.43)	0.76
Shetland Islands	2019	77.2	169	219	NA	NA	NA
	PreLD	87	27	31	9.8	2<br>(0.67-5.98)	0.22

	LD	85.2	56	65	8	2.22<br>(1.76-2.79)	<0.001
	PostLD	42.8	13	31	-34.5	1.74<br>(1.27-2.39)	<0.001
South Ayrshire	2019	82.1	760	926	NA	NA	NA
	PreLD	79.6	149	180	-2.5	1.05<br>(0.69-1.6)	0.82
	LD	90.5	271	301	8.4	1.97<br>(1.31-2.98)	<0.001
	PostLD	85	103	122	2.9	1.18<br>(0.71-1.99)	0.52
South Lanarkshire	2019	76.6	2469	3225	NA	NA	NA
	PreLD	75	587	745	-1.6	1.14<br>(0.94-1.38)	0.19
	LD	85.1	893	1043	8.5	1.82<br>(1.51-2.21)	<0.001
	PostLD	80.6	438	545	4	1.25<br>(1-1.57)	0.05
West Dunbartonshire	2019	67.6	585	866	NA	NA	NA
	PreLD	75.7	129	190	8.1	1.02<br>(0.73-1.42)	0.93
	LD	84.5	238	281	16.9	2.66<br>(1.86-3.79)	<0.001
	PostLD	78.8	116	147	11.2	1.8<br>(1.18-2.74)	0.01
West Lothian	2019	67.2	1244	1850	NA	NA	NA
	PreLD	73.7	266	382	6.5	1.12<br>(0.88-1.42)	0.36
	LD	82.1	487	594	14.9	2.22<br>(1.76-2.79)	<0.001
	PostLD	77.6	193	247	10.4	1.74<br>(1.27-2.39)	<0.001
Western Isles	2019	74.1	149	201	NA	NA	NA
	PreLD	60.8	18	30	-13.3	0.52<br>(0.24-1.16)	0.11
	LD	91.6	62	69	17.5	0.64<br>(0.53-0.76)	<0.001
	PostLD	76.7	23	30	2.6	1.14<br>(0.88-1.49)	0.32

606 **D First dose MMR**

HSCP	Time period	% uptake (within 4 weeks)	Number received	Number eligible	% point change from 2019	OR compared to 2019 (94% CI)	p value
Aberdeen City	2019	55.1	1235	2243	NA	NA	NA
	PreLD	53.4	259	501	-1.7	0.87 (0.72-1.06)	0.17
	LD	69.3	553	795	14.2	1.87 (1.57-2.22)	<0.001
	PostLD	71.5	285	399	16.4	2.04 (1.62-2.57)	<0.001
Aberdeenshire	2019	49.4	1383	2798	NA	NA	NA
	PreLD	48	254	554	-1.4	0.87 (0.72-1.04)	0.12
	LD	56.8	536	936	7.4	1.37 (1.18-1.59)	<0.001
	PostLD	48.9	221	448	-0.5	1 (0.82-1.22)	0.97
Angus	2019	69.3	681	982	NA	NA	NA
	PreLD	72.1	182	250	2.8	1.18 (0.87-1.61)	0.29
	LD	85.6	311	365	16.3	2.55 (1.85-3.5)	<0.001
	PostLD	80	129	162	10.7	1.73 (1.15-2.59)	0.01
Argyll and Bute	2019	64.8	440	679	NA	NA	NA
	PreLD	74	112	157	9.2	1.35 (0.92-1.98)	0.12
	LD	78.6	178	226	13.8	2.01 (1.41-2.87)	<0.001
	PostLD	72.9	74	102	8.1	1.44 (0.9-2.28)	0.13
Clackmannanshire and Stirling	2019	70.8	902	1274	NA	NA	NA
	PreLD	73.4	179	242	2.6	1.17 (0.86-1.6)	0.32
	LD	83.5	377	449	12.7	2.16 (1.63-2.86)	<0.001
	PostLD	86.8	178	205	16	2.72 (1.78-4.15)	<0.001
Dumfries and Galloway	2019	72	907	1259	NA	NA	NA
	PreLD	71.7	198	272	-0.3	1.04 (0.77-1.39)	0.8

	LD	84	338	401	12	2.08<br>(1.55-2.8)	<0.001
	PostLD	79.7	178	224	7.7	1.5<br>(1.06-2.12)	0.02
Dundee City	2019	60.5	857	1416	NA	NA	NA
	PreLD	60.3	188	293	-0.2	1.17<br>(0.9-1.52)	0.24
	LD	76	388	510	15.5	2.07<br>(1.65-2.61)	<0.001
	PostLD	74.5	198	265	14	1.93<br>(1.43-2.59)	<0.001
East Ayrshire	2019	58.9	703	1194	NA	NA	NA
	PreLD	56.3	166	260	-2.6	1.23<br>(0.93-1.63)	0.14
	LD	69	306	441	10.1	1.58<br>(1.25-2)	<0.001
	PostLD	70.4	154	218	11.5	1.68<br>(1.23-2.3)	<0.001
East Dunbartonshire	2019	75.8	803	1060	NA	NA	NA
	PreLD	79.3	188	240	3.5	1.16<br>(0.83-1.62)	0.4
	LD	91.1	303	335	15.3	3.03<br>(2.05-4.48)	<0.001
	PostLD	86.8	162	187	11	2.07<br>(1.33-3.23)	<0.001
East Lothian	2019	71.2	800	1124	NA	NA	NA
	PreLD	70.7	166	233	-0.5	1<br>(0.73-1.37)	0.98
	LD	87.3	352	405	16.1	2.69<br>(1.96-3.69)	<0.001
	PostLD	80.2	157	198	9	1.55<br>(1.07-2.24)	0.02
East Renfrewshire	2019	74.6	745	998	NA	NA	NA
	PreLD	76.6	157	199	2	1.27<br>(0.88-1.84)	0.21
	LD	84.8	280	327	10.2	2.02<br>(1.44-2.84)	<0.001
	PostLD	85.2	136	159	10.6	2.01<br>(1.26-3.19)	<0.001
Edinburgh	2019	68.1	3140	4611	NA	NA	NA
	PreLD	68.1	709	1037	0	1.01	0.86

						(0.88-1.17)	
	LD	80.1	1315	1642	12	1.88 (1.64-2.16)	<0.001
	PostLD	73.1	562	766	5	1.29 (1.09-1.53)	<0.001
Falkirk	2019	68.8	1052	1530	NA	NA	NA
	PreLD	67.8	245	343	-1	1.14 (0.88-1.47)	0.33
	LD	85.4	450	530	16.6	2.56 (1.97-3.32)	<0.001
	PostLD	80.8	216	265	12	2 (1.44-2.78)	<0.001
Fife	2019	67	2379	3553	NA	NA	NA
	PreLD	70.9	535	762	3.9	1.16 (0.98-1.38)	0.08
	LD	70.5	856	1214	3.5	1.18 (1.02-1.36)	0.02
	PostLD	68	414	608	1	1.05 (0.88-1.27)	0.58
Glasgow City	2019	67	4209	6281	NA	NA	NA
	PreLD	76.4	1079	1413	9.4	1.59 (1.39-1.82)	<0.001
	LD	80.6	1942	2404	13.6	2.07 (1.85-2.32)	<0.001
	PostLD	80.4	921	1146	13.4	2.02 (1.73-2.35)	<0.001
Highland	2019	56.7	1156	2039	NA	NA	NA
	PreLD	55.1	254	434	-1.6	1.08 (0.87-1.33)	0.48
	LD	68.5	511	746	11.8	1.66 (1.39-1.98)	<0.001
	PostLD	58	216	371	1.3	1.06 (0.85-1.33)	0.58
Inverclyde	2019	75.7	535	707	NA	NA	NA
	PreLD	62.6	100	151	-13.1	0.63 (0.43-0.92)	0.02
	LD	82	161	195	6.3	1.52 (1.01-2.29)	0.04
	PostLD	82.1	87	106	6.4	1.47 (0.87-2.49)	0.15
Midlothian	2019	65.3	733	1122	NA	NA	NA

	PreLD	64.8	177	258	-0.5	1.16 (0.87-1.55)	0.32
	LD	82.8	349	421	17.5	2.57 (1.94-3.41)	<0.001
	PostLD	83.5	163	196	18.2	2.62 (1.77-3.89)	<0.001
Moray	2019	54.1	447	827	NA	NA	NA
	PreLD	62.7	111	181	8.6	1.35 (0.97-1.87)	0.08
	LD	86.4	264	308	32.3	5.1 (3.6-7.22)	<0.001
	PostLD	86.3	130	150	32.2	5.53 (3.38-9.02)	<0.001
North Ayrshire	2019	56.5	648	1146	NA	NA	NA
	PreLD	58.6	144	253	2.1	1.02 (0.77-1.34)	0.91
	LD	73	272	373	16.5	2.07 (1.6-2.67)	<0.001
	PostLD	61.2	144	243	4.7	1.12 (0.84-1.48)	0.44
North Lanarkshire	2019	69.8	2448	3509	NA	NA	NA
	PreLD	68.3	566	791	-1.5	1.09 (0.92-1.29)	0.32
	LD	83.4	1078	1286	13.6	2.25 (1.9-2.65)	<0.001
	PostLD	78.4	488	624	8.6	1.56 (1.27-1.91)	<0.001
Orkney Islands	2019	53.9	111	206	NA	NA	NA
	PreLD	55.7	19	33	1.8	1.16 (0.55-2.44)	0.69
	LD	73.2	50	67	19.3	2.8 (2.23-3.53)	<0.001
	PostLD	71.6	29	41	17.7	1.89 (1.42-2.51)	<0.001
Perth and Kinross	2019	66.4	855	1287	NA	NA	NA
	PreLD	65.6	177	263	-0.8	1.04 (0.78-1.38)	0.79
	LD	78.2	351	448	11.8	1.83 (1.42-2.35)	<0.001
	PostLD	72.7	156	217	6.3	1.29 (0.94-1.78)	0.11
Renfrewshire	2019	69.1	1210	1750	NA	NA	NA

	PreLD	73.2	301	400	4.1	1.36 (1.06-1.74)	0.02
	LD	83.5	507	607	14.4	2.26 (1.78-2.87)	<0.001
	PostLD	74.9	216	287	5.8	1.36 (1.02-1.81)	0.04
Scottish Borders	2019	67.1	658	981	NA	NA	NA
	PreLD	66.9	134	204	-0.2	0.94 (0.68-1.29)	0.7
	LD	78.7	250	317	11.6	1.83 (1.36-2.47)	<0.001
	PostLD	70.8	114	154	3.7	1.4 (0.95-2.05)	0.09
Shetland Islands	2019	29.8	68	228	NA	NA	NA
	PreLD	54.1	19	35	24.3	2.79 (1.36-5.76)	0.01
	LD	39.5	27	69	9.7	2.8 (2.23-3.53)	<0.001
	PostLD	24.5	12	41	-5.3	1.89 (1.42-2.51)	<0.001
South Ayrshire	2019	61.5	583	948	NA	NA	NA
	PreLD	70.8	128	182	9.3	1.48 (1.05-2.09)	0.02
	LD	81.4	274	333	19.9	2.91 (2.13-3.97)	<0.001
	PostLD	78.6	132	169	17.1	2.23 (1.52-3.29)	<0.001
South Lanarkshire	2019	70.1	2271	3241	NA	NA	NA
	PreLD	71.3	548	731	1.2	1.28 (1.06-1.54)	0.01
	LD	81.8	960	1171	11.7	1.94 (1.64-2.3)	<0.001
	PostLD	81	460	567	10.9	1.84 (1.47-2.3)	<0.001
West Dunbartonshire	2019	65.8	573	871	NA	NA	NA
	PreLD	75.6	136	185	9.8	1.44 (1.01-2.06)	0.04
	LD	79.4	249	311	13.6	2.09 (1.53-2.85)	<0.001
	PostLD	78	128	164	12.2	1.85 (1.25-2.75)	<0.001

West Lothian	2019	66.7	1238	1857	NA	NA	NA
	PreLD	70.1	301	418	3.4	1.29<br>(1.02-1.63)	0.04
	LD	84.6	583	687	17.9	2.8<br>(2.23-3.53)	<0.001
	PostLD	79.1	257	325	12.4	1.89<br>(1.42-2.51)	<0.001
Western Isles	2019	52.6	113	215	NA	NA	NA
	PreLD	64.6	24	38	12	1.55<br>(0.76-3.15)	0.23
	LD	77.1	55	72	24.5	1.87<br>(1.57-.22)	<0.001
	PostLD	63.2	23	35	10.6	2.04<br>(1.62-2.57)	<0.001

E Second dose MMR

HSCP	Time period	% uptake (within 4 weeks)	Number received	Number eligible	% point change from 2019	OR compared to 2019 (95% CI)	p value
Angus	2019	43.8	498	1137	NA	NA	NA
	PreLD	54.7	147	275	10.9	1.47<br>(1.13-1.92)	<0.001
	LD	74.1	266	367	30.3	3.38<br>(2.61-4.37)	<0.001
	PostLD	62.7	118	189	18.9	2.13<br>(1.55-2.93)	<0.001
Argyll and Bute	2019	57.3	436	761	NA	NA	NA
	PreLD	55.8	92	166	-1.5	0.93<br>(0.66-1.3)	0.66
	LD	61.2	169	279	3.9	1.15<br>(0.87-1.51)	0.34
	PostLD	60.8	65	108	3.5	1.13<br>(0.75-1.7)	0.57
Clackmannanshire and Stirling	2019	52.3	770	1471	NA	NA	NA
	PreLD	54.7	193	320	2.4	1.38<br>(1.08-1.77)	0.01

	LD	68.3	341	504	16	1.9 (1.54-2.36)	<0.001
	PostLD	60.5	144	237	8.2	1.41 (1.07-1.87)	0.02
Dumfries and Galloway	2019	63.2	871	1379	NA	NA	NA
	PreLD	60.6	199	314	-2.6	1.01 (0.78-1.3)	0.94
	LD	71	308	437	7.8	1.39 (1.1-1.76)	0.01
	PostLD	72.5	187	257	9.3	1.56 (1.16-2.09)	<0.001
Dundee City	2019	32.9	485	1476	NA	NA	NA
	PreLD	39.4	143	346	6.5	1.44 (1.13-1.83)	<0.001
	LD	58.9	309	523	26	2.95 (2.4-3.62)	<0.001
	PostLD	52.8	137	256	19.9	2.35 (1.8-3.08)	<0.001
East Ayrshire	2019	39.7	522	1316	NA	NA	NA
	PreLD	26.6	100	304	-13.1	0.75 (0.57-0.97)	0.03
	LD	43.8	192	445	4.1	1.15 (0.93-1.44)	0.2
	PostLD	43.5	86	198	3.8	1.17 (0.86-1.58)	0.31
East Dunbartonshire	2019	56.4	674	1196	NA	NA	NA
	PreLD	63.6	197	300	7.2	1.48 (1.14-1.93)	<0.001
	LD	82.6	370	447	26.2	3.72 (2.84-4.88)	<0.001
	PostLD	85.1	188	223	28.7	4.16 (2.85-6.07)	<0.001
East Lothian	2019	60	762	1271	NA	NA	NA
	PreLD	56.2	161	271	-3.8	0.98 (0.75-1.28)	0.87
	LD	78.5	315	401	18.5	2.45 (1.88-3.18)	<0.001
	PostLD	59.1	112	184	-0.9	1.04 (0.76-1.43)	0.81
East Renfrewshire	2019	62.5	731	1169	NA	NA	NA
	PreLD	63.3	176	258	0.8	1.29	0.09

						(0.96-1.71)	
	LD	83.5	309	369	21	3.09 (2.28-4.17)	<0.001
	PostLD	84.1	156	186	21.6	3.12 (2.07-4.69)	<0.001
Edinburgh	2019	56.3	2727	4846	NA	NA	NA
	PreLD	60.5	660	1117	4.2	1.12 (0.98-1.28)	0.09
	LD	65.6	1103	1679	9.3	1.49 (1.33-1.67)	<0.001
	PostLD	61.3	492	806	5	1.22 (1.05-1.42)	0.01
Falkirk	2019	49	793	1619	NA	NA	NA
	PreLD	47.6	194	366	-1.4	1.17 (0.94-1.47)	0.16
	LD	62	353	579	13	1.63 (1.34-1.97)	<0.001
	PostLD	62.8	178	284	13.8	1.75 (1.35-2.27)	<0.001
Fife	2019	47	1799	3826	NA	NA	NA
	PreLD	50.4	446	906	3.4	1.09 (0.95-1.26)	0.23
	LD	52.2	673	1285	5.2	1.24 (1.09-1.41)	<0.001
	PostLD	44.7	257	573	-2.3	0.92 (0.77-1.09)	0.33
Glasgow City	2019	50.9	3262	6411	NA	NA	NA
	PreLD	61.8	960	1543	10.9	1.59 (1.42-1.78)	<0.001
	LD	70.8	1682	2378	19.9	2.33 (2.11-2.58)	<0.001
	PostLD	72.6	782	1077	21.7	2.56 (2.22-2.95)	<0.001
Highland	2019	50.7	1144	2257	NA	NA	NA
	PreLD	31.8	223	516	-18.9	0.74 (0.61-0.9)	<0.001
	LD	58.4	474	803	7.7	1.4 (1.19-1.65)	<0.001
	PostLD	52.3	192	356	1.6	1.14 (0.91-1.43)	0.26
Inverclyde	2019	61.9	445	719	NA	NA	NA

	PreLD	63.2	111	177	1.3	1.04 (0.74-1.45)	0.84
	LD	71.3	153	214	9.4	1.54 (1.11-2.15)	0.01
	PostLD	66.4	91	128	4.5	1.51 (1-2.28)	0.05
Midlothian	2019	60.2	736	1223	NA	NA	NA
	PreLD	56.8	168	293	-3.4	0.89 (0.69-1.15)	0.37
	LD	75.8	299	405	15.6	1.87 (1.45-2.4)	<0.001
	PostLD	68	137	204	7.8	1.35 (0.99-1.85)	0.06
North Ayrshire	2019	39.4	526	1334	NA	NA	NA
	PreLD	35.4	100	280	-4	0.85 (0.65-1.12)	0.25
	LD	47.7	205	427	8.3	1.42 (1.14-1.77)	<0.001
	PostLD	42.7	94	224	3.3	1.11 (0.83-1.48)	0.47
North Lanarkshire	2019	53	1986	3749	NA	NA	NA
	PreLD	53.1	508	845	0.1	1.34 (1.15-1.56)	<0.001
	LD	70.7	888	1252	17.7	2.17 (1.89-2.49)	<0.001
	PostLD	66.7	436	656	13.7	1.76 (1.48-2.09)	<0.001
Orkney Islands	2019	37	71	192	NA	NA	NA
	PreLD	50.6	16	33	13.6	1.6 (0.76-3.37)	0.21
	LD	63.6	43	66	26.6	2.46 (2.02-2.99)	<0.001
	PostLD	77.3	24	31	40.3	1.6 (1.25-2.04)	<0.001
Perth and Kinross	2019	45.5	654	1436	NA	NA	NA
	PreLD	51.5	190	356	6	1.37 (1.08-1.73)	0.01
	LD	63.2	284	465	17.7	1.88 (1.52-2.32)	<0.001
	PostLD	59.1	121	203	13.6	1.76 (1.31-2.38)	<0.001
Renfrewshire	2019	57.5	1064	1852	NA	NA	NA

	PreLD	60.9	252	408	3.4	1.2<br>(0.96-1.49)	0.11
	LD	70.6	445	627	13.1	1.81<br>(1.49-2.2)	<0.001
	PostLD	68.8	215	310	11.3	1.68<br>(1.29-2.17)	<0.001
Scottish Borders	2019	55.5	616	1110	NA	NA	NA
	PreLD	51.9	122	224	-3.6	0.96<br>(0.72-1.28)	0.78
	LD	64.6	218	335	9.1	1.49<br>(1.16-1.93)	<0.001
	PostLD	59.1	111	189	3.6	1.14<br>(0.83-1.56)	0.41
Shetland Islands	2019	24.5	68	278	NA	NA	NA
	PreLD	38	16	42	13.5	1.9<br>(0.96-3.75)	0.06
	LD	19	16	70	-5.5	2.46<br>(2.02-2.99)	<0.001
	PostLD	19.9	9	44	-4.6	1.6<br>(1.25-2.04)	<0.001
South Ayrshire	2019	42.6	447	1050	NA	NA	NA
	PreLD	37.3	100	244	-5.3	0.94<br>(0.71-1.24)	0.65
	LD	54.6	187	354	12	1.51<br>(1.19-1.92)	<0.001
	PostLD	56.4	94	166	13.8	1.76<br>(1.27-2.45)	<0.001
South Lanarkshire	2019	53.3	1839	3452	NA	NA	NA
	PreLD	52	453	798	-1.3	1.15<br>(0.99-1.35)	0.07
	LD	67.7	844	1247	14.4	1.84<br>(1.6-2.1)	<0.001
	PostLD	64.5	364	563	11.2	1.6<br>(1.33-1.93)	<0.001
West Dunbartonshire	2019	53	529	998	NA	NA	NA
	PreLD	62	125	200	9	1.48<br>(1.08-2.02)	0.01
	LD	68.4	203	294	15.4	1.98<br>(1.5-2.61)	<0.001
	PostLD	71	111	152	18	2.4<br>(1.64-3.51)	<0.001

West Lothian	2019	59.3	1238	2089	NA	NA	NA
	PreLD	59.3	291	493	0	0.99 (0.81-1.21)	0.9
	LD	79	576	737	19.7	2.46 (2.02-2.99)	<0.001
	PostLD	69.9	239	342	10.6	1.6 (1.25-2.04)	<0.001
Western Isles	2019	45.6	104	228	NA	NA	NA
	PreLD	41.4	20	48	-4.2	0.85 (0.45-1.6)	0.62
	LD	47.8	43	89	2.1	3.38 (2.61-4.37)	<0.001
	PostLD	64.1	27	42	18.5	2.13 (1.55-2.93)	<0.001

Supplementary Table S3

Immunisation	Deprivation quintile	Time period	% received within 4 weeks (Number received/total eligible)	% point change from 2019	OR (95%CI) for uptake compared to 2019	p-value
**First 6in1**	1 - most deprived	2019	91.9 (11025/11996)	NA	NA	NA
	1 - most deprived	PreLD	91.5 (2383/2580)	-0.4	1.1 (0.9-1.3)	0.44
	1 - most deprived	LD	93.9 (3815/4067)	2	1.3 (1.2-1.5)	<0.001
	1 - most deprived	PostLD	94 (1906/2026)	2.1	1.4 (1.2-1.7)	<0.001
	2	2019	93.1 (9740/10461)	NA	NA	NA
	2	PreLD	91.6 (2119/2289)	-1.5	0.9 (0.8-1.1)	0.36
	2	LD	95.1 (3311/3484)	2	1.4 (1.2-1.7)	<0.001
	2	PostLD	93.9 (1641/1746)	0.8	1.2 (0.9-1.4)	0.18
	3	2019	93.7 (8457/9030)	NA	NA	NA
	3	PreLD	93.1 (1814/1931)	-0.6	1.1 (0.9-1.3)	0.64
	3	LD	95.2 (3070/3224)	1.5	1.4 (1.1-1.6)	<0.001
	3	PostLD	95 (1468/1546)	1.3	1.3 (1-1.6)	0.05
	4	2019	95.6 (9804/10251)	NA	NA	NA
	4	PreLD	95.3 (2038/2142)	-0.3	0.9 (0.7-1.1)	0.31
	4	LD	95.9 (3328/3470)	0.3	1.1 (0.9-1.3)	0.5
	4	PostLD	94.4 (1677/1776)	-1.2	0.8 (0.6-1)	0.02
	5 - least deprived	2019	96.3 (8442/8769)	NA	NA	NA
	5 - least deprived	PreLD	95.7 (1724/1794)	-0.6	1(0.7-1.3)	0.73
	5 - least deprived	LD	96.7 (2759/2853)	0.4	1.1 (0.9-1.4)	0.28
	5 - least deprived	PostLD	96.3 (1373/1427)	0	1 (0.7-1.3)	0.92
**Second 6in1**	1 - most deprived	2019	79.8 (9633/12078)	NA	NA	NA

	1 - most deprived	PreLD	78 (1984/2492)	-1.8	1(0.9-1.1)	0.87
	1 - most deprived	LD	86.3 (3533/4096)	6.5	1.6 (1.4-1.8)	<0.001
	1 - most deprived	PostLD	86.3 (1737/2013)	6.5	1.6 (1.4-1.8)	<0.001
	2	2019	82.9 (8702/10499)	NA	NA	NA
	2	PreLD	83.4 (1880/2256)	0.5	1 (0.9-1.2)	0.61
	2	LD	88.4 (3133/3549)	5.5	1.6 (1.4-1.7)	<0.001
	2	PostLD	86.9 (1509/1736)	4	1.4 (1.2-1.6)	<0.001
	3	2019	85.2 (7711/9055)	NA	NA	NA
	3	PreLD	85.4 (1675/1968)	0.2	1 (0.9-1.1)	0.96
	3	LD	89.5 (2851/3182)	4.3	1.5 (1.3-1.7)	<0.001
	3	PostLD	89.4 (1387/1553)	4.2	1.5 (1.2-1.7)	<0.001
	4	2019	88.5 (9157/10348)	NA	NA	NA
	4	PreLD	89 (1892/2121)	0.5	1.1 (0.9-1.3)	0.35
	4	LD	92.2 (3221/3494)	3.7	1.5 (1.3-1.8)	<0.001
	4	PostLD	90.2 (1540/1708)	1.7	1.2 (1-1.4)	0.04
	5 - least deprived	2019	89.1 (7925/8890)	NA	NA	NA
	5 - least deprived	PreLD	88.5 (1655/1837)	-0.6	1.1 (0.9-1.3)	0.23
	5 - least deprived	LD	93.3 (2668/2862)	4.2	1.7 (1.4-2)	<0.001
	5 - least deprived	PostLD	91.7 (1280/1395)	2.6	1.4 (1.1-1.7)	<0.001
**Third 6in1**	1 - most deprived	2019	66.2 (8007/12102)	NA	NA	NA
	1 - most deprived	PreLD	65.5 (1727/2640)	-0.7	1 (0.9-1.1)	0.46
	1 - most deprived	LD	77.1 (3175/4114)	10.9	1.7 (1.6-1.9)	<0.001
	1 - most deprived	PostLD	74.9 (1427/1905)	8.7	1.5 (1.4-1.7)	<0.001
	2	2019	70.3	NA	NA	NA

			(7432/10569)			
	2	PreLD	68.5 (1607/2331)	-1.8	0.9 (0.9-1)	0.19
	2	LD	80 (2836/3550)	9.7	1.7 (1.5-1.8)	<0.001
	2	PostLD	79.4 (1336/1683)	9.1	1.6 (1.4-1.8)	<0.001
	3	2019	73.2 (6670/9107)	NA	NA	NA
	3	PreLD	74.3 (1499/2045)	1.1	1 (0.9-1.1)	0.96
	3	LD	82 (2610/3178)	8.8	1.7 (1.5-1.9)	<0.001
	3	PostLD	80.6 (1242/1542)	7.4	1.5 (1.3-1.7)	<0.001
	4	2019	77.5 (7991/10307)	NA	NA	NA
	4	PreLD	77.5 (1799/2322)	0	1 (0.9-1.1)	0.96
	4	LD	86.2 (2911/3376)	8.7	1.8 (1.6-2)	<0.001
	4	PostLD	82.7 (1414/1709)	5.2	1.4 (1.2-1.6)	<0.001
	5 - least deprived	2019	79.7 (7091/8893)	NA	NA	NA
	5 - least deprived	PreLD	80.9 (1626/2032)	1.2	1 (0.9-1.1)	0.77
	5 - least deprived	LD	87 (2478/2841)	7.3	1.7 (1.5-2)	<0.001
	5 - least deprived	PostLD	85.2 (1121/1316)	5.5	1.5 (1.2-1.7)	<0.001
**First MMR**	1 - most deprived	2019	62.4 (7561/12121)	NA	NA	NA
	1 - most deprived	PreLD	63.8 (1790/2721)	1.4	1.2 (1.1-1.3)	<0.001
	1 - most deprived	LD	73.7 3217/4358)	11.3	1.7 (1.6-1.8)	<0.001
	1 - most deprived	PostLD	70.6 (1529/2168)	8.2	1.4 (1.3-1.6)	<0.001
	2	2019	63.8 (6720/10533)	NA	NA	NA
	2	PreLD	64.6 (1579/2378)	0.8	1.1 (1-1.2)	0.02
	2	LD	76.2 (2920/3825)	12.4	1.8 (1.7-2)	<0.001
	2	PostLD	72.9 (1325/1814)	9.1	1.5 (1.4-1.7)	<0.001

	3	2019	64 (5936/9281)	NA	NA	NA
	3	PreLD	68.1 (1376/2024)	4.1	1.2 (1.1-1.3)	<0.001
	3	LD	77.9 (2541/3251)	13.9	2 (1.8-2.2)	<0.001
	3	PostLD	74.6 (1260/1685)	10.6	1.7 (1.5-1.9)	<0.001
	4	2019	66.5 (7102/10673)	NA	NA	NA
	4	PreLD	67.9 (1597/2292)	1.4	1.2 (1-1.3)	<0.001
	4	LD	80.5 (3041/3776)	14	2.1 (1.9-2.3)	<0.001
	4	PostLD	76.6 (1379/1801)	10.1	1.6 (1.5-1.8)	<0.001
	5 - least deprived	2019	70.4 (6563/9325)	NA	NA	NA
	5 - least deprived	PreLD	74 (1423/1927)	3.6	1.2 (1.1-1.3)	<0.001
	5 - least deprived	LD	85.1 (2730/3208)	14.7	2.4 (2.2-2.7)	<0.001
	5 - least deprived	PostLD	79 (1238/1566)	8.6	1.6 (1.4-1.8)	<0.001
**Second MMR**	1 - most deprived	2019	46 (5785/12580)	NA	NA	NA
	1 - most deprived	PreLD	47.9 (1482/2935)	1.9	1.2 (1.1-1.3)	<0.001
	1 - most deprived	LD	60.3 (2727/4538)	14.3	1.8 (1.7-1.9)	<0.001
	1 - most deprived	PostLD	57.7 (1236/2139)	11.7	1.6 (1.5-1.8)	<0.001
	2	2019	50.2 (5169/10291)	NA	NA	NA
	2	PreLD	53.3 (1270/2300)	3.1	1.2 (1.1-1.3)	<0.001
	2	LD	63.7 (2276/3570)	13.5	1.7 (1.6-1.9)	<0.001
	2	PostLD	60.3 (1001/1661)	10.1	1.5 (1.4-1.7)	<0.001
	3	2019	51.7 (4701/9100)	NA	NA	NA
	3	PreLD	51.8 (1127/2099)	0.1	1.2 (1.1-1.3)	<0.001
	3	LD	64.5 (1937/3007)	12.8	2 (1.8-2.2)	<0.001
	3	PostLD	62.3	10.6	1.7 (1.5-1.9)	<0.001

			(916/1470)			
	4	2019	54.2 (4998/9219)	NA	NA	NA
	4	PreLD	53.3 (1219/2135)	-0.9	1.1 (1-1.2)	0.02
	4	LD	69.8 (2238/3211)	15.6	1.9 (1.8-2.1)	<0.001
	4	PostLD	66.4 (1014/1528)	12.2	1.7 (1.5-1.9)	<0.001
	5 - least deprived	2019	59.4 (5148/8670)	NA	NA	NA
	5 - least deprived	PreLD	61.6 (1281/2010)	2.2	1.2 (1.1-1.3)	<0.001
	5 - least deprived	LD	75.6 (2105/2787)	16.2	2.1 (1.9-2.3)	<0.001
	5 - least deprived	PostLD	72 (1000/1391)	12.6	1.7 (1.5-2)	<0.001

Supplementary Table S4

Immunisation	Interaction term (baseline comparisons = 2019, SIMD 1)	ROR (exp of coeff of interaction model)	95% Confidence intervals	*p* -value
**First 6in1**	PreLD:SIMD2	0.87	(0.68-1.1) ns	0.23
	LD:SIMD2	1.06	(0.85-1.33) ns	0.59
	PostLD:SIMD2	0.83	(0.62-1.1) ns	0.2
	PreLD:SIMD3	0.99	(0.76-1.28) ns	0.92
	LD:SIMD3	1.01	(0.8-1.28) ns	0.91
	PostLD:SIMD3	0.91	(0.67-1.25) ns	0.56
	PreLD:SIMD4	0.84	(0.64-1.1) ns	0.2
	LD:SIMD4	0.8	(0.63-1.02) ns	0.07
	PostLD:SIMD4	0.55	(0.41-0.74) sig	<0.001
	PreLD:SIMD5	0.9	(0.66-1.22) ns	0.48
	LD:SIMD5	0.85	(0.65-1.12) ns	0.25
	PostLD:SIMD5	0.7	(0.5-1.01) ns	0.05
**Second 6in1**	PreLD:SIMD2	1.04	(0.89-1.23) ns	0.62
	LD:SIMD2	0.98	(0.84-1.14) ns	0.76
	PostLD:SIMD2	0.86	(0.7-1.05) ns	0.14
	PreLD:SIMD3	1.01	(0.85-1.2) ns	0.95
	LD:SIMD3	0.94	(0.8-1.11) ns	0.47
	PostLD:SIMD3	0.91	(0.73-1.13) ns	0.41
	PreLD:SIMD4	1.08	(0.9-1.3) ns	0.39
	LD:SIMD4	0.96	(0.81-1.14) ns	0.67
	PostLD:SIMD4	0.75	(0.6-0.93) sig	0.01

	PreLD:SIMD5	1.12	(0.92-1.36)<br>ns	0.27
	LD:SIMD5	1.05	(0.87-1.27)<br>ns	0.6
	PostLD:SIMD5	0.85	(0.67-1.08)<br>ns	0.18
**Third 6in1**	PreLD:SIMD2	0.97	(0.85-1.1)<br>ns	0.63
	LD:SIMD2	0.97	(0.86-1.1)<br>ns	0.62
	PostLD:SIMD2	1.06	(0.9-1.26)<br>ns	0.46
	PreLD:SIMD3	1.04	(0.9-1.19)<br>ns	0.61
	LD:SIMD3	0.97	(0.85-1.11)<br>ns	0.66
	PostLD:SIMD3	0.99	(0.83-1.18)<br>ns	0.92
	PreLD:SIMD4	1.03	(0.9-1.19)<br>ns	0.67
	LD:SIMD4	1.05	(0.92-1.2)<br>ns	0.49
	PostLD:SIMD4	0.91	(0.77-1.08)<br>ns	0.29
	PreLD:SIMD5	1.05	(0.91-1.22)<br>ns	0.51
	LD:SIMD5	1	(0.87-1.16)<br>ns	0.97
	PostLD:SIMD5	0.96	(0.79-1.16)<br>ns	0.66
**First MMR**	PreLD:SIMD2	0.97	(0.85-1.1)<br>ns	0.61
	LD:SIMD2	1.08	(0.96-1.21)<br>ns	0.21
	PostLD:SIMD2	1.07	(0.92-1.24)<br>ns	0.4
	PreLD:SIMD3	1.03	(0.9-1.18)<br>ns	0.65
	LD:SIMD3	1.19	(1.05-1.34)<br>sig	0.01
	PostLD:SIMD3	1.16	(0.99-1.35)<br>ns	0.06
	PreLD:SIMD4	1	(0.87-1.14)<br>ns	0.96
	LD:SIMD4	1.22	(1.09-1.38)<br>sig	<0.001
	PostLD:SIMD4	1.14	(0.98-1.33)	0.1

			ns	
	PreLD:SIMD5	1.02	(0.89-1.18)<br>ns	0.73
	LD:SIMD5	1.41	(1.24-1.61)<br>sig	<0.001
	PostLD:SIMD5	1.1	(0.94-1.3)<br>ns	0.25
**Second MMR**	PreLD:SIMD2	1.02	(0.9-1.15)<br>ns	0.75
	LD:SIMD2	0.99	(0.89-1.09)<br>ns	0.78
	PostLD:SIMD2	0.93	(0.81-1.08)<br>ns	0.35
	PreLD:SIMD3	0.91	(0.8-1.03)<br>ns	0.12
	LD:SIMD3	0.96	(0.86-1.07)<br>ns	0.44
	PostLD:SIMD3	0.96	(0.83-1.11)<br>ns	0.61
	PreLD:SIMD4	0.94	(0.83-1.06)<br>ns	0.31
	LD:SIMD4	1.1	(0.98-1.23)<br>ns	0.09
	PostLD:SIMD4	1.04	(0.9-1.2)<br>ns	0.63
	PreLD:SIMD5	1	(0.88-1.14)<br>ns	0.96
	LD:SIMD5	1.19	(1.06-1.34)<br>sig	<0.001
	PostLD:SIMD5	1.09	(0.93-1.27)<br>ns	0.29

Supplementary table S5

Immunisation	Time period	% uptake<br>(no received/no eligible)	% point change from 2019	OR for uptake compared to 2019<br>(95% CI)	*p*-value
First6in1<br>(uptake by age 24weeks)	2019	97.9<br>(49542/50609)	NA	NA	NA
	Pre LD	97.7<br>(10514/10761)	-0.2	0.92<br>(0.8-1.06)	0.22
	LD	97.6<br>(16724/17133)	-0.3	0.88<br>(0.79-0.99)	0.03
	Post LD	97.5<br>(8319/8531)	-0.4	0.85<br>(0.73-0.98)	0.03
Second6in1<br>(uptake by age 28 weeks)	2019	96.7<br>(49291/50975)	NA	NA	NA
	Pre LD	96.2<br>(10306/10698)	-0.5	0.9<br>(0.8-1.01)	0.06
	LD	96.6<br>(16639/17222)	-0.1	0.98<br>(0.89-1.07)	0.61
	Post LD	96.6<br>(8125/8412)	-0.1	0.97<br>(0.85-1.1)	0.61
Third6in1<br>(uptake by age 32 weeks)	2019	94<br>(48029/51085)	NA	NA	NA
	Pre LD	94.1<br>(10728/11394)	0.1	1.02<br>(0.94-1.12)	0.59
	LD	94.8<br>(16199/17093)	0.8	1.15<br>(1.07-1.24)	<0.001
	Post LD	93.5<br>(7644/8172)	-0.5	0.92<br>(0.84-1.01)	0.09
FirstMMR<br>(uptake by age 16 months)	2019	91.1<br>(47386/52015)	NA	NA	NA
	Pre LD	91.3<br>(10389/11370)	0.2	1.03<br>(0.96-1.11)	0.36
	LD	92.5<br>(17076/18463)	1.4	1.2<br>(1.13-1.28)	<0.001
	Post LD	91.6<br>(8285/9047)	0.5	1.06<br>(0.98-1.15)	0.14
SecondMMR<br>(uptake by age 3years 8 months)	2019	80.8<br>(40376/49940)	NA	NA	NA
	Pre LD	83.2<br>(9471/11495)	2.4	1.11<br>(1.05-1.17)	<0.001
	LD	86.1<br>(14763/17145)	5.3	1.47<br>(1.4-1.54)	<0.001
	Post LD	84.4<br>(6915/8196)	3.6	1.28<br>(1.2-1.36)	<0.001

Supplementary table S6

Immunisation	Time period	% uptake<br>(no received/no eligible)	% point change from 2019	OR for uptake compared to 2019<br>(95% CI)	*p*-value
First6in1<br>(uptake by age 6 months)	2019	96.3<br>(571531/593700)	NA	NA	NA
	Pre LD	95.8<br>(132779/138608)	0.5	0.88 (0.86-0.91)	<0.001
	LD	95.8<br>(171660/179180)	0.5	0.89 (0.86-0.91)	<0.001
	Post LD	95.6<br>(90702/94862)	0.7	0.85 (0.82 -0.87)	<0.001
Second6in1<br>(uptake by age 6 months)	2019	93.9<br>(559382/595815)	NA	NA	NA
	Pre LD	92.1<br>(131578/142833)	1.8	0.76 (0.74-0.78)	<0.001
	LD	93.0<br>(164780/177221)	0.9	0.86 (0.84-0.88)	<0.001
	Post LD	92.5<br>(87818/94960)	1.4	0.80 (0.78-0.82)	<0.001
Third6in1<br>(uptake by age 6 months)	2019	88.7<br>(529163/596355)	NA	NA	NA
	Pre LD	85.6<br>(126284/147479)	3.1	0.76 (0.74-0.77)	<0.001
	LD	86.6<br>(154912/178953)	2.1	0.82 (0.81-0.83)	<0.001
	Post LD	86.0<br>(79991/92990)	2.7	0.78 (0.77-0.80)	<0.001
FirstMMR<br>(uptake by age 18 months)	2019	88.1<br>(541107/613923)	NA	NA	NA
	Pre LD	86.7<br>(119885/138353)	1.4	0.87(0.86-0.89)	<0.001
	LD	86.2<br>(173451/201164)	1.9	0.84(0.83-0.85)	<0.001

	Post LD	86.2 (85939/99685)	1.9	0.84(0.83-0.86)	<0.001

S1 File

Impact of COVID-19 and associated control measures on uptake of pre-school immunisations

Analysis Plan

**Full Project Title**	Impact of COVID-19 and associated control measures on uptake of pre-school immunisations
**Version Number**	1.0
**Previous Versions**	0.2
**Effective Date**	27/01/2021
**EAVE II Sub-study**	
**EAVE II Analyst(s)**	Rachel Mulholland (UofE)
**Co-authors**	Scotland:<br>Fiona McQuaid* (NHS Lothian, UofE)<br>Claire Cameron (PHS)<br>Cheryl Gibbon (PHS)<br>Aziz Sheikh (UofE)<br>Judith Tait (PHS)<br>Steve Turner (RCPCH, University of Aberdeen)<br>Jaime Villacampa Ortega (PHS)<br>Rachael Wood (PHS, UofE)<br><br>England:<br>Helen Bedford (ICH, London)<br>Elise Tessier (PHE)<br>Yuma Sanpang Rai (PHE)<br>Joanne White (PHE)

	Partho Roy (PHE)
**Target journal**	BMJ

*Corresponding author

Contents

1 Introduction 89
2 Aims and objectives 90
2.1 Aims 90
2.2 Objectives 90
3 Study Design 91
3.1 Study design 91
3.2 Setting 91
3.3 Population 91
3.4 Data sources 91
3.5 Inclusion/exclusion criteria 92
3.6 Sample size calculation 92
4 Data and data validation 93
4.1 Data variables available 93
4.2 Constructed variables 93
4.3 Consistency and error checking 94
5 Statistical analyses 95
5.1 Objective a: 95
5.1.1 Outcome 95
5.1.2 Exposures of interest 95
5.1.3 Analytical techniques 96
5.1.4 Potential confounders 98
5.1.5 Potential effect modifiers 98
5.1.6 Sub-group analysis 98
5.1.7 Corrections for multiple testing 98
5.1.8 Sensitivity analysis 98
5.1.9 Other analysis 98
5.2 Missing data 101
5.3 Statistical software 101
6 Reporting results 102
6.1 Reporting guidelines and conventions 102
6.2 Dissemination 102
7 References 103

1 Introduction

The COVID-19 pandemic and resulting national lockdown has had a profound impact on the delivery of healthcare with the reconfiguration of acute services to support the anticipated influx of COVID-19 patients, cancellation of most elective activity and pausing of screening programmes (1). There is also evidence of a change in healthcare-seeking behaviour, for example, in Scotland the uptake of both emergency and elective hospital based care dropped substantially over the lockdown period (2). However, within child health, key routine services such as childhood immunisations and health visiting continued across Scotland and a campaign launched in April 2020 urged the public to remember "The NHS is open" (https://www.gov.scot/news/urgent-medical-help-still-available).

It has become increasingly apparent that younger children are at low risk of severe disease due to SARS-CoV-2 (3, 4) and may be less susceptible to infection by the virus (5). Yet the wider impact of the pandemic on children in terms of education, mental and physical health and safeguarding is not yet fully understood, though it is likely to be profound (6, 7). One particular area of concern early in the lockdown period was the potential effect on the uptake of routine childhood immunisations (8). Maintaining high population vaccine coverage is vital for both direct and indirect (via herd immunity) protection against non-COVID-19 infectious diseases. The current pre-school UK vaccination schedule is shown in table 1 along with the disease protected against.

Table 1 UK immunisation schedule 2020 (9). NA= not referenced/used in this manuscript.

Age due	**Diseases protected against**	**Vaccine given**	**Referred in this manuscript as:**
8 weeks	Diphtheria, tetanus, pertussis (whooping cough), polio, Haemophilus influenzae type b (Hib) and hepatitis B	DTaP/IPV/Hib/HepB	First dose 6in1
	Meningococcal group B (MenB)	MenB	NA
	Rotavirus gastroenteritis	Rotavirus	NA
12 weeks	Diphtheria, tetanus, pertussis, polio, Hib and hepatitis B	DTaP/IPV/Hib/HepB	Second dose 6in1
	Pneumococcal (13 serotypes)	Pneumococcal conjugate vaccine (PCV)	NA
	Rotavirus gastroenteritis	Rotavirus	NA
16 weeks	Diphtheria, tetanus, pertussis, polio, Hib and hepatitis B	DTaP/IPV/Hib/HepB	Third dose 6in1
	Meningococcal group B (MenB)	MenB	NA
1 year (after first birthday)	Hib and Meningococcal group C (MenC)	Hib/MenC	NA
	Measles, mumps and rubella	MMR	First dose MMR
	Pneumococcal	PCV booster	NA
	MenB	MenB booster	NA
3 years 4 months (or shortly after)	Diphtheria, tetanus, pertussis and polio	dTaP/IPV	NA
	Measles, mumps and rubella	MMR booster	Second dose MMR

Initial reports suggested a fall in children receiving their scheduled vaccinations in the very early lockdown period (8). The aim of this paper is to use routinely collected and publicly available child health surveillance data in Scotland to describe the pattern of pre-school vaccine uptake during the pandemic period, with comparisons to 2019 data, by geographical area and socio-economic index. Of note, this paper refers to the first national lockdown which began on 23rd March 2020 with restrictions easing gradually from June 2020. The emergence in the UK of a new, highly transmissible, SARS-CoV-2 variant in late 2020 (10) has since prompted further control measures (essentially further lockdowns in Scotland and England with similar measure in Wales and Northern Ireland). Data continue to be collected on the impact on vaccine uptake and should be further evaluated when available, however this paper deals solely with the first national lockdown period.

We have contacted colleagues in child health surveillance from the other nations of the UK (England, Wales and Northern Ireland) to request access to equivalent data and should this become available, we will aim to describe the patterns of pre-school vaccine uptake as above for each of the nations which form the UK.

2 Aims and objectives

3 Aims

We aim to describe the impact of COVID-19 and associated control measures on the uptake of pre-school immunisations in Scotland, with potential to expand to other UK nations should additional data become available.

4 Objectives

We seek to:

a. Describe the impact of the COVID-19 pandemic on the uptake of selected immunisations provided at each of the five immunisation contacts offered to pre-school children (table 1)
b. Explore whether impact varied by: i) Health and social care partnership area of residence (HSCP- geographical areas closely related to local authority areas); and ii) socio-economic deprivation index
c. Use these data to inform future strategies to promote maximal childhood vaccine uptake and reduce barriers, both in the context of a pandemic and more generally.

5 Study Design

6 Study design

Natural experiment, designed to take advantage of routinely collected data in the year prior to the COVID-19 pandemic and immediately before, during the first period of ‘lockdown’ (23 March-July 2020) imposed by the Scottish and UK and other devolved governments, and after the lockdown restrictions began to be eased (August- September 2020).

7 Setting

Scotland, UK with potential scope to extend to England, Wales and Northern Ireland.

8 Population

All children in Scotland (and if available; England, Wales and Northern Ireland) who become eligible (based on age), for routine immunisations as shown in Table 1 (specifically; first, second and third dose 6 in1, and first and second dose MMR) from January 2019 to September 2020.

9 Data sources

The Public Health Scotland (PHS) COVID-19 wider impacts dashboard (https://scotland.shinyapps.io/phs-covid-wider-impact/), which presents data drawn from the Scottish Immunisation & Recall System (SIRS). SIRS is the dataset which records all information on children eligible for and receiving routine preschool immunisation in Scotland. These data are publicly available via the link above.

Equivalent data has been sought from England, Wales and Northern Ireland.

In England, data are extracted from ImmForm, a Public Health England website used to collect vaccine coverage data and provide vaccine ordering facilities. At present, due to data sharing agreements, it is likely that data from England will be analysed “in-house” by scientists at Public Health England, and results shared with the wider team. This analysis plan and the code used to analysis the Scottish data will be shared and similar methods applied within the constraints of which data are available (likely uptake of 6in1 vaccine at 6 months of age, and MMR at 12 and 18 months).

In Northern Ireland, data are collected by the Northern Ireland Child health System, the format and accessibility of which is being explored.

In Wales, data are provided by NHS Wales Informatics Service from the National Community Child Health Database (NCCHD). This is sourced from Community Child Health databases maintained by

local Child Health Office staff in Trusts throughout Wales based on regular returns from nurses and doctors who immunise or advise on immunisation.

10 Ethical approval

Ethical approval for this specific study was not required as we are using publicly available, anonymised, aggregated data.

11 Inclusion/exclusion criteria

All children living in Scotland (or other UK nation) who became eligible based on age for the relevant vaccine are included, with the exception of those registered to receive their second dose MMR immunisation in HSCPs associated with NHS Grampian. This is because this immunisation is offered to children when they turn 4 years of age in Grampian, rather than when they turn 3 years and 4 months of age as in all other Board areas.

Note 1: During this period, some children would become eligible for more than one set of immunisations (for example a child aged 8 weeks in January 2020, would become eligible for first, second and third doses of 6in1 vaccine during the study period). These children are included for each separate dose.

Note 2: A very small number of children would be medically exempt from the MMR vaccine as it is a live vaccine, however these children are still included as eligible for the purposes of this analysis.

Note 3: It is possible for parents to consent to receive selected vaccines only (for example to decline the MMR, but still receive the other vaccines offered at 1 year of age), therefore caution must be taken if extrapolating these data to represent uptake of the other pre-school immunisations for which data are not currently publicly available.

12 Sample size calculation

Taking in entire eligible population

13 Data and data validation

14 Data variables available

Table 2 Available variables (Scottish dataset)

Variable	Description	Values
Vaccine type	Type of preschool immunisation vaccine	First dose 6in1 (8 weeks)<br>Second dose 6in1 (12 weeks)<br>Third dose 6in1 (16 weeks)<br>First dose MMR (12 months)<br>Second dose MMR (3 years and 4 months)
Area name	Splits into Scotland as a whole, NHS Health Boards and HSCP	Scotland<br>NHS Health Boards<br>HSCP
Time-period	Time-period when preschool child was eligible for immunisation	2019 (baseline),<br>January to September 2020 (Monthly)<br>Week beginning 02 March to 28 September 2020 (Weekly)
Total eligible	The total number of eligible preschool children	N
Total uptake within 4 weeks of eligibility (N)*	The number of preschool children who received vaccination within 4 weeks of becoming eligible	N
Total uptake within 4 weeks of eligibility (%)	The number of preschool children who received vaccination within 4 weeks of becoming eligible out of the total number of eligible preschool children	% [0,100]
SIMD Quintile (for Scotland level data only)	Scottish Index of Multiple Deprivation (SMID) index	1 (most deprived), 2, 3, 4, 5 (least deprived)

*Note: Data are available for total uptake at later ages (for example 6 months of age). However, we have chosen to examine uptake within 4 weeks of eligibility as this represents timely uptake of vaccinations as per the recommended schedule.

15 Constructed variables

Table 3 Constructed variables

Variable	Description	Values
Absolute change from 2019 (%)	% uptake within 4 weeks of eligibility in 2020 – % uptake within 4 weeks of eligibility in 2019	[-100,100] %

Relative change from 2019 (%)	Absolute change from 2019 (%) / % uptake within 4 weeks of eligibility in 2019	[-1,Inf] %

16 Consistency and error checking

Data quality has already been checked by PHS.

17 Statistical analyses

The following sections are presented separately for each analytical objective. All analyses will be conducted for data from Scotland. Depending on the data available for the other UK nations, similar analyses will be conducted in parallel. At present, the variations in data (in terms of collection methods and availability and varying vaccination policies) suggest that pooling the UK data or conducting a meta-analysis would not be appropriate, however any differing trends may be discussed in a descriptive manner.

18 Objective a) Describe the impact of the COVID-19 pandemic on the uptake of immunisations provided at each of the five immunisation contacts offered to pre-school children

19 Outcome

The primary outcome is the % uptake of preschool immunisations (represented by uptake of one of the vaccines due at that age) within 4 weeks of eligibility. As a secondary outcome, the % uptake of preschool immunisations by 6 months of age (first dose 6in1) or 16 -18 months of age (first dose MMR) may also be considered to allow for comparisons with data from other nations (TBC).

20 Exposures of interest

Time, specifically in time-periods: 2019 (baseline), 01 Jan- 22 Mar 2020 (pre-lockdown), 23 Mar-final week Jul 2020 (lockdown) and Aug-end of Sept 2020 (post lockdown). The start date to 23 Mar has been chosen to correspond with the beginning of the UK wide lockdown as announced by the UK government. The end of the lockdown period is less well-defined and varied both in approach and timescale between Scotland, England, Wales and Northern Ireland. Broadly speaking, by the end of July, there was a substantial reduction in 'lockdown' restrictions with the opening of many non-essential businesses and limited indoor meeting between households permitted, therefore a pragmatic approach has been taken to define the lockdown period as 23 Mar 2020 until end Jul 2020. The data included in each time period are shown in Table 4.

Table 4 Definition of time periods

Time period	Data included
Baseline 2019	Aggregate data for whole of 2019
Pre-Lockdown 2020	Monthly data for Jan 2020 and Feb 2020.<br>Weekly data from W/B 2Mar2020 up to and including W/B 16Mar2020
Lockdown 2020	Weekly data from W/B 23Mar2020 up to and including W/B 27Jul2020
Post lockdown 2020	Weekly data from W/B 3Aug2020 up to and including W/B 28Sept2020

The primary outcome of interest is comparison between baseline 2019 and lockdown 2020 for each vaccine. Of secondary interest are comparisons between baseline 2019 and pre-lockdown 2020, baseline 2019 and post lockdown 2020, pre-lockdown 2020 and lockdown 2020, lockdown 2020 and post lockdown 2020.

21 Analytical techniques

To compare the % uptake of preschool immunisation across time-periods we will visualise the total % uptake within 4 weeks of eligibility for each time-period by vaccine in a bar plot.

To statistically test whether these uptake rates are different, we will perform a binary logistical regression analysis for aggregate data, using time period as the explanatory variable, and vaccination status (vaccinated or unvaccinated) as the dependent variable. Separate analyses will be carried out for each vaccine. Odds ratios with 95% confidence intervals will be calculates using 2019 as the baseline comparator.

The following tables will be utilised:

Table 5 Odds of being vaccinated between time-periods for each vaccine visit

Vaccine	Time-period comparison	Odds ratio	95% CI
First dose 6in1	2019 vs pre-lockdown (secondary outcome)		
	**2019 vs lockdown (primary outcome)**		
	Pre-lockdown vs lockdown (secondary outcome)		
	2019 vs post-lockdown (secondary outcome)		
	Lockdown vs post-lockdown (secondary outcome)		
Second dose 6in1	2019 vs pre-lockdown (secondary outcome)		
	**2019 vs lockdown (primary outcome)**		
	Pre-lockdown vs lockdown (secondary outcome)		
	2019 vs post-lockdown (secondary outcome)		
	Lockdown vs post-lockdown (secondary outcome)		
…			
Second dose MMR	2019 vs pre-lockdown (secondary outcome)		

	**2019 vs lockdown (primary outcome)**		
	Pre-lockdown vs lockdown (secondary outcome)		
	2019 vs post-lockdown (secondary outcome)		
	Lockdown vs post-lockdown (secondary outcome)		

22 Potential confounders

None available since aggregated data

23 Potential effect modifiers

None at this stage

24 Sub-group analysis

None

25 Corrections for multiple testing

None

26 Sensitivity analysis

None

27 Other analysis

None

28 Objective bi) Explore the difference in impact between geographical areas

29 Outcome

Absolute/relative difference in % uptake within 4 weeks of eligibility during lockdown vs 2019 for each Health and Social Care Partnership (HSCP).

30 Exposures of interest

Geographical area, specifically HSCP (excluding partnership areas within NHS Grampian for the MMR second dose only).

31 Analytical techniques

To visualise the spatial distribution of the difference in lockdown and 2019, the % differences will be plotted in a choropleth map of Scotland segmented into HSCPs. This map will have each HSCP area coloured according to the % difference. This will be repeated for each vaccine type.

To statistically test whether these uptake rates are different between time periods for each HSCP, we will perform a binary logistical regression analysis for aggregate data, using vaccination status (vaccinated or unvaccinated) as the dependent variable and time period as the explanatory variable, specifying an interaction with HSCP. Separate analyses will be carried out for each vaccine. Odds ratios with 95% confidence intervals will be calculated using 2019 as the baseline comparator and results will be visualised using forest plots. The following table will be used (one for each vaccine):

Table 6 Odds of being vaccinated between time-periods by HSCP

Time-period	Variable	Difference %	Odds ratio	95% CI
2019 vs lockdown	Aberdeen City			
	Aberdeenshire			
	Angus			
	…			

32 Potential confounders

None

33 Potential effect modifiers

None

34 Sub-group analysis

None

35 Corrections for multiple testing

None

36 Sensitivity analysis

None

37 Other analysis

None

38 Objective bii) Explore the difference in impact between socio-economic deprivation

39 Outcome

Absolute/relative difference in % uptake within 4 weeks of eligibility during lockdown vs total 2019 by SMID quintile.

40 Exposures of interest

Scottish Index of Multiple Deprivation (SIMD) quintiles

41 Analytical techniques

To explore the difference in the % uptake during lockdown (23Mar-Jul 2020) versus 2019 across the deprivation quintiles, we will plot the % difference for each vaccine by the SIMD quintile in a bar plot.

To statistically test whether these uptake rates are different between time periods for each SMID quintile, we will perform a binary logistical regression analysis for aggregate data, using time period as the explanatory variable, and vaccination status (vaccinated or unvaccinated) as the dependent variable, specifying an interaction with SIMD quintile. Separate analyses will be carried out for each vaccine. Odds ratios with 95% confidence intervals will be calculated using 2019 as the baseline comparator. The following tables will be utilised (one for each vaccine):

Table 7 Odds of being vaccinated between time-periods by SMID

Time-period	Variable	Difference %	Odds ratio	95% CI
2019 vs lockdown	SMID 1			
	SMID 2			
	SMID 3			
	SMID 4			
	SMID 5			

A similar technique will be carried out to determine the odds of being vaccinated by SMID quintile at each time point (2019 and lockdown), using SMID 1 (most deprived) as the baseline comparator, to assess for change in the inequality between SMID quintiles between the two time periods.

42 Potential confounders

None

43 Potential effect modifiers

None

44 Sub-group analysis

None

45 Corrections for multiple testing

None

46 Sensitivity analysis

None

47 Other analysis

None

48 Missing data

As noted in section 3.6, for analyses relating to the second dose of MMR only, data relating to children registered to receive their immunisations in HSCP within NHS Grampian will be excluded. This is because this immunisation is offered to children when they turn 4 years of age in Grampian, rather than when they turn 3 years and 4 months of age as in all other Board areas.

A small number of children have missing information on HSCP area of residence and/or SIMD quintile. Numbers with missing data will be provided as supporting information for the analyses by HSCP and SIMD. No information is provided through the PHS dashboard on immunisation uptake for children with missing HSCP/SIMD data so this will not be reported.

49 Statistical software

All analyses will be performed on R/R Studio (4.0.3)

50 Reporting results

51 Reporting guidelines and conventions

Results will be reported according to STROBE (11) and RECORD (12) (via the COVID-19 extension) guidelines. P-values will be quoted to two decimal places, unless they are less than 0.001 (whereby the p-value will be given as <0.001) or between <0.005 and >0.001, in which case they will be stated to three decimal places. We will report 95% confidence intervals and if the confidence intervals cross 1, the results will not be considered statistically significant.

52 Dissemination

The analysis will be written in a manuscript and submitted to a peer reviewed journal. The chosen journal we have decided to aim for is the BMJ.

We will distribute finding to leads for immunisation policy and delivery in the Scottish government, Public Health Scotland, and territorial NHS Boards through the Scottish Immunisation Group. Results will also be shared with equivalent bodies of other UK nations. Findings will also be discussed with the Royal College of Paediatrics and Child Health and *British Association for Child and Adolescent Public Health* and may be presented at local, regional, national and/or international meetings.

Key messages will be made into an infographic to be disseminated on social media and communication channels. All code will be made publicly available via the EAVE II GitHub (https://github.com/EAVE-II).

53 References

1. Scottish Government, Re-mobilise, Recoved, Re-design: the feamework for NHS Scotland, 2020.

2. Mulholland RH, Wood R, Stagg HR, Fischbacher C, Villacampa J, Simpson CR, et al. Impact of COVID-19 on accident and emergency attendances and emergency and planned hospital admissions in Scotland: an interrupted time-series analysis. J R Soc Med. 2020;113(11):444-53.

3. Swann OV, Holden KA, Turtle L, Pollock L, Fairfield CJ, Drake TM, et al. Clinical characteristics of children and young people admitted to hospital with covid-19 in United Kingdom: prospective multicentre observational cohort study. BMJ. 2020;370:m3249.

4. Dong Y, Mo X, Hu Y, Qi X, Jiang F, Jiang Z, et al. Epidemiology of COVID-19 Among Children in China. Pediatrics. 2020;145(6).

5. Viner RM, Mytton OT, Bonell C, Melendez-Torres GJ, Ward J, Hudson L, et al. Susceptibility to SARS-CoV-2 Infection Among Children and Adolescents Compared With Adults: A Systematic Review and Meta-analysis. JAMA Pediatr. 2020.

6. Araújo LA, Veloso CF, Souza MC, Azevedo JMC, Tarro G. The potential impact of the COVID-19 pandemic on child growth and development: a systematic review. J Pediatr (Rio J). 2020.

7. Royal College of Paediatrics and Child Health workforce team . The impact of COVID - 19 on child health services - report. 2020. Available from https://www.rcpch.ac.uk/resources/impact-covid-19-child-health-services-report

8. Saxena S, Skirrow H, Bedford H. Routine vaccination during covid-19 pandemic response. BMJ. 2020;369:m2392.

9. Public Health England. Complete routine immunisation schedule from January 2020 2020 [updated June 2020. Available from: https://assets.publishing.service.gov.uk/government/uploads/system/uploads/attachment_data/file/899423/PHE_Complete_Immunisation_Schedule_Jun2020_05.pdf.

10. Public Health England. Investigation of novel SARS - C o V - 2 variant. 2020.

11. von Elm E, Altman DG, Egger M, Pocock SJ, Gøtzsche PC, Vandenbroucke JP, et al. The Strengthening the Reporting of Observational Studies in Epidemiology (STROBE) statement: guidelines for reporting observational studies. J Clin Epidemiol. 2008;61(4):344-9.

12. Benchimol EI, Smeeth L, Guttmann A, Harron K, Moher D, Petersen I, et al. The REporting of studies Conducted using Observational Routinely-collected health Data (RECORD) statement. PLoS Med. 2015;12(10):e1001885.

S2 File Supplementary methods

Additional inclusion/exclusion criteria

During the study period, some children would become eligible for more than one set of immunisations (for example, a child aged 8 weeks in January 2020, would become eligible for first, second and third doses of 6in1 vaccine during the study period) and these children were included for each separate dose. Of note, a very small number of children would be medically exempt from the MMR vaccine as it is a live vaccine (15), however these children are still included as eligible for the purposes of this analysis. For the analyses of the second dose MMR only, we excluded children registered to receive this vaccine in Health and Social Partnerships associated with NHS Grampian (Aberdeen City, Aberdeenshire and Moray) as this immunisation is offered to children when they turn 4 years of age in Grampian, rather than when they turn 3 years and 4 months of age as in all other areas.

Additional information on data sources

Scotland

The "COVID19 wider impacts on the health care system" dashboard was set up by Public Health Scotland in response to the COVID-19 pandemic to provide near real-time updates from a range of national databases on topics ranging from child health to cancer, thus allowing for rapid analysis of trends (19). (18). Data for childhood immunisations were drawn monthly from the Scottish Immunisation & Recall System (SIRS), an electronic system used by all NHS boards in Scotland by which information on children eligible for and receiving routine preschool immunisation is recorded by administrative staff within the relevant NHS board (19). The code used by Public Health Scotland to produce the wider impacts dashboard can be accessed at https://github.com/Public-Health-Scotland/covid-wider-impacts.

Data were available for all children living in Scotland and eligible for the five immunisations of interest with the above noted exceptions. For 2019, aggregate data for the entire year were used, while for 2020, monthly data were used for January and February 2020, then weekly from 2nd March 2020 (table S1). For the island HSCPs (Shetland, Orkney and the Western Isles), and for England, only monthly data were available therefore lockdown was defined as the months April to July inclusive.

Of note, data checking by Public Health Scotland revealed that a changes to the reporting database to include scheduling for some adult immunisations meant that a small number of older adults born in 1920 were included in the denominator for the January 2020 data (further details can be found in the 23 December release commentary on https://scotland.shinyapps.io/phs-covid-wider-impact/). It was not possible to remove these older adult records, therefore, uptake rates for January 2020 may have been slightly under-reported. This fault was rectified by the next dashboard update in February.

S3 File

The RECORD statement – checklist of items, extended from the STROBE statement, that should be reported in observational studies using routinely collected health data.

	Item No.	STROBE items	Location in manuscript where items are reported	RECORD items	Location in manuscript where items are reported
**Title and abstract**					
	1	(a) Indicate the study's design with a commonly used term in the title or the abstract (b) Provide in the abstract an informative and balanced summary of what was done and what was found	Title page (page 1-3)<br>Abstract (page 4)	RECORD 1.1: The type of data used should be specified in the title or abstract. When possible, the name of the databases used should be included.<br><br>RECORD 1.2: If applicable, the geographic region and timeframe within which the study took place should be reported in the title or abstract.<br><br>RECORD 1.3: If linkage between databases was conducted for the study, this should be clearly stated in the title or abstract.	Title page (page 1-3)<br>Abstract (page 4)<br>Further details in methods (page 7)
**Introduction**					
Background rationale	2	Explain the scientific background and rationale for the investigation being reported	Introduction (page 6)		
Objectives	3	State specific objectives, including any prespecified hypotheses	Abstract (page 4)<br>Methods (page 7-8)		

			Pre-specified analysis plan (supplemental data)		
**Methods**					
Study Design	4	Present key elements of study design early in the paper	Methods (page 7-8)		
Setting	5	Describe the setting, locations, and relevant dates, including periods of recruitment, exposure, follow-up, and data collection	Methods (page 7-8)		
Participants	6	*(a) Cohort study* - Give the eligibility criteria, and the sources and methods of selection of participants. Describe methods of follow-up<br>*Case-control study* - Give the eligibility criteria, and the sources and methods of case ascertainment and control selection. Give the rationale for the choice of cases and controls<br>*Cross-sectional study* - Give the eligibility criteria, and the sources and methods of selection of participants<br><br>*(b) Cohort study* - For matched studies, give matching criteria and number of exposed and unexposed<br>*Case-control study* - For matched studies, give matching criteria and the number of controls per case	Methods (page 7-8)	RECORD 6.1: The methods of study population selection (such as codes or algorithms used to identify subjects) should be listed in detail. If this is not possible, an explanation should be provided.<br><br>RECORD 6.2: Any validation studies of the codes or algorithms used to select the population should be referenced. If validation was conducted for this study and not published elsewhere, detailed methods and results should be provided.<br><br>RECORD 6.3: If the study involved linkage of databases, consider use of a flow diagram or other graphical display to demonstrate the data linkage process, including the number of individuals with linked data at each stage.	Methods (page 7-8)<br>Code used to analysis data will be made publicly available at https://github.com/EAVE-II) (page 8)

Variables	7	Clearly define all outcomes, exposures, predictors, potential confounders, and effect modifiers. Give diagnostic criteria, if applicable.	Methods (page 7-8)	RECORD 7.1: A complete list of codes and algorithms used to classify exposures, outcomes, confounders, and effect modifiers should be provided. If these cannot be reported, an explanation should be provided.	Methods (page 7-8)<br>Code used to analysis data will be made publicly available at https://github.com/EAVE-II) (page 8)
Data sources/ measurement	8	For each variable of interest, give sources of data and details of methods of assessment (measurement).<br>Describe comparability of assessment methods if there is more than one group	Methods (page 7-8)		
Bias	9	Describe any efforts to address potential sources of bias	Methods (page 7-8)<br>Discussion (page 12)		
Study size	10	Explain how the study size was arrived at	Methods (page 7-8)		
Quantitative variables	11	Explain how quantitative variables were handled in the analyses. If applicable, describe which groupings were chosen, and why	Methods (page 7-8)		
Statistical methods	12	(a) Describe all statistical methods, including those used to control for confounding<br>(b) Describe any methods used to examine subgroups and interactions<br>(c) Explain how missing data were addressed	Methods (page 7-8)<br>Pre-specified analysis plan (supplemental data)<br>Supplemental methods		

		(d) *Cohort study* - If applicable, explain how loss to follow-up was addressed<br>*Case-control study* - If applicable, explain how matching of cases and controls was addressed<br>*Cross-sectional study* - If applicable, describe analytical methods taking account of sampling strategy<br>(e) Describe any sensitivity analyses			
Data access and cleaning methods		..	Methods (page 7-8)<br>Pre-specified analysis plan (supplemental data)<br>Supplemental methods	RECORD 12.1: Authors should describe the extent to which the investigators had access to the database population used to create the study population.<br><br>RECORD 12.2: Authors should provide information on the data cleaning methods used in the study.	Methods (page 7-8)<br>Pre-specified analysis plan (supplemental data)<br>Supplemental methods
Linkage		..		RECORD 12.3: State whether the study included person-level, institutional-level, or other data linkage across two or more databases. The methods of linkage and methods of linkage quality evaluation should be provided.	Methods (page 7-8)<br>Pre-specified analysis plan (supplemental data)<br>Supplemental methods
**Results**					

Participants	13	(a) Report the numbers of individuals at each stage of the study (*e.g.*, numbers potentially eligible, examined for eligibility, confirmed eligible, included in the study, completing follow-up, and analysed)<br>(b) Give reasons for non-participation at each stage.<br>(c) Consider use of a flow diagram	Non applicable	RECORD 13.1: Describe in detail the selection of the persons included in the study (*i.e.,* study population selection) including filtering based on data quality, data availability and linkage. The selection of included persons can be described in the text and/or by means of the study flow diagram.	Whole population therefore non-applicable
Descriptive data	14	(a) Give characteristics of study participants (*e.g.*, demographic, clinical, social) and information on exposures and potential confounders<br>(b) Indicate the number of participants with missing data for each variable of interest<br>(c) *Cohort study* - summarise follow-up time (*e.g.*, average and total amount)	Results (page 10-11)<br>Supplemental tables<br>Discussion (page 12)		
Outcome data	15	*Cohort study* - Report numbers of outcome events or summary measures over time<br>*Case-control study* - Report numbers in each exposure category, or summary measures of exposure<br>*Cross-sectional study* - Report numbers of outcome events or summary measures	Non-applicable		
Main results	16	(a) Give unadjusted estimates and, if applicable, confounder-	Results, supplemental tables		

		adjusted estimates and their precision (e.g., 95% confidence interval). Make clear which confounders were adjusted for and why they were included<br>(b) Report category boundaries when continuous variables were categorized<br>(c) If relevant, consider translating estimates of relative risk into absolute risk for a meaningful time period			
Other analyses	17	Report other analyses done—e.g., analyses of subgroups and interactions, and sensitivity analyses	Results (page 10-11)		
**Discussion**					
Key results	18	Summarise key results with reference to study objectives	Discussion (page 12)		
Limitations	19	Discuss limitations of the study, taking into account sources of potential bias or imprecision. Discuss both direction and magnitude of any potential bias	Discussion (page 12 and 13)	RECORD 19.1: Discuss the implications of using data that were not created or collected to answer the specific research question(s). Include discussion of misclassification bias, unmeasured confounding, missing data, and changing eligibility over time, as they pertain to the study being reported.	Discussion (page 12-13)
Interpretation	20	Give a cautious overall interpretation of results considering objectives, limitations, multiplicity of analyses, results from similar studies, and other relevant evidence	Discussion (page 13)		

Generalisability	21	Discuss the generalisability (external validity) of the study results	Discussion (page 13)		
**Other Information**					
Funding	22	Give the source of funding and the role of the funders for the present study and, if applicable, for the original study on which the present article is based	Title (page 3)<br>Methods (page 9)		
Accessibility of protocol, raw data, and programming code		..		RECORD 22.1: Authors should provide information on how to access any supplemental information such as the study protocol, raw data, or programming code.	Data sharing statement (page 2)<br>Methods (page 8)

*Reference: Benchimol EI, Smeeth L, Guttmann A, Harron K, Moher D, Petersen I, Sørensen HT, von Elm E, Langan SM, the RECORD Working Committee. The REporting of studies Conducted using Observational Routinely-collected health Data (RECORD) Statement. *PLoS Medicine* 2015; in press.

*Checklist is protected under Creative Commons Attribution (CC BY) license.
