# Supplementary material for: Uptake of infant and preschool immunisations in Scotland and England during the COVID-19 pandemic: An observational study of routinely collected data
Source: PLoS Med. 2022 Feb 22;19(2):e1003916. doi: 10.1371/journal.pmed.1003916 (PMC8863286; doi:10.1371/journal.pmed.1003916)
Supplement: Attachment — Submitted filename: Response to reviewers only.docx [file pmed.1003916.s016.docx]

**PMEDICINE-D-21-03163R1 Response to reviewer comments**

**Comments from the academic editor**

As the reviewers indicate, there is a need of data and descriptions of the secondary effects of the pandemic on health services, coverage and health outcomes. A possible increase in coverage is of interest among all other reports of negative indirect effects - although it may be difficult to draw inferences that are relevant to other settings.

**Comments from the editor**

**General comments:**
Throughout the paper, please adapt reference call-outs to the following style: "... likely to be profound [7,8]." (noting the absence of spaces within the square brackets).

- The reference call-outs have been adapted as requested.

**Data availability:**
PLOS Medicine requires that the de-identified data underlying the specific results in a published article be made available, without restrictions on access, in a public repository or as Supporting Information at the time of article publication, provided it is legal and ethical to do so.
Where the data are not freely available, please describe briefly the ethical, legal, or contractual restriction that prevents you from sharing it. Please also include an appropriate contact (web or email address) for inquiries - please note that a study author cannot be the contact person for data requests.

- The data sharing statement and methods have been updated:

Line 33 “All Scottish data used in this study are publicly available on the Public Health Scotland “Wider impacts of COVID-19” dashboard which can be accessed via <https://scotland.shinyapps.io/phs-covid-wider-impact/>. The English data used is included in the supplementary information. Any further queries regarding the data on ImmForm should be directed to [EarlyChildhoodBaseline@phe.gov.uk](mailto:EarlyChildhoodBaseline@phe.gov.uk). The code used to analyse the data is publicly available via the EAVE II GitHub (<https://github.com/EAVE-II)>.”

And

Line 244 “Scottish data can be accessed via the PHS wider impacts dashboard <https://scotland.shinyapps.io/phs-covid-wider-impact/> . The English data used is included in the supplementary information and any further queries should be directed to [EarlyChildhoodBaseline@phe.gov.uk](mailto:EarlyChildhoodBaseline@phe.gov.uk).

- The use of English data from Immform is in accordance with the following statement:

“The data on ImmForm contains results collected from national surveys and collections. Any secondary analyses of unpublished data should apply acceptable standards and guidance on data quality relating to the collection, analysis, publication or other dissemination of data and information. Any secondary analyses of unpublished data should be well described and obtained using sound methodology and where possible produced in conjunction with official statistics/data already in the public domain. Please ensure you have considered all caveats when interpreting the data and reference the source in any subsequent reproductions or analyses of data. Pre-release access is subject to terms protecting patient privacy and confidential information in relation to the legitimacy of the data request for the purpose of the effective discharge of official functions and research.

The user agrees:

- not to disclose or share without prior agreement, any part of the data that includes unpublished data until official statistics have been published
- not to use access for personal gain or take any action for political advantage
- not to use access to change or compromise the content, presentation or the timing of publication of the data
- use of the data must be consistent with the condition of supply
- to suppress numbers less than five in order to prevent deductive disclosure of information about individuals, which directly or indirectly leads to the identification of any patient, as set out in guidelines issued by the Information Commissioners Office (ICO)

**NB** Your organisation, UK Health Security Agency (UKHSA), The Department of Health and Social Care (DHSC), NHS England shall not publish any information which directly or indirectly leads to the identification of any patient as set out in guidelines issued by the ICO. The ICO guidelines relating to anonymisation of patient data are found at: <https://ico.org.uk/media/for-organisations/documents/1061/anonymisation-code.pdf>. Nor should data be published that leads to the identification of individual practices as consent for this from all practices has not been obtained.

- the sharing of data with third parties (following approval by UKHSA) should follow the ICO guidelines and parties should ensure that that they have a data sharing agreement in place. The ICO guidelines relating to sharing of data can be found at: [https://ico.org.uk/media/for-organisations/documents/1068/data_sharing_code_of_practice.pdf](https://eur01.safelinks.protection.outlook.com/?url=https%3A%2F%2Fico.org.uk%2Fmedia%2Ffor-organisations%2Fdocuments%2F1068%2Fdata_sharing_code_of_practice.pdf&data=04%7C01%7CElise.Tessier%40phe.gov.uk%7C8d13643a9b8c4c5738d008d993ab55ba%7Cee4e14994a354b2ead475f3cf9de8666%7C0%7C0%7C637703186636781792%7CUnknown%7CTWFpbGZsb3d8eyJWIjoiMC4wLjAwMDAiLCJQIjoiV2luMzIiLCJBTiI6Ik1haWwiLCJXVCI6Mn0%3D%7C3000&sdata=t3xFTQs9ceRqzm9rVkJTcOEN64wVzLwNmW0H5W2H2tE%3D&reserved=0)

If you have any further queries regarding the data on ImmForm please contact [EarlyChildhoodBaseline@phe.gov.uk](mailto:EarlyChildhoodBaseline@phe.gov.uk)”

**Abstract Background:** The final sentence should clearly state the study question.

- Line 46 “The aim of this study was to use these data to assess the impact of different phases of the pandemic on infant and preschool immunisation uptake rates.”

**Abstract Methods and Findings:**
Please provide brief demographic details of the study population (e.g. sex, age, ethnicity, etc)

- The issue of demographic data has been further addressed in our response to the first “Results” comment below.

Please include the number of participants and dates between which the study took place.

- Lines 49 “Observational study using routinely collected data for the year prior to the pandemic (2019), and immediately before (January-22^rd^ March 2020), during (23^rd^ March-26^th^ July) and after (27^th^ July-4^th^ October) the first UK ‘lockdown’.”
- Lines 56/57 “This represented 439,754 invitations to be vaccinated in Scotland and 4.1million for England.”

Please define abbreviations at first use e.g. DTaP/IPV/Hib/HepB, MMR

- Line 55-56 “Five vaccinations delivered at different ages were evaluated; three doses of the ‘6-in-1’ Diphtheria, Tetanus, Pertussis, Polio, *Haemophilus influenzae* type b and Hepatitis B vaccine (DTaP/IPV/Hib/HepB) and two doses of Measles, mumps and Rubella vaccine (MMR).”

Please clarify whether CIs presented are 95% CIs. Where ORs are provided, please specify the comparison group.

- This has been clarified for each results quotes. For example, line 65 “(95.3 vs 94%, OR compared to 2019 1.28, 95% CI 1.18-1.39)”

In the last sentence of the Abstract Methods and Findings section, please describe 2-3 of  the main limitations of the study's methodology.

- Lines 73-75: “The use of routinely collected data used in this study was a limiting factor, as detailed information on potential confounding factors were not available and we were unable to completely eliminate the possibility of seasonal trends in immunisation uptake.

**Abstract Conclusions:**
Please begin your Abstract Conclusions with "In this study, we observed ..." or similar, to summarize the main findings from your study, without overstating your conclusions. Please emphasize what is new and address the implications of your study, being careful to avoid assertions of primacy.
Please avoid using ‘effect’, which should be used only if causality can be inferred, i.e., from an RCT.

Lines 77-82 “In this study, we observed that the national lockdown in Scotland was associated with an increase in timely childhood immunisation uptake, however in England uptake fell slightly. Reasons for the improved uptake in Scotland may include active measures taken to promote immunisation at local and national levels during this period and should be explored further. Promoting immunisation uptake and addressing potential vaccine hesitancy is particularly important given the ongoing pandemic and COVID-19 vaccination campaigns.”

**Author Summary:**
At this stage, we ask that you include a short, non-technical Author Summary of your research to make findings accessible to a wide audience that includes both scientists and non-scientists. The Author Summary should immediately follow the Abstract in your revised manuscript. This text is subject to editorial change and should be distinct from the scientific abstract. Please see our author guidelines for more information: <https://journals.plos.org/plosmedicine/s/revising-your-manuscript#loc-author-summary>
In the final bullet point of ‘What Do These Findings Mean?’, please describe the main limitations of the study in non-technical  language.

- Lines 84-115:

“**Why was this study done?**

- Early reports from multiple countries suggested that the COVID-19 pandemic and associated control measures such as ‘lockdowns’ could be detrimental to routine childhood immunisation uptake.
- A fall in the number of children receiving their routine immunisations could leave the population vulnerable to multiple infectious diseases such as measles.
- However, these reports generally only assessed the immediate effect within a few weeks of national lockdowns and the longer-term impact of COVID-19 control measures on routine childhood immunisation uptake has not yet been fully evaluated.
- It is important to understand how the pandemic affected the uptake of routine childhood immunisations in order to learn lessons about what factors influence immunisation uptake and help plan for the future.

**What did the researchers do and find?**

- Information on childhood immunisation uptake is routinely collected in Scotland and England. Because of concerns about the effect of the pandemic on immunisation uptake, this information was collected in more detail and we used this to examine what happened over the lockdown period.
- To see if there was any change in immunisation uptake, we compared the information from immediately before, during and after the 2020 lockdown in the United Kingdom to information from the previous year (2019).
- We found that immunisation uptake in Scotland rose significantly across lockdown, with over seven thousand more children receiving their immunisations on time compared to the previous year. In England there was a slight fall in uptake.

**What do these findings mean?**

- These findings suggest that, despite early concerns, infant and preschool immunisation uptake increased in Scotland over the lockdown period.
- We don’t yet have enough information to determine the reasons behind this positive change in Scotland or why uptake fell in England.
- The next step is to assess what factors may have led to this apparent increase in uptake which could improve uptake of infant and pre-school immunisations beyond the pandemic.

**Introduction:**
Line 104 - please temper assertions of primacy by adding ‘to the best of our knowledge’ or similar

- This has been amended, line 142-143: “though to the best of our knowledge the full impact has not yet been assessed.”

**Methods:**
Thank you for appending your analysis plan. Please cite this early in the Methods section. Changes in the analysis-- including those made in response to peer review comments-- must be identified as such in the Methods section of the paper, with rationale. If a reported analysis was performed based on an interesting but unanticipated pattern in the data, please be clear that the analysis was data-driven.

- The analysis plan has been referenced in the first methods paragraph and we have listed changes to the original analysis plan as requested.

Line 169 onwards:

“The prespecified analysis plan is included within the supplementary materials (S1 file). Originally, we had hoped to analyse data from the four nations of the United Kingdom, however it was only possible to obtain data from Scotland and England. Minor changes were made to the planned visualisation format of the data (from bar to line plots) to better illustrate the findings.”

Please add the following statement, or similar, to the Methods: "This study is reported as per the REporting of studies Conducted using Observational Routinely-collected Data (RECORD) guideline (S1 Checklist)." When completing the checklist, please use section and paragraph numbers, rather than page numbers which will likely no longer correspond to the appropriate sections after copy-editing.

- Line 250-251: “This study is reported as per the REporting of studies Conducted using Observational Routinely-collected Data (RECORD) guideline (S3 File) [20].” The checklist updated as requested (S3 file).

Line 205 - please refer to the specific supplementary file where further details can be found.

- Line 241: “Further methodological details can be found in S2 File.”

**Results:**
Please provide a table showing the baseline characteristics of the study population (as Table 1).
Please report the number of individuals included in your study.

- The routinely collected whole country population data used in this study do not include any further information on baseline characteristics (eg ethnicity, sex), which we have tried to acknowledge in the discussion. To address this request, we have included an additional supplementary table (Table S1) with birth data taken from the National Office of Statistics for Scotland and England. While these data do not correspond directly to the individuals in our study, they give an overview of the population and we feel it is reasonable to expect this broadly represents the characteristics of our study population. We have tried to explain these limitations in the manuscript and legend of the table. Given this, we feel it is more appropriate to include this table in the supplementary materials rather than in the main manuscript. S1 Table and line 258 “Detailed demographic data are not routinely collected and were therefore not available. For context, S1 Table shows population data from the relevant national records offices for births in Scotland and England by sex, maternal age and maternal country of birth from 2015 to 2020. It is expected that these data are broadly representative of the children included in this study.”

Please clarify whether CIs presented are 95% CIs.
Where ORs are presented, please specify the comparison group.

- Line 263: “All confidence intervals presented are 95% confidence intervals and odds ratios are compared to the baseline year 2019 unless otherwise stated.”

**Discussion:**
Please present and organize the Discussion as follows: a short, clear summary of the article's findings; what the study adds to existing research and where and why the results may differ from previous research; strengths and limitations of the study; implications and next steps for research, clinical practice, and/or public policy; one-paragraph conclusion.
Please remove all subheadings within your Discussion e.g. Limitations and other considerations

- The discussion has been rewritten and restructured as requested.

**Figures:**
Please define all abbreviations used in the figure legend of each figure.
Please consider avoiding the use of red and green in order to make your figure more accessible to those with colour blindness.
Fig. 1, Fig S1, Fig 3, Fig S5  - Please clarify what is meant by the time in brackets on the label of your y axis.

- Abbreviations have now been defined for each legend. Figure 2 colour scheme has been changed to a colour-blind accessible palette. We have amended the labels of Fig 1, S1, 3 and S5 to clarify.

**Tables:**
When a p value is given, please specify the statistical test used to determine it in the table legend.
Please define all abbreviations used in the table legend of each table.

- Table legends have been amended as requested.

**References:**
Please ensure that journal name abbreviations match those found in the National Center for Biotechnology Information (NCBI) databases, and are appropriately formatted and capitalised.
Please also see <https://journals.plos.org/plosmedicine/s/submission-guidelines#loc-references> for further details on reference formatting. For example, 6 authors should be named prior to ‘et al’.

- The references have been adjusted as requested.

**Supplementary files:**
Please see <https://journals.plos.org/plosmedicine/s/supporting-information> for our supporting information guidelines.

- The supplementary files have been amended and saved as per the above guidelines.

**Reviewer 1**

**Major Issue**
1.      The authors describe that they use "aggregate binary logistic regression" with "vaccination status (vaccinated vs unvaccinated) as the dependent variable" (lines 192-94). It is unclear to me the definition of this method and the exact formulation used. If aggregate percentages were used, how were these values weighted in the analysis? Please provide additional detail so that this analysis could be replicated.

- We have explained the supplemental methods (S2 file) to include a detailed description of how the analysis was conducted, including the R codes used (the full code will also be made available on publication). The aggregate binary regression model takes the number of events and the number of non-events as the input. Meaning it calculates the outcome using the raw counts, not percentages and therefore is already weighted.
- File S2:

***“Statistical methods details***

We used aggregate binary logistic regression to compare the probability of being vaccinated in 2019 with each of the three lockdown time-periods. Each immunisation type was analysed separately.

The explanatory variable was time-period, categorised as: 2019, pre-lockdown, lockdown and post lockdown, and the dependent variable was vaccination status (vaccinated or not vaccinated).

The data for each time-period were aggregated, that is the numbers of vaccinated and unvaccinated children (rather than percentages) for each period were combined to form a regression table for analysis. Analysis tables were set up in the example, table 1:

**Table 1: Aggregate data example for First 6in1 immunisation**

| Lockdown.factor  (Column 1) | Date  (Column 2) | Vaccinated  (Column 3) | Unvaccinated  (Column 4) |
| --- | --- | --- | --- |
| 2019 | 2019 | 47567 | 3042 |
| Pre-lockdown | January 2020 | 3393 | 200 |
| Pre-lockdown | February 2020 | 4079 | 246 |
| Pre-lockdown | W/B 02-MAR-20 | 813 | 63 |
| Pre-lockdown | W/B 09-MAR-20 | 883 | 62 |
| Pre-lockdown | W/B 16-MAR-20 | 935 | 87 |
| Lockdown | W/B 23-MAR-20 | 846 | 62 |
| etc | etc | etc | etc |

A generalised linear model was set up using the generalised linear model, glm(), function in R, coded as follows;

model_first6in1_scotland = glm(First6in1_regression_tbl ~ First6in1_LDperiod$lockdown.factor,

family="binomial")

where First6in1_regression_tbl is equivalent to columns 3 and 4 and First6in1_LDperiod$lockdown.factor to column 1 in table 1.

The odds ratios and 95% confidence intervals were obtained by calculating the exponent of the resulting coefficients/confidence intervals from the model, using the following code:

exp(model_first6in1_scotland$coefficients)

exp(confint(model_first6in1_scotland))

In order to compare whether the change in immunisation uptake varied by SIMD quintile, analysis tables were set up as per the following example in table 2:

**Table 2: SIMD data example for First 6in1 immunisation**

| Lockdown.factor  (Column 1) | SIMD  (Column 2) | Vaccinated  (Column 3) | Unvaccinated  (Column 4) |
| --- | --- | --- | --- |
| 2019 | 1 | 11025 | 971 |
| 2019 | 2 | 9740 | 721 |
| 2019 | 3 | 8457 | 573 |
| 2019 | 4 | 9804 | 447 |
| 2019 | 5 | 8442 | 327 |
| Pre-lockdown | 1 | 2383 | 197 |
| Pre-lockdown | 2 | 2119 | 170 |
| etc | etc | etc | etc |

An interaction model was created which included time point and SIMD quintile as follows:

model_SIMD_First6in1<- glm(First6in1_interaction_tbl ~ tp*SIMD, family="binomial")where First6in1_interaction_tbl is equivalent to columns 3 and 4, tp is column 1 and SIMD is column 2. The baseline comparisons were set as 2019 and SIMD 1. From this model, the ratio of odds ratio was calculated by taking the exponential function of the coefficient of the interaction term from the interaction model. If the 95% confidence intervals did not include 1, the interaction of time period and SIMD was considered statistically significant, that is; there was a significant difference in the level of change (2019- time period) between the deprivation quintile and SIMD 1. Further explanation of the interaction model can be found in [1].

***References***

- [1] Harrison E. and Pius R. Logistic Regression. In Harrison E. and Pius R. R for Health Data Science. Chapman and Hall/CRC. 2020. Also available from https://argoshare.is.ed.ac.uk/healthyr_book/”

**Minor Issues**
2.      In Figure 1 the authors provide the annual uptake for 2019, monthly uptake for Jan and Feb of 2020, and weekly update for March to September 2020. While this plot is helpful, it masks any seasonal variation that may be expected in vaccine delivery. It would be helpful to see the monthly or weekly pattern of vaccine delivery in 2019 to compare to what was observed in 2020. The authors also mention this in their limitations (lines 354-59); this analysis fails to account for seasonality in vaccine delivery.

- We agree that any seasonal variation cannot be fully excluded in this analysis and have amended our abstract and discussion to emphasis this: line 452 “In using the mean percentage of the entire year 2019 as our baseline comparator, we potentially run the risk of confusing normal seasonal variation in immunisation uptake with the impact of lockdown measures. Ideally, direct weekly or monthly comparisons would be made between 2019 and 2020, though these data are not available. The enhanced detail and frequency of collection of immunisation uptake data and rapid dissemination (through the COVID dashboard) was in response to the pandemic and therefore only began in March 2020. However, quarterly trends published for previous years including 2019 do not show major difference in uptake throughout the year and in fact show a gradual decline in uptake year on year since 2015 [30]. In addition, it is possible that 2020 uptake rates have been underestimated, due to a lag in data entry into SIRS which would particularly affect the ‘catch-up’ rates (Fig 4A).”
- The enhanced detail and frequency of collection of immunisation uptake data and rapid dissemination (through the COVID dashboard) was in response to the pandemic and therefore only began in March 2020. Prior to the pandemic, data were collected quarterly only therefore, weekly and monthly data are not available for 2019. These quarters do not align fully with our ‘lockdown’ periods and report uptake by six months of age (rather than within 4 weeks of eligibility) therefore we chose to use the whole of 2019 as our comparator. However, as mentioned in the discussion, there are no obvious seasonal trends seen in the previous quarterly data.

Despite this limitation, we hope that the overall message remains that immunisation uptake in Scotland did not fall during lockdown, as had been feared, and there may be some aspects of the lockdown that contributed to an increase in the number of children receiving their immunisation on time.

3.      The y-axis on figure 4 makes it very hard to notice the small differences in the bars. Could you use a smaller y-scale (for example 75 to 100) or a different type of chart to visualize this data?

- We had initially used the full 0-100 scale to avoid exaggerating the very small differences between the groups, however we have now amended the scale as suggested.

4.      This manuscript provides consistent and convincing data to suggest that vaccine delivery significantly increased during the COVID-19 lockdown in Scotland. The authors also acknowledge that the nature of this data do not allow for detailed understanding of the precise reasons for that trend. For these results to be most useful, it would be important for clinicians and policy-makers to understand the precise drivers of success to apply them in settings where COVID-19 continues to disrupt routine health services or in order to build more robust and resilient vaccine delivery systems in other countries. The authors provide two primary hypotheses—A) that flexible work increased appointment attendance (lines 379-83) and B) that accessibility and pre-appointment reminders increased uptake (lines 384-93). It would be helpful to supplement this analysis with qualitative or contextual data to support these hypotheses or provide additional strategies that led to the success
observed. I would also like to know if the strategies described in B) were unique to the COVID-19 lockdown period? What was the timeline for these interventions? The authors also provide some hypotheses for the decrease in England (lines 394-405) but do not explain why these factors were abated in Scotland. What were the specific program differences between England and Scotland that may have led to these differences in uptake?

- We agree that answering these questions and determining the factors which led to the apparent increase in vaccine delivery (in Scotland) is key to maintaining this success. Thus far, these hypotheses are based mainly on our experience as practitioners and the work published by Jarchow- MacDonald et al reference in the Discussion (2020). To our knowledge, the pre-appointment symptom screening phone calls were introduced during the COVID-19 lockdown, however different geographical areas may have used telephone reminders prior to the pandemic. We plan to investigate these potential explanations in detail in future and now reference this in the revised Discussion:

Line 434 “More work is required to fully dissect the key factors in improving timely

preschool immunisation uptake in Scotland. Clarity and publicity about the continuation of the immunisation programmes, telephone reminders and the opportunity to discuss with healthcare professions seem likely to have had the most impact. We plan to use the data generated in this study to inform future work investigating these factors, including geographical differences and timing introducing policies such as reminders and publicity campaigns.”

5.      The authors acknowledge in the results that the positive impact on vaccine uptake in Scotland was primarily in the "timeliness" of vaccines and that "the impact on longer term or final achieved uptake was more variable, possibly reflecting a ceiling effect on maximal uptake, for the earliest immunisations" (294-307). Based on this description and the results of this analysis, it appears that the COVID-19 lockdown may have promoted more prompt vaccinations but not overall vaccine coverage. Could the authors provide a quantitative analysis on the impact of prompt vaccination? Especially during the COVID-19 pandemic when pandemic-related behavior change such as reduced physical contact likely decreased the transmission of many infectious diseases, is vaccination a few weeks or even months earlier likely to have a big impact on the transmission of vaccine-preventable diseases?

- This is a very interesting question which we considered ourselves when designing this project. Unfortunately, it is not one we are able to answer with the data available. As noted by reviewer 1, any observed changes in vaccine-preventable diseases (for example pertussis in young babies) would be heavily confounded by the changes in behaviour such as social distancing and isolation for symptoms such as cough. However, we do hope to continue and expand on this work. Of the five vaccinations, there did appear to be an increase in overall coverage for the second MMR, therefore monitoring trends in measles cases as we move fully out of COVID-related restrictions may be of interest, though we would not be able to infer causation. We have included reference to this in the revised discussion:

Line 477 onwards: “In addition, we have not yet assessed the potential impact of improved early immunisation uptake on rates of vaccine preventable diseases. Any observed changes in vaccine-preventable diseases (for example pertussis in young babies) would be heavily confounded by the changes in behaviour such as social distancing and isolation for symptoms such as cough. However, of the five vaccinations, there did appear to be an increase in overall coverage for the second MMR, therefore monitoring trends in measles cases as we move fully out of COVID-related restrictions may be of interest, though we would not be able to infer causation.”

**Reviewer 2**

1. The results could be potentially biased when the underlying trend and seasonal patterns (e.g. if there is an increasing trend in the uptake, and or the update proportion decrease in the holiday season in December) are not controlled.  Since weekly and/or monthly data are available, I would recommend to extend the study period (e.g. Add data of 2017 and 2018 ) and employ a more robust interrupted time series design in order to control for the underlying secular trend and seasonal variation in the pre-Covid-19 period.  Although the author points out in the limitation that quarterly trends published for previous years including 2019 do not show major difference in uptake throughout the year and in fact show a gradual decline in uptake since 2015, I would suggest to analyze and graphically demonstrate the trend of the weekly/monthly uptake proportion for the years before the pandemic.

- We agree that any seasonal variation cannot be fully excluded in this analysis and have amended our abstract and discussion to emphasis this. We have also addressed this issue in our response to reviewer 1, point 2. The enhanced detail and frequency of collection of immunisation uptake data and rapid dissemination (through the COVID dashboard) was in response to the pandemic and therefore only began in March 2020. Prior to the pandemic, data were collected quarterly only therefore, weekly and monthly data are unfortunately not available for 2019. These quarters do not align fully with our ‘lockdown’ periods therefore we chose to use the whole of 2019 as our comparator. However, as mentioned in the discussion, there are no obvious seasonal trends seen in the previous quarterly data. Despite this limitation, we hope that the overall message remains that immunisation uptake in Scotland did not fall during lockdown, as had been feared, and there may be some aspects of the lockdown that contributed to an increase in the number of children receiving their immunisation on time.

2.      The author talked little about how they handle missing data. As shown in Figure 2, missingness (or does not missingness here represents not significant change? The author needs to clarify this) mainly exited in regions with higher baseline uptake proportion. This missingness not at random may confound the overall estimates.

- We acknowledge that Figure 2 was very confusing and have redrawn it to clarify that the grey areas represent non-significant change and there is no missing data. We have added a sentence in the methods and Figure 2 legend, signposting the reader to the additional methods in supplemental file S2 which gives more detail on inclusion and exclusion criteria; line 306 “Note that in Grampian, the second MMR dose is delivered at a later age therefore data for this area is recorded as ‘non-applicable’ (further details in S2 File)”.

3.      I would suggest to the title of Fig 2 because it not only shows the baseline mean update proportion. What's the difference between "Absolute % change" and "% point change" ?

- We are grateful to reviewer 2 for pointing out this error and have corrected the figure.

4.      For figure 4, I would suggest to reset the limits of y axis or present the % of unvaccinated as the difference between the four periods in the current figure is hardly noticeable.

- The figure has been redrawn as noted in the comments above.

5.      For figure S1, I would suggest to add the geographic areas with missing data.

- We have tried to clarify the issues of missing data in our response to point 2 above. All Health and Social Care Partnerships are included in figure S1, with the exception on Grampian HSCP for second dose MMR only as discussed above.

6.      The code is not available from the Github page. This needs to be updated accordingly.

- The page will be updated shortly.

7.      The author may consider sensitivity analysis examining uptake within 2 and 6 weeks of eligibility

- Unfortunately, we do not have these data available. We have clarified in the manuscript the limitations of data availability given the use of routinely collected national data. Line 454: “Ideally, direct weekly or monthly comparisons would be made between 2019 and 2020, though these data are not available. The enhanced detail and frequency of collection of immunisation uptake data and rapid dissemination (through the COVID dashboard) was in response to the pandemic and therefore only began in March 2020. However, quarterly trends published for previous years including 2019 do not show major difference in uptake throughout the year and in fact show a gradual decline in uptake year on year since 2015 [30].”

**Reviewer 3**

(I have listed the comments from the annotated manuscript below.)

1. You don't mention this again. Did enhanced surveillance have any impact on estimated coverage rates?
   - The enhanced surveillance did not impact the estimated rates. We have removed this term from the revised abstract as it is somewhat ambiguous “enhanced surveillance” and have replaced it with a description of what was actually done:

line 44 “..public health bodies in Scotland and England collected national data for childhood immunisations on a weekly or monthly basis to allow for rapid analysis of trends.”

- The real-time data published on the Scottish COVID dashboard may have initially underestimated coverage due to late return of results from some areas. However, the final analysis of this paper was conducted after these data had been checked and confirmed in the same way as the 2019 data.

2. You should define "first period".
   - Line 49-51 “We undertook an observational study using routinely collected data for the year prior to the pandemic (2019), and immediately before (January-22^rd^ March 2020), during (23^rd^ March-26^th^ July) and after (27^th^ July-4^th^ October) the first UK ‘lockdown’.”
3. Levels

- This has been corrected (line 67).

4. were not assessed?
   - Changed (line 179).
5. Need to include dashed lines in your legend.
   - The notation (---) has been included in the legend along with the written description of what the dashed lines represent, line 281 “Dashed horizontal lines (---) indicated the mean uptake in 2019 of the immunisation with the corresponding colour.”
6. Table should stand alone. Need to indicated this is data from Scotland.
   - The legend has been updated (line 285).
7. Spell out. "First dose of 6in1"
   - This has now been done for all doses in this table (table 1).

The below comments all refer to figure 2:

8. These titles need to be clearer.
9. When do you switch from "% point change" to "absolute % change"?
10. Labeling grey as missing is very confusing. Need to label correctly.
11. ?? What does missing mean here?
12. Please specify this data is for Scotland.
13. A and B not described.
14. What text are you referencing to here? The figures and tables need to stand alone.
    - (Response to points 8-14 which refer to figure 2) We acknowledge that figure 2 was very confusing and we are grateful to the reviewer for highlighting the areas requiring improvement. The figure has been redrawn and relabelled to address these points and the legend has been amended.
15. For Scotland.
    - The legend has been updated (line 333)
16. B is missing
    - This has been corrected (Figure 4).
17. Strongly suggest keeping the order of the x-axis the same for all 4 graphs.
    - The x-axis has been changed to the same order as suggested (Figure S1).
18. Figures 2 and S2 aren't both needed.
    - Figure S2 has been deleted and subsequent figures relabelled.
19. This figure [S4 in the original manuscript, now amended to S3 in the revision] could be deleted.
    - We would prefer to keep this figure. While the data used to generate it are included in table S4, we feel the figure improves the accessibility of the paper by providing readers with a quick visual summary of a rather large supplemental table.
