# Supplementary material for: Uptake of infant and preschool immunisations in Scotland and England during the COVID-19 pandemic: An observational study of routinely collected data
Source: PLoS Med. 2022 Feb 22;19(2):e1003916. doi: 10.1371/journal.pmed.1003916 (PMC8863286; doi:10.1371/journal.pmed.1003916)
Supplement: Attachment — Submitted filename: PMEDICINE-D-21-03163R2 Response to reviewers.docx [file pmed.1003916.s017.docx]

**PMEDICINE-D-21-03163R1 Response to editor and reviewer comments**

**Requests from Editors:**

Abstract Methods and Findings:
Line 49 - please revise to ‘We conducted an observational study’ or similar

- This has been added.

Line 65 - please define OR and CI at first use

- Definitions have been added, line 65-66: “(95.3 vs 94%, Odds ratio [OR] compared to 2019 1.28, 95% confidence intervals [CI] 1.18-1.39)”

Author Summary:
In the final bullet point of ‘What Do These Findings Mean?’, please describe the main limitations of the study in non-technical  language.

- The final bullet point has been added as follows, line 116 onwards: “Due to the type of information used, we were unable to fully explore the possibility of immunisation uptake varying by the time of year, and some potentially important details, such as ethnicity, were not available.”

Results:
Line 269 - please revise to “14.3% for”

- This change has been made (now line 272)

Discussion:

Line 398 - please remove ‘in fact’

- This change has been made (now line 402)

Line 401 -  please remove ‘Although’ at the beginning of the sentence

- This change has been made (now line 405)

Line 433 - please revise to ‘suggesting’

- This change has been made (now line 437)

Line 464 - please revise to ‘significantly’

- This change has been made (now line 468)

Line 473 - please revise ‘vital given in’

- This has been revised to “vital given the current importance of” (line 476).

Figures:
All figures - please specify country in the legend

- All figure legend now reference the country (lines 282/2, 337, 368, 606, 610, 614/615,

Fig S1 - please indicate sections A and B within the figure itself

- The reference to sections A and B within the legend has been removed as it referred to an earlier version of this figure (line 609)

Fig S4 - dashed blue lines appear to be missing from the figure itself

- The dashed blue lines referred to an earlier version of this figure, the legend has been amended as follows (line 618): “The start and end of the lockdown period is indicated by the purple shaded area.”

Fig S2 - Please note that figures cannot be reproduced from sources that are not CC-BY. Please be sure to check the usage rights.

- Is this a reference for Fig 2 rather than Fig S2? If so, the base map is free to be used (full details from <https://www.nationalarchives.gov.uk/doc/open-government-licence/version/3/>). A further statement has been added to the Fig2 legend (line 314): “... which contains public sector information licensed under the Open Government Licence v3.0.”

Tables:
S2 Table - please specify country in the legend

- This has been amended, line 629

Comments from Reviewers:

Reviewer #1: I am satisfied with the edits made to this manuscript in response to review and believe it should be accepted for publication.


Reviewer #2: We thank the author for answering reviewers's question and revising the paper.

1. The title and legends of all Figures are missing

- These are included in the final submission

Reviewer #3: The authors addressed all of my concerns in their revised manuscript.

- We are very grateful to the reviewers for the time they have taken further reviewing our manuscript.
